# Phenylpropanoid Glycoside Analogues: Enzymatic Synthesis, Antioxidant Activity and Theoretical Study of Their Free Radical Scavenger Mechanism

**DOI:** 10.1371/journal.pone.0020115

**Published:** 2011-06-03

**Authors:** Agustín López-Munguía, Yanet Hernández-Romero, José Pedraza-Chaverri, Alfonso Miranda-Molina, Ignacio Regla, Ana Martínez, Edmundo Castillo

**Affiliations:** 1 Departamento Ingeniería Celular y Biocatálisis, Instituto de Biotecnología, Universidad Nacional Autónoma de México, Cuernavaca, Morelos, México; 2 Departamento de Biología, Facultad de Química, Universidad Nacional Autónoma de México, Cd. Universitaria, México Distrito Federal (DF), México; 3 Departamento de Materia Condensada y Criogenia, Instituto de Investigaciones en Materiales, Universidad Nacional Autónoma de México, Ciudad Universitaria, México Distrito Federal (DF), México; 4 Facultad de Estudios Superiores Zaragoza, Universidad Nacional Autónoma de México, Batalla del 5 de mayo y Fuerte de Loreto, México Distrito Federal (DF), México; University of Wales Bangor, United Kingdom

## Abstract

Phenylpropanoid glycosides (PPGs) are natural compounds present in several medicinal plants that have high antioxidant power and diverse biological activities. Because of their low content in plants (less than 5% w/w), several chemical synthetic routes to produce PPGs have been developed, but their synthesis is a time consuming process and the achieved yields are often low. In this study, an alternative and efficient two-step biosynthetic route to obtain natural PPG analogues is reported for the first time. Two galactosides were initially synthesized from vanillyl alcohol and homovanillyl alcohol by a transgalactosylation reaction catalyzed by *Kluyveromyces lactis* β-galactosidase in saturated lactose solutions with a 30%–35% yield. To synthesize PPGs, the galactoconjugates were esterified with saturated and unsaturated hydroxycinnamic acid derivatives using *Candida antarctica* Lipase B (CaL-B) as a biocatalyst with 40%–60% yields. The scavenging ability of the phenolic raw materials, intermediates and PPGs was evaluated by the 2,2-diphenyl-1-picrylhydrazyl radical (DPPH•) method. It was found that the biosynthesized PPGs had higher scavenging abilities when compared to ascorbic acid, the reference compound, while their antioxidant activities were found similar to that of natural PPGs. Moreover, density functional theory (DFT) calculations were used to determine that the PPGs antioxidant mechanism proceeds through a sequential proton loss single electron transfer (SPLET). The enzymatic process reported in this study is an efficient and versatile route to obtain PPGs from different phenylpropanoid acids, sugars and phenolic alcohols.

## Introduction

Antioxidants are one of the most important nutraceutical compounds that have emerged from the recent decades of research in food science. The advances in this field have allowed a better understanding of the free radical damage of cellular constituents, such as lipids, proteins and DNA. Antioxidants and radical scavengers have a crucial role in the treatment or prevention of several diseases such as type 2 diabetes, atherosclerosis, cancer, cardiovascular disorders and neurodegenerative disorders [1].

**Figure 1:**
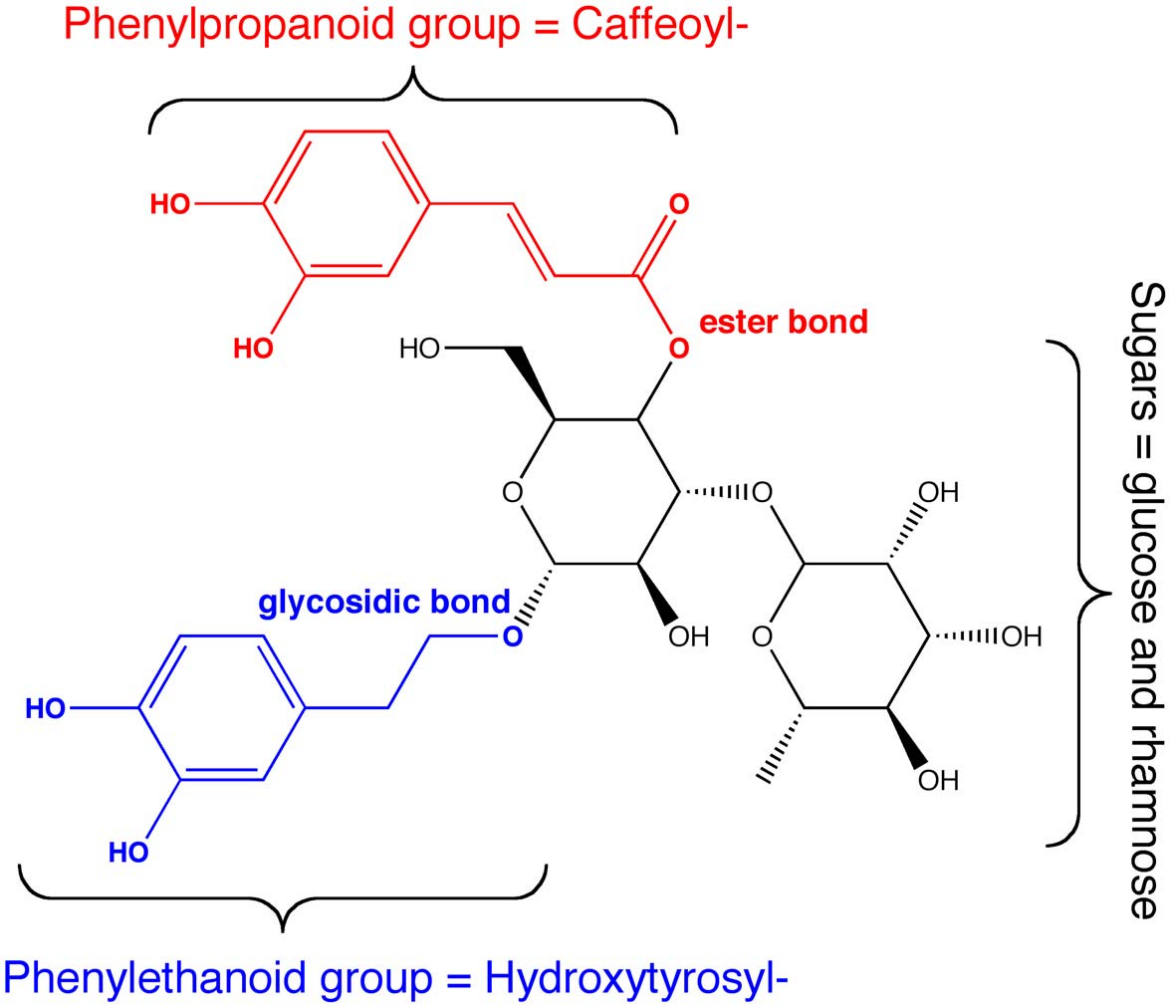
Verbascoside (Acteoside) is a PPG with applications in food and pharmaceutical sciences. The PPG molecule contains one or more sugar molecules and two antioxidants linked through an ester and glycosydic linkages.

Phenylpropanoid glycosides (PPGs) are acylated glycoconjugates carrying a substituted arylalkyl aglycon, the acylation occurring mainly on the primary hydroxyl group with a cinnamoyl derived residue. PPGs are secondary metabolites widely distributed in plants with demonstrated therapeutical properties against hypertension, viral infections, fungal infections, tumors and cancer as well as an immunomodulatory effect [2]–. These properties have been related with the antioxidative and free radical scavenging capacities of their structural components [1]–[2], [7]. In fact, several *in vitro* assays have demonstrated that PPGs act as potent antioxidants by inhibiting the oxidation of low-density lipoproteins through different mechanisms such as free radical scavenging and metal ion chelation, associated with the presence of phenylpropanoid and phenylethanoid groups in their structure (Figure 1) [2]–[8].

Despite the interesting properties of PPGs as therapeutic agents, antioxidants or free radical scavengers, the low content of PPGs in plants (less than 5% w/w) has limited their isolation and commercial application [9]. Phenylpropanoid verbascoside extracted from *Aloysia triphylla* (syn. *Lippia citriodora*) is among the few PPGs commercially available (Planox-L®, Anoxymer GmbH, Germany) [4], [10]. Several chemical pathways have been described for the synthesis of PPGs. Nevertheless, the requirement of protection and deprotection steps for their synthesis, also requiring stereochemical and regiochemical reactions result in non-sustainable, low yield and time consuming processes [5], [6], [11]–[18]. During the last two decades, biocatalysis has become a valuable tool in organic synthesis due to the chemo-, regio- and stereoselectivity of the enzymatic reactions, as well as their high specificity and mild reaction conditions. As shown in Figure 1, the structural skeletons of PPGs contain glycosidic and ester groups which may be readily introduced through enzymatic synthesis. In fact, glycosidases and lipases have already been applied to the synthesis of analogous compounds requiring specific sugars or acid derivatives [19], [20]. For instance, Bridiau *et al.* (2006) have described an effective process for the synthesis of galactosides of different benzyl and phenyl ethyl derivatives by selective transgalactosylation catalyzed by β-galactosidase from *Kluy*v*eromyces lactis* [21]. Alternatively, lipases have been successfully applied in the regioselective synthesis of sugar esters of phenylpropanoid related compounds and for the preparation of hydrophilic and hydrophobic derivatives of natural phenolic acids in organic media [22]–[27]. More specifically, the comparative reactivity of phenylpropanoids such as caffeic and ferulic acids versus the corresponding dihydrophenyl propanoid acids revealed the strong influence of propanoid chain unsaturations on the transformation of these compounds by *Candida antarctica* lipase B (CaL-B) in organic solvent media [23]. According to the literature, saturated derivates of caffeic and ferulic acids have higher free radical scavenging activities than their corresponding unsaturated analogs, suggesting that this activity may be extended to the corresponding PPG analogs. However, there is no conclusive explanation of this behavior [28], [29].

**Figure 2:**
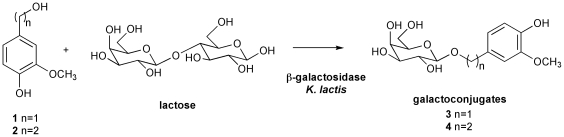
Enzymatic synthesis of galactosides 3 and 4 catalyzed by β-galactosidase from *Kluyveromyces lactis.* Acceptor substrates (250 mM), lactose (donor; 1 M) and MgCl_2_ (0.01 M) were suspended in phosphate buffer (50 mM and pH 6.5) and incubated at 35°C prior to an addition of 12 U/mL β-galactosidase.

**Figure 3:**
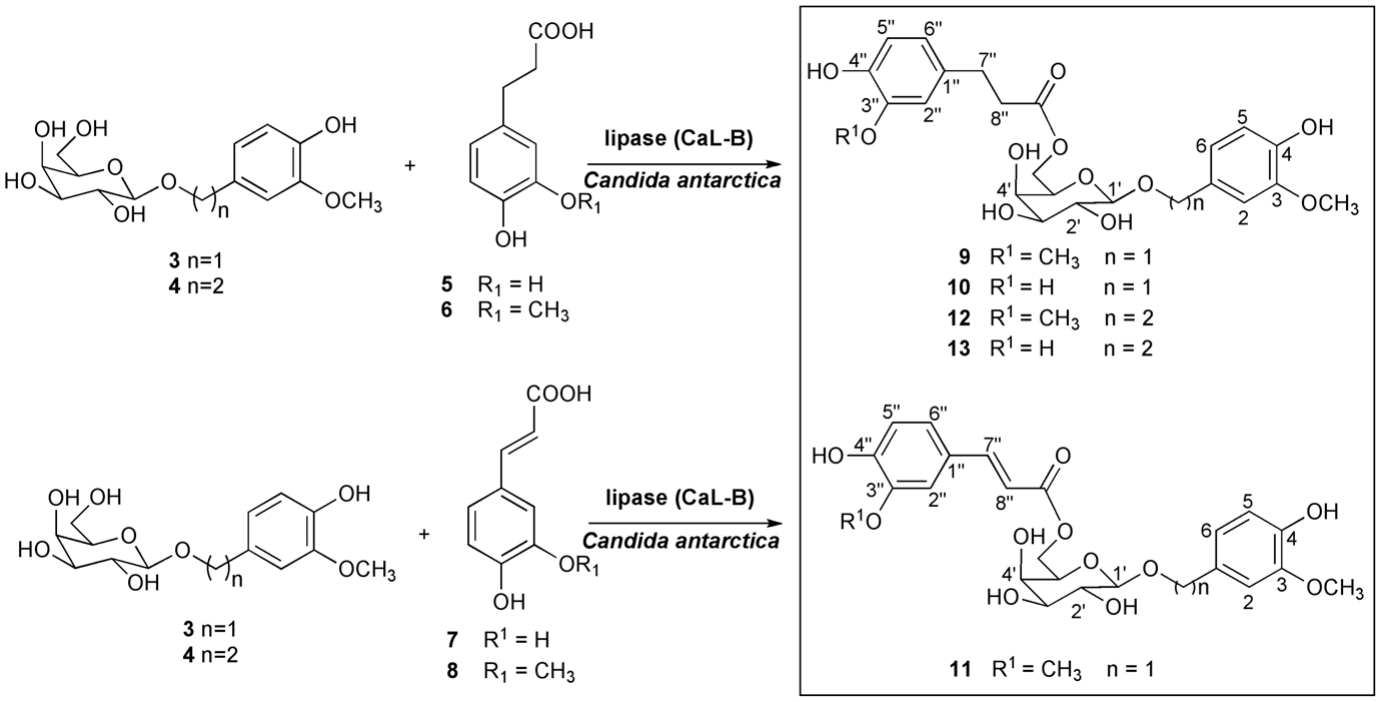
Lipase-catalyzed acylation of galactosides. 30 mM of aromatic galactosides were reacted with an equivalent amount of donor substrates with Novozym 435 (20 mg/mL) and 100 mg/mL of molecular sieves (5Å, 8–12 mesh) in 2-methyl-2-propanol. The enzymatic reaction was carried out for 24 h at 75°C with constant stirring.

In the present study, we describe an alternative and efficient two-step biosynthetic route to obtain bifunctional analogs of PPG. The first step is related to the galactosylation of vanillyl alcohol (**1**) and homovanillyl alcohol (**2**) performed with a β-galactosidase from *K. lactis* (**Figure 2**). The second step concerns to an esterification reaction between dihydroferulic or dihydrocaffeic acids and the primary hydroxyls of the galactosyl residue using CaL-B (**Figure 3**). In order to analyze if the antioxidant ability is modified in these new compounds, we investigated the effect of structural differences on the free radical scavenging ability of substrates and PPGs with the 2,2-diphenyl-1-picrylhydrazyl radical (DPPH•) assay. The structural differences included the length of the aliphatic chain in the aglycon, the presence of α-β unsaturation in the propanoid chain of the acyl residue and the number of hydroxyl groups in the aromatic rings.

Finally, potential reaction mechanisms for the antioxidant reaction were investigated through density functional theory (DFT) calculations to obtain a theoretical evaluation of the antioxidant capacity of these molecules. These predictions may be useful for further experimental design.

## Materials and Methods

### Materials

The following commercially available enzymes were used for synthesis reactions in this project: β-galactosidase from *K. lactis* (3000 LAU/ml; Maxilact, DSM Food Specialties) and immobilized CaL-B from Novozym 435 (Novozymes A/C, México city, Mexico). The following reagents were used in this study: 2-methyl-2-propanol, methanol and acetonitrile (HPLC grade) (J.T. Backer, Phillipsburg, NJ, USA); standards of vanillyl alcohol, homovanillyl alcohol and 2,2-diphenyl-1-picrylhydrazyl (DPPH•) (Aldrich, WI, USA); molecular sieves (5Å beads, 8–12 mesh), lactose and 3,4-dihydroxycinnamic acid (caffeic acid) (Sigma Chemical Co., MO, USA); and ferulic acid (Fluka Chemie, Buchs, Switzerland).

### General Experimental Procedures

HRFABMS spectra in a matrix of *m*-nitrobenzyl alcohol were recorded on a JEOL JMX-AX 505 HA mass spectrometer. All of the NMR spectra were recorded on a Varian Unity spectrometer at 400 MHz for ^1^H NMR, ^1^H-^1^H COSY, HETCOR and HMBC and 100 MHz for ^13^C NMR using CDCl_3_, CD_3_OD, pyridine-*d*_5_ and acetone-*d*_6_ as solvents. Chemical shifts are reported in parts per million (ppm) and referenced to the proton resonance of the solvent used for each sample. Low pressure column chromatography was carried out on a silica gel 60 (230–400 mesh, Macherey-Nagel). Analytical thin layer chromatography (TLC) was performed on precoated silica gel 60 F254 plates (Macherey-Nagel).

After removing the solid components of the reaction medium by filtration or centrifugation, esterification reaction products were analyzed by HPLC using a Waters 600E system controller (Waters Corp., Milford, MA) at 45°C that was equipped with a Waters 996 photodiode array detector using a Waters Spherisorb ODS-2 column (4.6 mm×250 mm, Waters Corp., Milford, MA) and a solvent mixture of water/methanol/trifluoracetic acid (60∶40∶0.2 v/v) as an eluent at a flow rate of 1 ml/min. For the galactosylation reaction, products were eluted at room temperature with acetonitrile/water (80∶20) at a flow rate of 1 ml/min separated with a Kromasil NH_2_ column (4.6 mm×250 mm) and analyzed with a refraction index detector (Waters Corp., Milford, MA).

### Synthesis of dihydroferulic and dihydrocaffeic acids

Dihydrocaffeic acid and dihydroferulic acid were synthesized according to previous reports [30]. Dihydroferulic acid was prepared by dissolving ferulic acid (15 g, 77 mmol) in 150 mL of methanol in the presence of 450 mg of Pd/C 10% as a catalyst. Hydrogenation was carried out at 60 psi for 1 h, and the reaction was monitored by TLC (*n*-hexane/ethyl acetate 65∶35). Once the reaction was completed, the catalyst was filtered, and the solvent was evaporated. The product was recovered by crystallization from methanol, and 15.1 g of product was obtained in a quantitative yield (99%). Dihydrocaffeic acid was prepared by dissolving 20 g of caffeic acid in 200 mL of an acetone/methanol (1∶1 v/v) mixture and 600 mg of Pd/C 10% as a catalyst. Hydrogenation was carried out at 60 psi for 1 h, and the end of the reaction was monitored by TLC (*n*-hexane/acetone and 1∶1, *n*-hexane/AcOEt 7∶3 with 5% of acetic acid). Once the reaction was completed, the catalyst was filtered, and the solvent was evaporated. The product was recovered by crystallization from a methyl-*tert*-butyl ether/*n*-hexane (1∶1 v/v) mixture (17 g and 85% yield). The spectral data of both products were similar to previously reported spectral data [30].

### Galactosides Synthesis and Purification

Galactosides **3** and **4** (**Figure 2**) were prepared at 35°C in a 50 mM phosphate buffer (pH 6.5) containing 0.01 M MgCl_2_ and 12 U/mL β-galactosidase using vanillyl alcohol (**1**) and homovanillyl alcohol (**2**) as acceptors (250 mM) and lactose as a sugar donor (1 M). High lactose concentrations were selected to favor transglucosylation over hydrolysis. Aliquots of the reaction mixture were withdrawn at relevant time intervals, heated for 5 min at 100°C to stop the reaction and centrifuged for 10 min at 1150 x g at 25°C to eliminate suspended solids. The products were analyzed either by TLC using acetonitrile/water/acetic acid (3∶1∶0.2 v/v/v) as a solvent or by HPLC. The residual acceptor was extracted twice with chloroform (CHCl_3_) and concentrated prior to application to silica gel column chromatography using CHCl_3_/methanol (80∶20) as an eluent (30%–35% yield). Pure galactosides (**3**–**4**) were used as standards for quantification.

### Esterification reaction of galactosides

The synthesis of PPGs (**9**–**13**, Figure 3) was prepared at 75°C in a solvent mixture of 2-methyl–2-propanol/acetonitrile (90∶10) by reacting 70 mM of the synthesized galactosides with two molar equivalents (140 mM) of the donor caffeic and ferulic acid derivatives (**5**–**8**) in the presence of 20 mg/mL Novozym 435 and 100 mg/mL of molecular sieves (5Å, 8–12 mesh). The enzymatic reaction was monitored for 24 h. The samples were withdrawn at various times and were diluted, centrifuged and analyzed by HPLC for quantification of substrates and products. The reaction was stopped by removing the biocatalyst and molecular sieves by centrifugation followed by evaporation of the solvent under vacuum. The ester products were purified by column chromatography over a silica gel eluting with ethyl acetate:ethanol (95∶5) (40%–60% yield).

### Spectral Data of Compounds

All products were synthesized and purified as above described and obtained as off-white powders. The structures of compounds were elucidated on the basis of the 1D (^1^H, ^13^C) and 2D (COSY, HETCOR and HMBC) NMR experiments. Compound **13 [**2-(4-hydroxy-3-methoxyphenyl)ethyl-6-*O*-dihydrocaffeoyl-β-D-galactopyranoside] was used as a model for structural description. All the details concerning product’s characterization are included in the Materials and Methods S1.

### Evaluation of PPGs Scavenging Effect on DPPH•

The reaction with the DPPH• is a short and efficient method widely used to evaluate the antioxidant capacity that consists of measuring the decay of visible absorption of the DPPH•, which is a stable free radical having a purple color [31]–[33]. When free radical scavengers are added, the color of the DPPH• changes to yellow due to its conversion to diphenyl-picrylhydrazine (DPPH-H). According to the method reported by Yamaguchi (1998), a reaction mixture containing 2.95 mL of 100 mM ethanolic DPPH• solution and 50 µL of sample dissolved in methanol at an adequate concentration was shaken vigorously on a vortex mixer and incubated at room temperature [34]. No differences in the IC_50_ values were found when the scavenging assay was performed at 20 or 120 min for ascorbic acid (results not shown) so 20 min were fixed for the assay. Compounds with higher IC_50_ than ascorbic acid at 20 min were not of interest for the purpose of this study. The non-reacted DPPH• was determined colorimetrically at 517 nm using a Beckman DU 650 spectrophotometer. Test compounds and changes in the absorbance of the samples were expressed in terms of IC_50_, indicating the concentration of a compound required for a 50% decrease in absorbance of the DPPH•. IC_50_ was calculated from a concentration-response curve, and ascorbic acid was used as a positive control. All analyses were carried out in triplicates.

### Computational details

Full optimizations without symmetry constraints were carried out using the hybrid, three parameter B3LYP functional within the density functional theory (DFT) framework and the 6-311G(d) basis set [35a], [35b], [36a], [36b], [36c]. Harmonic frequency analyses were used to verify the optimized minima. Single point energy calculations at these optimized geometries were computed with the same functional and 6-311+G(d) basis set to obtain vertical ionization energies (IE) and electron affinities (EA) [36a], [36b], [36c]. The thermal corrections to Gibbs free energies of the B3LYP/6-311G(d) fully optimized stationary points, and the corresponding B3LYP/6-311+G(d) single point electronic energy were used to obtain the adiabatic Gibbs free energy of each species involved in the charge transfer reaction. The stationary points were first modeled in a gas phase (vacuum), and the solvent effects were included *a posteriori* by single point calculations using the polarizable continuum model, specifically the integral-equation-formalism (IEF-PCM) at the B3LYP/6-311+G(d) level of theory with water and benzene as solvents for polar and nonpolar environments, respectively [37a],[37b],[37c]. All calculations were done with Gaussian 03 software [38]. The Gibbs free energies in solution were computed as the PCM B3LYP/6-311+G(d) single point electronic energy with the thermal corrections to Gibbs free energies from the gas phase B3LYP/6-311G(d) calculations.

## Results and Discussion

### Enzymatic synthesis of PPGs (9–13)

The availability of an enzymatic method for the synthesis of PPGs is not only attractive in terms of an efficient procedure to obtain these secondary metabolites but also because it is an interesting alternative to introduce structural variability to PPGs so that structure-function relationships may be studied. In particular, the effect on the scavenging activity of the aliphatic chain length in the aglycon, the presence of α-β unsaturation in the acyl moiety and the number of hydroxyl groups of PPGs were examined.

The first step of the biosynthesis of the bifunctional phenylpropanoid analogs consisted of the preparation of galactosides **3** and **4** by the galactosylation reaction using a high concentration of lactose (1 M) as a donor and 250 mM of **1** or **2** as acceptors (**Figure 2**). From each reaction, two compounds were detected by HPLC (retention time (rt)  = 5.7 min and rt  = 16 min) with a maximum absorption at 280 nm. After isolation, purification, quantification and structural analysis, both compounds were identified as monogalactosides **3** and **4** (30%–35% yield) and corresponding digalactosides (<5% yield). Interestingly, it was found that the galactosyl residues were completely incorporated into the primary hydroxyl groups of **1** and **2** without any incorporation in phenolic residues. These results correlate with results previously reported for the synthesis of other galactosylated compounds [21].

It has been well documented that the enzymatic transglycosylation with glycosidases is a kinetically controlled reaction performed in the context of an unfavorable thermodynamic equilibrium. In this study, the galactosylation conditions were selected after exploring a wide range of initial acceptor and donor concentrations. The concentrations were as high as allowed with the low solubility of the substrates. Furthermore, reactions completed at concentrations of lactose lower than 1 M had galactoside yields that were significantly decreased favoring the hydrolysis reaction. Moreover, all lactose was hydrolyzed at concentrations lower than 0.6 M. Although it has been stated that this behavior is related to the reduced water activity at high sugar concentrations, these results have also been described as the consequence of higher β-galactosidase specificity for lactose [39].

For the second step of PPG synthesis, immobilized CaL-B (Novozym 435) was used to acylate the synthesized galactosides **3** and **4** using hydroxycinnamoyl acid derivatives (**5**–**8**) as acyl donors. In these cases, both the acyl donors (**5**–**6)** and their corresponding unsaturated compounds (**7**–**8)** were evaluated to explore the reactivity of the acyl donors in the lipase-catalyzed acylation and their effect on the antioxidant activity of PPGs. Indeed, the high esterification yields of dihydrophenylpropanoid acids in reactions catalyzed by lipases have been recently reported [21], [40].

In previous reports, we described a thermodynamic strategy for the preferential synthesis of esters or amides as a function of solvent polarity [41], [42]. Thus, for the synthesis of PPGs **9**–**13,** dihydrocaffeic (**5**), dihydroferulic (**6**), caffeic (**7**) and ferulic acid (**8**) were reacted with galactosides **3** and **4** in polar 2-methyl-2-propanol at 75°C in the presence of CaL-B (20 mg/mL) and molecular sieves molecular sieves (5Å, 8–12 mesh), to eliminate the water produced after condensation (**Figure 3**). After 24 h, products of each reaction were identified as the corresponding esters **9**–**13**, the efficiency of acylation was higher with saturated hydroxycinammic acid derivatives: dihydroferulic acid (60% yield) and dihydrocaffeic acid (54% yield). In contrast, the efficiency of acylation resulted lower with unsaturated hydroxycinammic acid derivatives: ferulic acid (40% yield) and caffeic acid (yield <5%). From these results, it may be concluded that the electrophilicity of the carboxyl groups in α-β unsaturated systems is lower than that of the corresponding saturated compounds. It is worth mentioning that CaL-B exhibited high specificity for the acylation of the C6 primary alcohol of the galactosyl residue. To the best of our knowledge, this is the first reported enzymatic method for the synthesis of natural PPG analogues. The traditional chemical synthesis procedures result in low yields, require a considerable number of steps and require protection of the functional groups. Moreover, total yields observed for the enzymatic synthesis of PPGs (25%) were better than those previously reported and were accomplished in only two steps [5], [6], [11]–[18].

### Free Radical Scavenging Activity

The antioxidant activity of compounds **1**–**13** as defined by their capacity to scavenge the DPPH• is summarized in Table 1, in which ascorbic acid is also reported as a positive control.

**Table 1:** DPPH• antioxidant activity of compounds 1–13 and ascorbic acid as a positive control.

Compounds	IC_50_ (µM)^a^ ± SD
**1**	43.3±0.62
**2**	45.46±1.89
**3**	49.08±4.36
**4**	45.12±4.77
**5**	5.89±0.71
**6**	29.51±2.4
**7**	8.68±0.15
**8**	34.21±2.12
**9**	23.03±1.06
**10**	17.09±0.21
**11**	ND^b^
**12**	18.59±0.67
**13**	16.0±0.86
**Ascorbic acid**	20.13±0.29

a IC_50_ (µM) is the scavenging capacity and phenol concentration expressed in µM that is able to quench 50% of DPPH radicals in a 100 µM solution. Each reported value is the mean of three separate measurements. ^b^ Not determined. Structures of compounds in Figure S1.

Although it is possible to obtain absolute rate constants from a kinetic analysis of the antioxidant reaction, the values reported in Table 1 represent the global capacity to scavenge free radicals of the studied molecules and give insights of their actual reactivity. All the analyzed compounds were able to quench the free radical in a concentration-dependent way. The free radical scavenging activities of the free (**1**–**2**) or galactosylated (**3**–**4**) PPG precursors were similar allowing the conclusion that the antiradical activities of **1** and **2** were not affected by galactosylation of the molecule or by the presence of either ethyl or propyl chains on the aglycon. Concerning the antiradical activity of the compounds used as acyl donors, the caffeic and ferulic acids (**7** and **8)** had slightly lower activities than their corresponding saturated derivates (**5** and **6**). These results are in agreement with those previously reported when comparing saturated and unsaturated phenylpropanoid free acids [28], [29], [43]. Finally, the IC_50_ values for the antiradical activity of the PPG analogs (**9**–**13**) fall between 23.03 µM–16.0 µM resulting in better antiradical activity than their alcohol or phenylpropanoid precursors with an exception of caffeic (**5**) and dihydrocaffeic (**7)** acids. Even though all enzymatically synthesized PPGs are simple molecules containing only one sugar moiety in their structure, it is important to note that the IC_50_ values determined for these compounds were found to be either similar to or lower than the IC_50_ values reported for natural PPGs in some cases[44]–[46].

### Computational study of the Antioxidant Mechanisms

In order to elucidate a possible reaction route of the synthesized PPGs analogs and their galactosylated precursors with DPPH•, quantum chemical calculations were performed. Theoretical calculations presented here only describe the "fast antioxidant" aspect of the compounds under scrutiny. In this context, two possible mechanisms have been previously proposed for the reaction between radical scavenger molecules (*anti)* and DPPH•, [47], [48a], [48b] the electron transfer (ET) process:

*anti* +*R*• → (*anti*•)^+^ +*R* ^−1^

and the sequential proton loss single electron transfer (SPLET) reaction:

*anti* → (*anti*−H) ^−1^ + *H*+              step 1

(*anti*−H) ^−1^ +*R*• → (*anti*−H)• +*R* ^−1^          step 2

**Figure 4:**
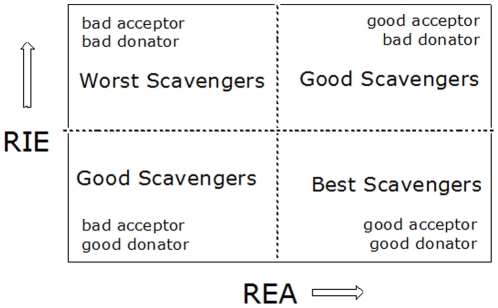
Full electron donator acceptor map (FEDAM). Dashed lines are only included to aid visualization. Adapted from reference 48 b.

For the ET reaction and the second step of the SPLET mechanism, the electron donor capacity of *anti* and (*anti*−H) ^−1^ is important. A simple way to analyze the relative feasibility to donate or accept charge among a set of chemical compounds for full electron transfer processes has been recently reported [48a], [48b]. It is well-known that to characterize the ability to donate or to accept electrons, IEs and EAs are adequate parameters. To analyze the ET process between radical scavengers and free radicals, a full electron donator acceptor map (FEDAM) was defined (Figure 4).

Concerning the construction of the FEDAM, values of IE and EA for fluor (F) and sodium (Na) atoms (obtained at the same level of theory) were used to obtain the corresponding relative values of electron acceptance (REA) and electron donation (RIE) indexes. These indexes are defined according to the following equations:
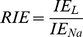
(1)

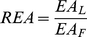
(2)


In these equations, L represents the studied molecule. Using the FEDAM, any substance can be classified in terms of its electron donating and accepting capability (considering F and Na as reference). Therefore, the FEDAM is a useful tool for a qualitative comparison of ET capacity between substances. This mapping process has been used to analyze the intrinsic antioxidant radical scavenging capability of several compounds including carotenoids, which are well-known free radical scavengers.

**Figure 5:**
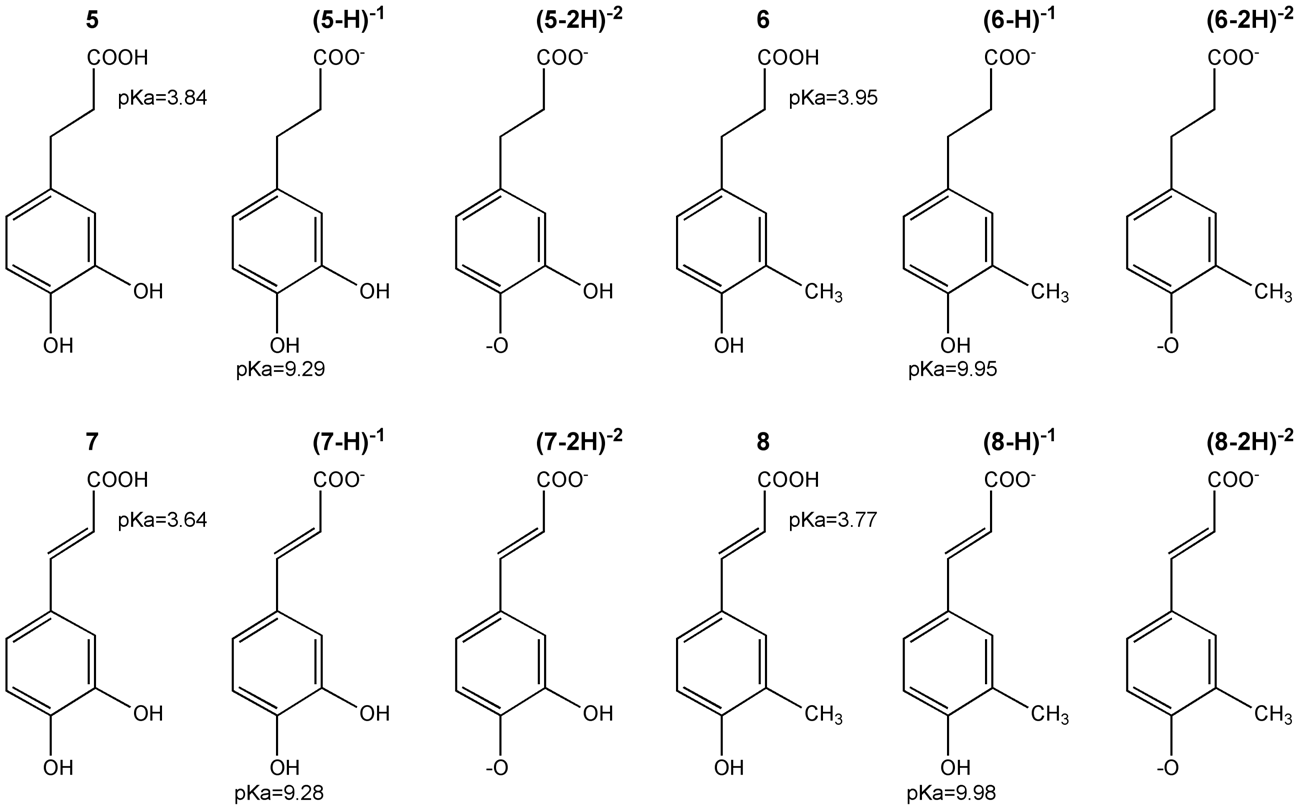
Structural representation of compounds from **Table 1** and their deprotonated species are shown. When known, the experimental pKa values are also included.

To analyze the possible reaction mechanism to scavenge free radicals of the galactosylated PPG precursors as well of the new PPG analogs, compounds **3** and **10** were theoretically studied. Also for comparison, compounds **5, 6, 7** and **8** were analyzed. Optimized structures and theoretical results concerning the optimization are included in the Materials and Methods S1. According with the results given in Table 1, caffeic (**7**) and ferulic acids (**8**) have lower free radical scavenging activities than their corresponding saturated derivates (**5**>**7**>**6**>**8)**. It is well known that the SPLET mechanism explains the reaction of phenols with DPPH•, nevertheless both mechanisms (ET and SPLET) were studied for compounds **3** and **10** and the theoretical results compared to the experimental values. It is important to note that according to their pKa values, compounds **5**, **6**, **7** and **8** are deprotonated in the experimental conditions (pH = 7) as shown in Figure 5. For this reason and to analyze the ET capacity of these compounds, RIE and REA were calculated for **3**, (5-H) ^−1^, (6-H) ^−1^, (7-H) ^−1^, (8-H) ^−1^ and **10**.

**Figure 6:**
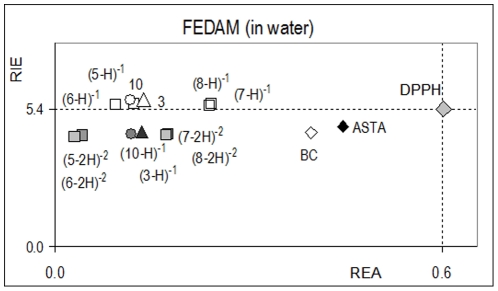
FEDAM in water. Dashed lines are only included to aid visualization. Molecules located below the dashed line are better electron donors when compared to DPPH•. Triangles represent compound 3, circles represent compound 10 and squares represent values of the deprotonated compounds of 5, 6, 7 and 8. β-carotene (BC) and astaxanthin (ASTA) are included for comparison.

To compare with the experimental results, the DPPH• was also calculated. Using RIE and REA, it was possible to locate these molecules in the FEDAM as shown in Figure 5, where previously reported values for β-carotene and astaxanthin are also included for comparison. As shown in Figure 6, among these molecules, DPPH• is the best electron acceptor.

Therefore, the ET mechanism must take place from the radical scavenger molecule to the DPPH free radical. This is only possible if free radical scavenger molecules behave as good electron donors, which is the case of β-carotene and astaxanthin as previously reported [48a], [48b]. In comparison, compounds **3**, (5-H) ^−1^, (6-H) ^−1^, (7-H) ^−1^, (8-H) ^−1^ and **10** are inferior electron donors (RIE values are larger) when compared to β-carotene and astaxanthin (Figure 6). Furthermore, these molecules are not better electron donors when compared to the DPPH•. As a consequence, the ET process from compounds **3**, (5-H) ^−1^, (6-H) ^−1^, (7-H)^1^, (8-H) ^−1^ or **10** to the DPPH• cannot be proposed as the antioxidant reaction mechanism. These radical scavengers cannot lose or donate an electron to deactivate the DPPH•.

**Figure 7:**
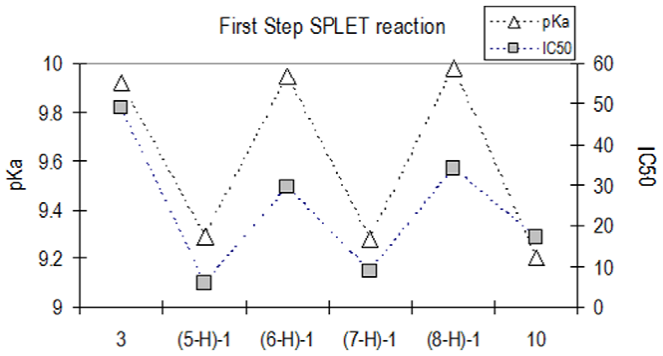
Experimental pKa and IC_50_ values are shown. Dashed lines are only included to aid visualization.

The first step in the SPLET reaction mechanism is the deprotonation of the radical scavenger molecule, and it is possible to correlate the pKa value of the deprotonation process with the SPLET reaction feasibility. Experimental pKa values and results of the scavenging capacity of the analyzed compounds are shown in Figure 7.

A correlation between the pKa values and scavenging capacity of the analyzed compounds was established. To define if this reaction mechanism was able to explain the differences in the experimental scavenging activity, it was necessary to analyze the second step of the SPLET reaction. Therefore, the evaluation of the energetic cost associated to the electron transfer from the antiradical to the free radical (once the deprotonated species are formed) is required. It is important to note that according to the pKa values, only 1% of the compound is deprotonated at physiological pH. This small amount of the deprotonated species may react throughout the ET reaction.

The corresponding deprotonated molecules are (3-H)^−1^, (10-H) ^−1^, (5-2H) ^−2^, (6-2H) ^−2^, (7-2H) ^−2^ and (8-2H) ^−2^ for the compounds that were already deprotonated (Figure 5). All of these molecules are located in the FEDAM (Figure 6), where it is shown that the deprotonated radical scavengers are better electron donors (RIE values are smaller) when compared to the corresponding protonated species. Furthermore, according to the FEDAM, all the deprotonated species have a similar capacity to donate electrons. To corroborate the ET predictions derived from the FEDAM, the energy evolution associated to each ET process between the (*anti-H)* ^−*1*^ or (*anti-2*H) ^−1^ molecules and the DPPH• must be determined. In this context, the corresponding adiabatic Gibbs free energy was computed in water at 298 °K and is reported for the deprotonated molecules (Table 2), while those of the protonated species (**3**,**10** and (anti-H) ^−*1*^ for **5**, **6**, **7** and **8**) are not included because they were all endergonic in water.

**Table 2:** Adiabatic Gibbs free energy in water (kJ/mol) at 298.15 K for reactions between the radical scavengers ((*anti*−H) ^−1^ and (*anti*−2H) ^−2^) and the *DPPH*• are shown.

(*anti*−H) ^−1^ +*DPPH*• → (*anti*−H)• +*DPPH* ^−1^		(*anti*−2H) ^−1^ +*DPPH*• → (*anti*−2H)• +*DPPH* ^−1^			
**(3-H)** ^−**1**^	**(10-H)** ^−**1**^	**(5-2H)** ^−**2**^	**(6-2H)** ^−**2**^	**(7-2H)** ^−**2**^	**(8-2H)** ^−**2**^
−37.5	−55.3	−41.7	−56.1	−36.4	−40.4

As inferred from the values reported in Table 2, the Gibbs free energies of one electron transfer from (*3-*H) ^−1^, (*10-*H) ^−1^, (*5-2*H) ^−2^, (*6-2*H) ^−2^, (*7-2*H) ^−2^ or (8*-2*H) ^−2^ to the DPPH• in water solutions are exergonic. These results correspond with the FEDAM predictions. If this is the case, all deprotonated compounds should have similar scavenging capacities. As reported in Table 1, the experimental scavenging capacity is greater for compounds **5** and **7** when compared to the capacity for compounds **3**, **6**, **8** and **10**. These results are due to the limiting step of deprotonation of the radical scavenger molecule, while the ET rate to the free radical is a fast process. According to their pKa values, compounds **3**, *(6-*H) ^−1^ and (*8-*H) ^−1^ are less reactive than (*5-*H) ^−1^, (7*-*H) ^−1^ and **10**, which is in agreement with the experimental scavenging capacity.

Together, these results suggest that the antioxidant reaction between each of these compounds and the DPPH• occurs through a SPLET mechanism. Similarly, curcumin has recently been reported to react with the DPPH• through the same mechanism [47]. For further experimental designs to synthesize antioxidant molecules, it is important to analyze the pKa values of the reactants to have an idea about the free radical scavenging efficiency towards the DPPH•.

### Conclusions

In this study, an enzymatic process for the synthesis of PPG analogues is reported for the first time. The synthesis of PPGs was studied considering the diversity introduced by the extent of the aliphatic chain in the aglycon, requirement of α−β unsaturation of the carbonyl moiety in the ester residue and number of hydroxyl groups in the PPG aromatic rings as well as their influence on the free radical scavenging activity. From this study, it is concluded that the antiradical activity of precursors **1** and **2** is not affected by the galactosylation of the molecule or by the presence of either ethyl or propyl chains on the aglycon. Based on the results of the DPPH• test, the majority of the enzymatically synthesized PPGs had a free radical scavenging activity that was higher than that of ascorbic acid, which was used as a reference compound. More significantly, their IC_50_ values resulted in similar IC_50_ values than the values reported for natural PPGs. Theoretical evidence indicated that the antioxidant reaction between each of these compounds and the DPPH• proceeds through a SPLET mechanism. The different radical scavenging capacities may be explained by the deprotonation of the radical scavenger molecule as being the limiting step, while the ET rate to the free radical is a fast process. According to this mechanism and considering the pKa values, compounds **3**, *(6-*H) ^−1^ and (*8-*H) ^−1^ are less reactive than compounds (*5-*H) ^−1^, (7*-*H) ^−1^ and **10**, which is in agreement with the experimental scavenging capacity.

In summary, the enzymatic process described in this study is not only an alternative and efficient route to obtain PPGs in cost effective yields, but it is also a procedure to create structural variability in these secondary metabolites. In addition to the antioxidant characteristics of the PPGs synthesized in this study, there is a large amount of evidence supporting the fact that PPGs bear potent structurally dependent nutraceutical activities.

## Supporting Information

Figure S1 (TIF)

Materials and Methods S1 (DOC)

## References

[1] Wildman, REC (2006). Handbook of nutraceuticals and functional foods..

[2] Kurkin VA (2003). Phenylpropanoids from medicinal plants: distribution, classification, structural analysis, and biological activity.. Chem Nat Comp.

[3] Pan J, Yuan C, Lin C, Jia Z, Zheng R (2003). Pharmacological activities and mechanism of natural phenyl propanoid glycosides.. Pharmazie.

[4] Laporta O, Perez-Fons L, Balan K, Paper D, Cartagena V, Micol V (2004). Bifunctional antioxidative oligosaccharides with antiinflammatory activity for joint health.. Agro Food Ind Hi-Tech.

[5] Zhang SQ, Li ZJ, Wang AB, Cai MS, Feng R (1997). Total synthesis of the phenylpropanoid glycoside, grayanoside A.. Carbohydr Res.

[6] Zhang SQ, Li ZJ, Wang AB, Cai MS, Feng R (1998). Synthesis of a phenylpropanoid glycoside, Osmanthuside B6.. Carbohydr Res.

[7] Moon JK, Shibamoto T (2009). Antioxidant assays for plant and food components.. J Agric Food Chem.

[8] Shi Y, Wang W, Kang J, Shi Y, Jia Z, Wang Y (1999). Reaction of hydroxyl radical with phenylpropanoid glycoside and its derivatives by pulse radiolysis.. Sci China Ser C-Life Sci.

[9] Robin JR, Rolland Y, inventors (2004). Use of Verbascoside as a stimulant agent for the production of thermal shock proteins by the cells of the skin.. French patent FR WO/2004/069218.

[10] Balan K, Paper D, inventors (2006). Use of an Extract of *Aloysia*/*Verbena*/*Lippia* triphylla/Citriodora for the treatment of chronic and/or inflammatory diseases.. United States patent US 2006/0280820A1.

[11] Li Q, Li SC, Li H, Cai MS, Li ZJ (2005). Total synthesis of syringalide B, a phenylpropanoid glycoside.. Carbohydr Res.

[12] Li Z, Zhang S, Wang A, Cai M (1998). Studies on glycosides X X VI: Total synthesis of the phenylpropanoid glycoside, Eutigoside A.. Acta Chim Sin.

[13] Kawada T, Asano R, Hayashida S, Sakuno T (1999). Total synthesis of the phenylpropanoid glycoside, acteoside.. J Org Chem.

[14] Duynstee HI, de Koning MC, Ovaa H, van Der Marel GA, Van Boom JH (1999). Synthesis of verbascoside: A dihydroxyphenylethyl glycoside with diverse bioactivity.. Eur J Org Chem.

[15] Kawada T, Asano R, Makino K, Sakuno T (2000). Synthesis of conandroside: A dihydroxyphenylethyl glycoside from *Conandron ramaidioides*.. Eur J Org Chem.

[16] Kawada T, Asano R, Makino K, Sakuno T (2002). Synthesis of isoacteoside, a dihydroxyphenylethyl glycoside.. J Wood Sci.

[17] Kawada T, Yoneda Y, Asano R, Kan-no I, Schmid W (2006). Synthesis of plantamajoside, a bioactive dihydroxyphenylethyl glycoside from *Plantago major*.. L Holzforschung.

[18] Zhou FY, She J, Wang YG (2006). Synthesis of a benzyl-protected analog of arenarioside, a trisaccharide phenylpropanoid glycoside.. Carbohydr Res.

[19] Bojarová P, Kren V (2009). Glycosidases: a key to tailored carbohydrates.. Trends Biotechnol.

[20] Gotor-Fernández V, Brieva R, Gotor V (2006). Lipases: Useful biocatalysts for the preparation of pharmaceuticals.. J Mol Catal B-Enzym.

[21] Bridiau N, Taboubi S, Marzouki N, Legoy MD, Maugard T (2006). β-Galactosidase catalyzed selective galactosylation of aromatic compounds.. Biotechnol Prog.

[22] Otto RT, Scheib H, Bornscheuer UT, Pleiss J, Syldatk C, Schmid RD (2000). Substrate specificity of lipase B from *Candida antarctica* in the synthesis of arylaliphatic glycolipids.. J Mol Catal B-Enzym.

[23] Cassani J, Luna H, Navarro A, Castillo E (2007). Comparative esterification of phenylpropanoids versus hydrophenylpropanoids acids catalyzed by lipase in organic solvent media.. Electron J Biotechnol.

[24] Stevenson DE, Wibisono R, Jensen DJ, Stanley RA, Cooney JM (2006). Direct acylation of flavonoid glycosides with phenolic acids catalysed by *Candida antarctica* lipase B (Novozym 435®).. Enzyme Microb Technol.

[25] Kennedy JF, Kumar H, Panesar PS, Marwaha SS, Goyal R, Parmar A (2006). Enzyme-catalyzed regioselective synthesis of sugar esters and related compounds.. J Chem Technol Biotechnol.

[26] Lee GS, Widjaja A, Ju YH (2006). Enzymatic synthesis of cinnamic acid derivatives.. Biotechnol Lett.

[27] Stamatis H, Sereti V, Kolisis FN (2001). Enzymatic synthesis of hydrophilic and hydrophobic derivatives of natural phenolic acids in organic media.. J Mol Catal B-Enzym.

[28] Nenadis N, Boyle S, Bakalbassis EG, Tsimidou M (2003). An experimental approach to structure-activity relationships of caffeic and dihydrocaffeic acids and related monophenols.. J Am Oil Chem Soc.

[29] Shimoji Y, Tamura Y, Nakamura Y, Nanda K, Nishidai S, Nishikawa Y (2002). Isolation and identification of DPPH radical scavenging compounds in Kurosu (Japanese unpolished rice vinegar).. J Agric Food Chem.

[30] Lee S, Lee CH, Kim E, Jung SH, Lee HK (2007). Hydroxylated hydrocinnamides as hypocholesterolemic Agents.. Bull Korean Chem Soc.

[31] Moon JK, Shibamoto T (2010). Antioxidant activities, total phenolics and flavonoids content in two varieties of Malaysia Young Ginger (*Zingiber officinale* Roscoe).. Molecules.

[32] Molyneux P (2004). The use of the stable free radical diphenylpicrylhydrazyl (DPPH) for estimating antioxidant activity.. Songklanakarin J Sci Technol.

[33] Magalhães LM, Segundo MA, Reis S, Lima JL (2008). Methodological aspects about in vitro evaluation of antioxidant properties.. Anal Chim Acta.

[34] Yamaguchi T, Takamura H, Matoba T, Terao J (1998). HPLC method for evaluation of the free radical-scavenging activity of foods by using 1,1-diphenyl-2-picrylhydrazyl.. Biosci Biotechnol Biochem.

[35] a) Becke AD (1998) Density-functional exchange-energy approximation with correct asymptotic behavior. Phys Rev A 38: 3098–3100. b) Lee C, Yang W, Parr RG (1998) Development of the Colle-Salvetti correlation-energy formula into a functional of the electron density. Phys Rev B 37: 785–789.

[36] a) Krishnan R, Binkley JS, Seeger R, Pople JA (1980) Self-consistent molecular orbital methods. XX. A bases set for correlated wave functions. J Chem Phys 72: 650–654. b) McLean AD, Chandler GS (1980) Contracted Gaussian basis sets for molecular calculations. I. Second row atoms, Z = 11-18. J Chem Phys 72: 5639–5648. c) Clark T, Chandrasekhar J, Spitznagel GW, Schleyer PVR (1980) Efficient diffuse function-augmented basis sets for anions calculations. III. The 3-21+G basis set for first-row elements, Li-F. J Comput Chem 4: 294–301.

[37] a) Cancès MT, Mennucci B, Tomasi J (1997) A new integral equation formalism for the polarizable continuum model: Theoretical background and applications to isotropic and anisotropic dielectrics. J Chem Phys 107: 3032–3041. b) Mennucci B, Tomasi J (1997) Continuum solvation models: A new approach to the problem of solute’s charge distribution and cavity boundaries. J Chem Phys 106: 5151–5158. c) Tomasi J, Mennucci B, Cancès E (1997) The IEF version of the PCM salvation method: an overview of a new method addressed to study molecular solutes at the QMab initio level. J Mol Struct 464: 211–226.

[38] Frisch MJ, Trucks GW, Schlegel HB, Scuseria GE, Robb MA, Cheeseman JR (2004). Gaussian, Inc..

[39] Stevenson DE, Stanley RA, Furneaux RH (1993). Optimization of alkyl β-D-galactopyranoside synthesis from lactose using commercially available β-galactosidases.. Biotechnol Bioeng.

[40] Sabally K, Karboune S, St-Louis R, Kermasha S (2007). Lipase-catalyzed synthesis of phenolic lipids from fish liver oil and dihydrocaffeic acid.. Biocatal Biotransform.

[41] Castillo E, Pezzotti F, Navarro A, López-Munguía A (2003). Lipase-catalyzed síntesis of xylitol monoesters: solvent engineering approach.. J Biotechnol.

[42] Castillo E, Torres-Gavilán A, Severiano P, Navarro A, López-Munguía A (2007). Lipase-catalyzed synthesis of pungent capsaicin analogues.. Food Chem.

[43] Silva FA, Borges F, Guimarães C, Lima JL, Matos C, Reis S (2000). Phenolic acids and derivatives: studies on the relationship among structure, radical scavenging activity, and physicochemical parameters.. J Agric Food Chem.

[44] Aligiannis N, Mitaku S, Tsitsa-Tsardis E, Harvala C, Tsaknis I, Lalas S (2003). Methanolic extract of *Verbascum macrurum* as a source of natural preservatives against oxidative rancidity.. J Agric Food Chem.

[45] Ersöz T, Alipieva KI, Yalcin FN, Akbay P, Handjieva N, Dönmez AA (2003). Physocalycoside, a new phenylethanoid glycoside from*Phlomis physocalyx* Hub.-Mor.. Z Naturforsch C.

[46] Delazar A, Sabzevari A, Mojarrab M, Nazemiyeh H, Esnaashari S, Nahar L (2008). Free-radical-scavenging principles from *Phlomis caucasica*.. J Nat Med.

[47] Galano A, Alvarez-Diduk R, Ramirez-Silva MT, Alarcón-Ángeles G, Rojas-Hernández A (2009). Role of the reacting free radicals on the antioxidant mechanism of Curcumin.. Chem Phys.

[48] a) Martínez A, Vargas R, Galano A (2009) What is important to prevent oxidative stress? A theoretical study on electron transfer reactions between carotenoids and free radicals. J Phys Chem B 113: 12113–12120. b) single walled carbon nanotubes with different structures through electron transfer reactions. J Phys Chem C 114: 8184–8191. 10.1021/jp903958h 19642662

