# Supplementary material for: Phenylpropanoid Glycoside Analogues: Enzymatic Synthesis, Antioxidant Activity and Theoretical Study of Their Free Radical Scavenger Mechanism
Source: PLoS One. 2011 Jun 3;6(6):e20115. doi: 10.1371/journal.pone.0020115 (PMC3108595; doi:10.1371/journal.pone.0020115)
Supplement: Materials and Methods S1 — (DOC) [file pone.0020115.s002.doc]

**Supporting Information**

**Phenylpropanoid glycoside analogues:**

**enzymatic synthesis, antioxidant activity and theoretical study of their free radical scavenger mechanism**

Agustín López Munguía^1^, Yanet Hernández-Romero^1^, José Pedraza-Chaverri^2^, Alfonso Miranda-Molina^1^, Ignacio Regla^4^, Ana Martínez^3*^, and Edmundo Castillo^1*^

^1^Departamento Ingeniería Celular y Biocatálisis, Instituto de Biotecnología, Universidad Nacional Autónoma de México, Cuernavaca, Morelos, México.

^2^Departamento de Biología, Facultad de Química, Universidad Nacional Autónoma de México, Cd. Universitaria, México DF, México.

^3^Departamento de Materia Condensada y Criogenia, Instituto de Investigaciones en Materiales, Universidad Nacional Autónoma de México, Ciudad Universitaria, México DF, México.

^4^Facultad de Estudios Superiores Zaragoza, Universidad Nacional Autónoma de México, Batalla del 5 de mayo y Fuerte de Loreto, México, DF

Spectra Data Details

The ^1^H-NMR spectrum of **13** indicated the presence of a set of signals of an aromatic ABX system [δ 6.66 (1H, d, *J* = 1.6 Hz, H-2’’), δ 6.69 (1H, d, *J* = 8.4 Hz, H-5’’), δ 6.51 (1H, dd, *J* = 2.0 Hz, *J* = 8 Hz, H-6’’)] and two CH_2_ protons [δ 2.80 (2H, t, *J* = 7.2 Hz, H-7’’), δ 2.54 (2H, t, *J* = 7.2 Hz, H-8’’)] in the spectrum suggested the dihydrocaffeoyl substituent. Other ABX aromatic protons [δ 6.90 (1H, d, *J* = 2.0 Hz, H-2), δ 6.70 (1H, d, *J* = 8.4 Hz, H-5), δ 6.67 (1H, dd, *J* = 1.8 Hz, *J* = 8.2 Hz, H-6)], a benzylic CH_2_ group at δ 2.75 (2H, t, *J* = 7.6 Hz, H-7), and two nonequivalent H-atoms of a CH_2_-O group [δ 3.98 (1H, t, *J* = 7.2 Hz, H-8a), δ 3.68 (1H, dt, *J*= 1.6 and 7.2 Hz, H-8b)] and a signal at δ 3.79 (3H, s, -OCH_3_) were assigned to the 4-hydroxy-3-methoxyphenyl)ethoxy moiety (aglycone). The dihydrocaffeoyl group was positionated at C-6’ of the galactose ring on the basis of the strong deshielding of the signals of proton spectra [δ 4.26 (1H, m, H-6’a), δ 4.22 (1H, dd, *J* = 5.6 Hz, *J* = 10.8 Hz, H-6’b)], which was further confirmed by three-bond correlation of CH_2_ (6’) to C=O (C-9’’) group at δ 172.91 in the HMBC spectrum. The ^1^H-NMR spectrum showed one anomeric proton at δ 4.25 (1H, d, *J* = 7.6 Hz, H-1’) which was confirmed by the corresponding anomeric carbon at δ 104.42 in the ^13^C-NMR, and by HMBC connectivities with H-3’ (δ 3.50) and with signal of δ 3.98 and δ 3.68 corresponding to H-atoms of CH_2_-O group in the position 8. Compound **13** was obtained as a white amorphous powder with a molecular weight of 494 g/mol consistent with the molecular formula C_24_H_30_O_11_ and confirmed by HRFAB-MS.

(4-hydroxy-3-methoxyphenyl)methyl-β-D-galactopyranoside (**3**). ^1^H NMR (400 MHz, CDCl_3_): δ = 6.97 (1H, d, *J* = 1.6 Hz, H-2), 6.74 (1H, dd, *J* = 1.6 Hz, *J* = 8.0 Hz, H-6), 6.70 (1H, d, *J* = 7.6 Hz, H-5), 5.2 (1H, d, *J* = 11.2 Hz, H-7a), 4.9 (1H, d, *J* = 11.2 Hz, H-7b), 4.12 (1H, d, *J* = 7.6, H-1’), 3.63 (1H, m, H-2’), 3.60 (1H, t, *J* = 3.6 Hz, H-4’), 3.74 (3H, s, -OCH_3_), 3.33 (1H, *J* = 5.8 Hz, *J* = 10.6 Hz, H-3’), 3.25 (1H, m, H-5’), 3.6-3.44 (2H, m, H-6’). ^13^C NMR (100 MHz): δ = 147.39 (C-3), 145.85 (C-4), 128.88 (C-1), 120.67 (C-6), 115.05 (C-5), 112.32 (C-2), 102.33 (C-1’), 75.35(C-4’), 73.56 (C-5’), 70.78 (C-3’), 69.63(C-7), 68.37 (C-2’), 60.72 (C-6’), 55.712 (-OCH_3_). HRFABMS (positive mode), calcd for C_14_H_20_O_8_: 316.1158, found: 316.1167.

2-(4-hydroxy-3-methoxyphenyl)ethyl-β-D-galactopyranoside (**4**). ^1^H NMR (400 MHz, CD_3_OD): δ = 6.85 (1H, s, H-2), 6.70 (1H, d, *J* = 8 Hz, H-6), 6.66 (1H, d, *J* = 8.4 Hz, H-5), 4.26 (1H, d, *J* = 7.2 Hz, H-1’), 4.05 (dd, *J* = 7.4 Hz, *J* = 16.6 Hz, H-8a), 3.83 (3H, s, -OCH_3_), 3.76 (1H, dd, *J* = 6.8 Hz, *J* = 11.2 Hz, H-8b), 3.74 (2H, m, H-6’), 3.50 (1H, m, H-2’), 3.49 (1H, m, H-5’), 3.47 (1H, m, H-4’), 3.46 (1H, dd, *J* = 3.0 Hz, *J* = 10.2 Hz, H-3’), 2.84 (2H, t, *J* = 7.2, H-7). ^13^C NMR (100 MHz): δ = 148.96 (C-1), 146.02 (C-3), 131.83 (C-4), 122.55 (C-6), 116.23 (C-5) 113.98 (C-2), 105.09 (C-1’), 76.77 (C-5’), 75.18 (C-3’), 72.72 (C-2’), 72.05 (C-8), 70.47 (C-4’), 62.66 (C-6’), 56.58 (-OCH_3_), 36.91 (C-7). HRFABMS (positive mode), calcd for C_15_H_22_O_8_: 330.1315, found: 330.1351.

(4-hydroxy-3-methoxyphenyl)methyl-6-*O*-dihydroferuloyl-β-D-galactopyranoside (**9**). ^1^H NMR (400 MHz, pyridine-*d*_5_): δ = 7.28 (1H, d, *J* = 1.2 Hz, H-2), 7.21 (1H, d, *J* = 9.2 Hz, H-5), 7.20 (1H, dd, *J* = 1.2 Hz, *J* = 8.8 Hz, H-5’’), 7.13 (1H, dd, *J* = 1.2 Hz, *J* = 8.0 Hz, H-6), 6.99 (1H, d, *J* = 1.6, H-2’’), 6.90 (1H, dd, *J* = 1.2 Hz, *J* = 8.0 Hz, H-6’’), 5.2 (1H, d, *J* = 11.2 Hz, H-7a), 4.99 (1H, dd, *J* = 7.2 Hz, *J* = 11.2 Hz, H-6’a), 4.41 (1H, d, *J* = 3.2, H-4’), 4.93 (1H, d, *J* = 8.0 Hz, H-1’), 4.88 (1H, d, *J* = 11.2 Hz, H-7b), 4.85 (1H, dd, *J* = 5.6 Hz, *J* = 11.6 Hz, H-6’b), 4.54 (1H, dd, *J* = 8.0 Hz, *J* = 9.2 Hz, H-2’), 4.19 (1H, dd, *J* = 3.2 Hz, *J* = 9.6 Hz, H-3’), 4.15 (1H, m, H-5’), 3.76 (3H, s, -OCH_3_), 3.65 (3H, s, -OCH_3_), 2.8 (1H, m, H-7’’). δ = ^13^C NMR (100 MHz): 173.47 (C-8’’), 149.12 (C-3’’), 149.06 (C-3), 148.35 (C-4’’), 147.16 (C-4), 132.56 (C-1’’), 130.05 (C-1), 122.59 (C-6’’), 121.89 (C-6), 117.09 (C-5’’), 116.71 (C-5), 113.56 (C-2’’), 113.30 (C-2), 104.64 (C-1’), 75.57 (C-3’), 74.31 (C-2’), 72.87 (C-4’), 71.85 (C-7), 70.70 (C-5’), 65.42 (C-6’), 56.48 (-OCH_3_), 56.31 (-OCH_3_), 37.41 (C-7’’). HRFABMS (positive mode), calcd for C_24_H_30_O_11_Na: 517.4899, found: 517.1659.

(4-hydroxy-3-methoxyphenyl)methyl-6-*O*-dihydrocaffeoyl-β-D-galactopyranoside (**10**). ^1^H NMR (400 MHz, CD_3_OD): δ = 7.01 (1H, d, *J* = 1.6 Hz, H-2), 6.80 (1H, dd, *J* = 1.8 Hz, *J* = 8.2 Hz, H-6), 6.73 (1H, d, *J* = 8.0 Hz, H-5), 6.66 (1H, d, *J* = 8.0 Hz, H-5’’), 6.64 (1H, d, *J* = 2.0 Hz, H-2’’), 6.52 (1H, dd, *J* = 2.2 Hz, 7.8 Hz, H-6’’), 4.74 (1H, d, *J* = 11.6 Hz, H-7b), 4.53 (1H, d, *J* = 11.2 Hz, H-7a), 4.31 (1H, dd, *J* = 7.6 Hz, *J* = 11.4 Hz, H-6’a), 4.25 (1H, d, *J* = 7.6 Hz, H-1’), 4.22 (1H, dd, *J* = 4.0 Hz, *J* = 10.8 Hz, H-6’b), 3.83 (3H, s, -OCH_3_), 3.73 (1H, d, *J* = 3.2, H-4’), 3.59 (1H, m, H-5’), 3.55 (1H, dd, *J* = 7.8 Hz, *J* = 9.6 Hz, H-2’), 3.43 (1H, dd, *J* = 3.6 Hz, *J* = 9.6 Hz, H-3’), 2.80 (1H, t, *J* = 8.0 Hz, H-7’’), 2.60 (1H, t, *J* = 8.0 Hz, H-8’’). ^13^C NMR (100 MHz): δ = 174.81 (C-9’’), 149.08 (C-4), 147.47 (C-3), 146.38 (C-3’’), 144.85 (C-4’’), 133.61 (C-1’’), 130.50 (C-1), 122.60 (C-6), 120.75 (C-6’’), 116.67 (C-2’’), 116.59 (C-5’’), 115.94 (C-5), 113.51 (C-2), 103.48 (C-1’), 74.88 (C-3’), 74.16 (C-5’), 72.55 (C-2’), 71.99 (C-7), 70.43 (C-4’), 64.83 (C-6’), 56.55 (-OCH_3_), 37.42 (C-8’’), 31.68 (C-7’’). HRFABMS (positive mode), calcd for C_23_H_29_O_11_Na: 503.1530, found: 503.1451.

2-(4-hydroxy-3-methoxyphenyl)ethyl-6-*O*-dihydroferuloyl-β-D-galactopyranoside (**12**). ^1^H NMR (400 MHz, CDCl_3_): δ = 7.19 (1H, d, *J* = 7.6 Hz, H-5), 7.17 (1H, d, *J* = 8.0 Hz, H-5’’), 7.11 (1H, d, *J* = 1.6 Hz, H-2), 6.95 (1H, d, *J* = 1.6 Hz, H-2’’), 6.87 (1H, dd, *J* = 1.6 Hz, *J* = 8.0 Hz, H-6’’), 6.85 (1H, dd, *J* = 2.0 Hz, *J* = 8.0 Hz, H-6), 4.94 (1H, dd, *J* = 7.2 Hz, *J* = 11.2 Hz, H-6’a), 4.84 (1H, d, *J* = 7.2 Hz, H-1’), 4.83 (1H, t, *J* = 2.4 Hz, H-4’), 4.80 (1H, dd, *J* = 5.2 Hz, *J* = 11.2, Hz, H-6’b), 4.50 (1H, dd, *J* = 7.8 Hz, *J* = 9.4 Hz, H-2’), 4.19 (1H, dd, *J* = 3.4 Hz, *J* = 9.4 Hz, H-3’), 4.15 (1H, m, H-5’), 4.14 (2H, t, *J* = 6.0 Hz, *J* = 6.4 Hz, H-8), 4.01 (2H, t, *J* = 7.2 Hz, H-7), 3.76 (3H, s, -OCH_3_), 3.74 (3H, s, -OCH_3_), 3.05 (2H, t, *J* = 7.2 Hz, *J* = 7.6 Hz, H-8’’), 2.77 (2H, t, *J* = 7.2 Hz, *J* = 7.6 Hz, H-7’’). ^13^C NMR (100 MHz): δ = 173.4 (C-9’’), 149.13 (C-3’’), 149.05 (C-3), 147.18 (C-4’’), 147.10 (C-4), 132.57 (C-1’’), 130.74 (C-1), 122.60 (C-6’’), 121.90 (C-6), 117.11 (C-5’’), 116.95 (C-5), 114.06 (C-2’’), 113.39 (C-2), 105.75 (C-1’), 75.62 (C-3’), 74.30 (C-2’), 72.84 (C-4’), 71.84 (C-7), 70.67 (C-5’), 65.33 (C-6’), 56.54 (-OCH_3_), 50.31 (-OCH_3_), 37.37 (C-8’’), 31.72 (C-7’’). HRFABMS (positive mode), calcd for C_25_H_32_O_11_: 508.1945, found: 508.1934.

2-(4-hydroxy-3-methoxyphenyl)ethyl-6-*O*-dihydrocaffeoyl-β-D-galactopyranoside (**13**). ^1^H NMR (400 MHz, acetone-*d*_6_): δ = 6.90 (1H, d, *J* = 2.0 Hz, H-2), 6.70 (1H, d, *J* = 8.4 Hz, H-5), 6.69 (1H, d, *J* = 8.4 Hz, H-5’’), 6.67 (1H, dd, *J* = 1.8 Hz, *J* = 8.2 Hz, H-6), 6.66 (1H, d, *J* = 1.6 Hz, H-2’’), 6.51 (1H, dd, *J* = 2.0 Hz, *J* = 8.0 Hz, H-6’’), 4.26 (1H, m, H-6’a), 4.25 (1H, d, *J* = 7.6 Hz, H-1’), 4.22 (1H, dd, *J* = 5.6 Hz, *J* = 10.8 Hz, H-6’b), 3.98 (1H, t, *J* = 7.2 Hz, H-8a), 3.95 (1H, dd, *J* = 7.6 Hz, *J* = 9.6 Hz, H-2’), 3.79 (3H, s, -OCH_3_), 3.68 (1H, dt, *J* = 7.2 Hz, *J* = 1.6 Hz, H-8b), 3.67 (1H, m, H-5’), 3.52 (1H, t, *J* = 2.4 Hz, H-4’), 3.50 (1H, dd, *J* = 3.8 Hz, *J* = 10.2 Hz, H-3’), 2.80 (2H, t, *J* = 7.2 Hz, H-7’’), 2.75 (2H, t, *J* = 7.6 Hz, H-7), 2.54 (2H, t, *J* = 7.2 Hz, *J* = 8.0 Hz, H-8’’). ^13^C NMR (100 MHz): δ = 172.91 (C-9’’), 149.70 (C-3), 148.13 (C-3’’), 145.7 (C-4’’), 144.3 (C-4), 133.10 (C-1’’), 131.06 (C-1), 122.05 (C-6’’), 120.22 (C-6), 116.17 (C-5’’), 116.02 (C-5), 115.52 (C-2’’), 113.36 (C-2), 104.42 (C-1’), 74.37 (C-5’), 73.30 (C-3’), 72.13 (C-4’), 71.32 (C-2’), 69.61 (C-8), 64.47 (C-6’), 64.13 (C-8’’), 56.20 (-OCH_3_), 36.84 (C-7), 36.54 (7’’). HRFABMS (positive mode), calcd for C_24_H_30_O_11_Na: 517.1686, found: 517.1735.


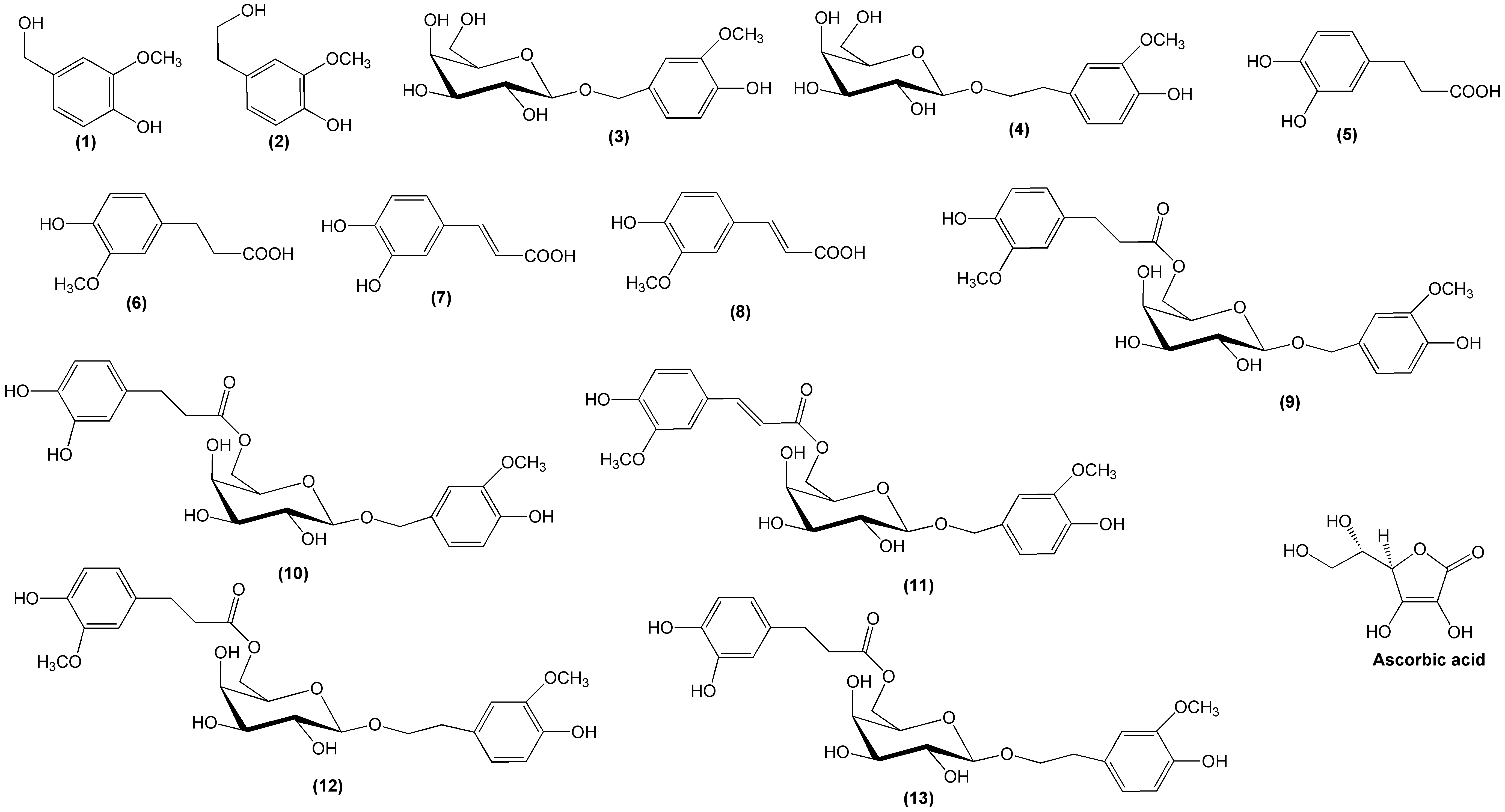


Figure S1. Structure of compounds analyzed by the DPPH• assay

**SPECTRAL DATA OF COMPOUNDS**

(4-hydroxy-3-methoxyphenyl)methyl-β-D-galactopyranoside (**3**)


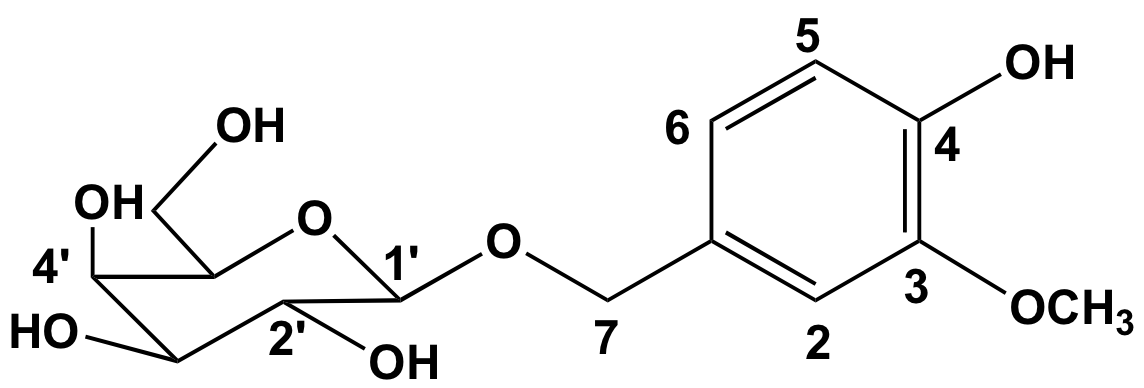


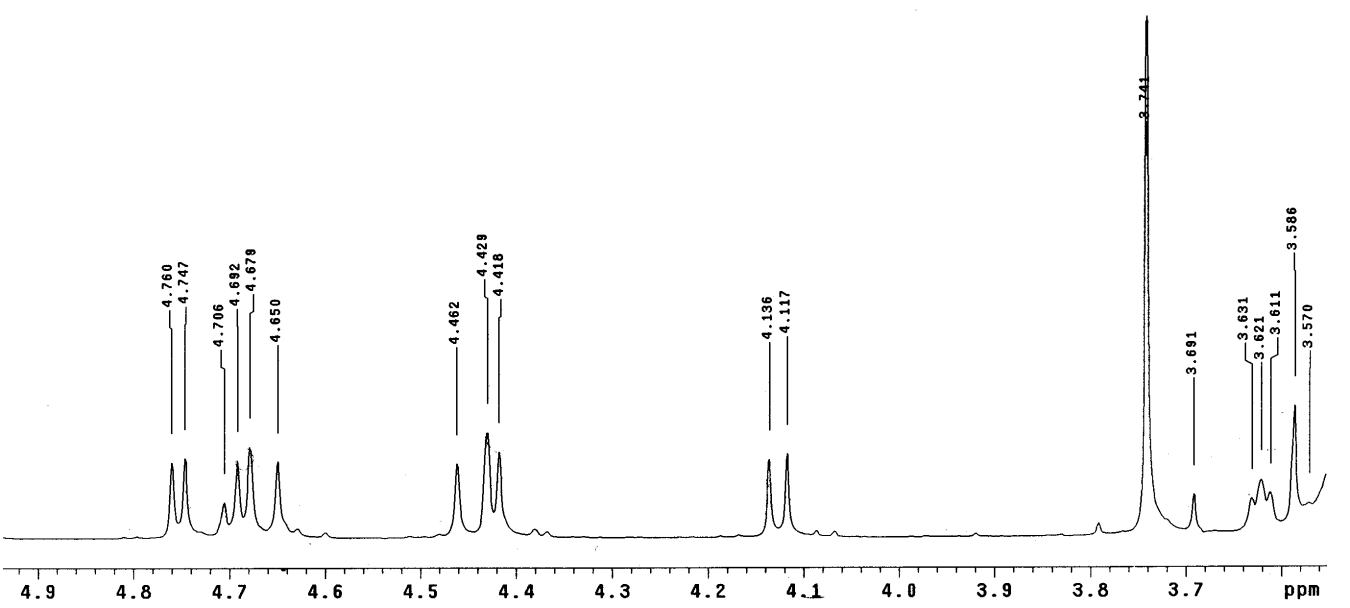


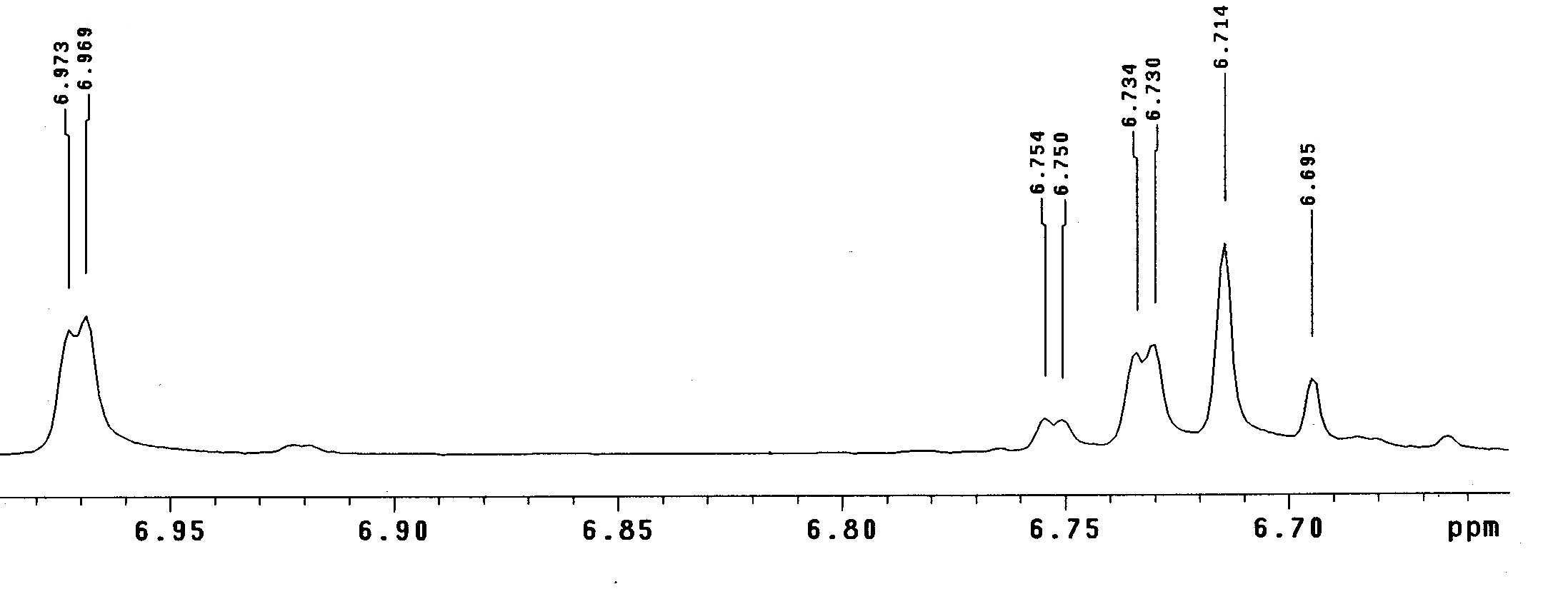

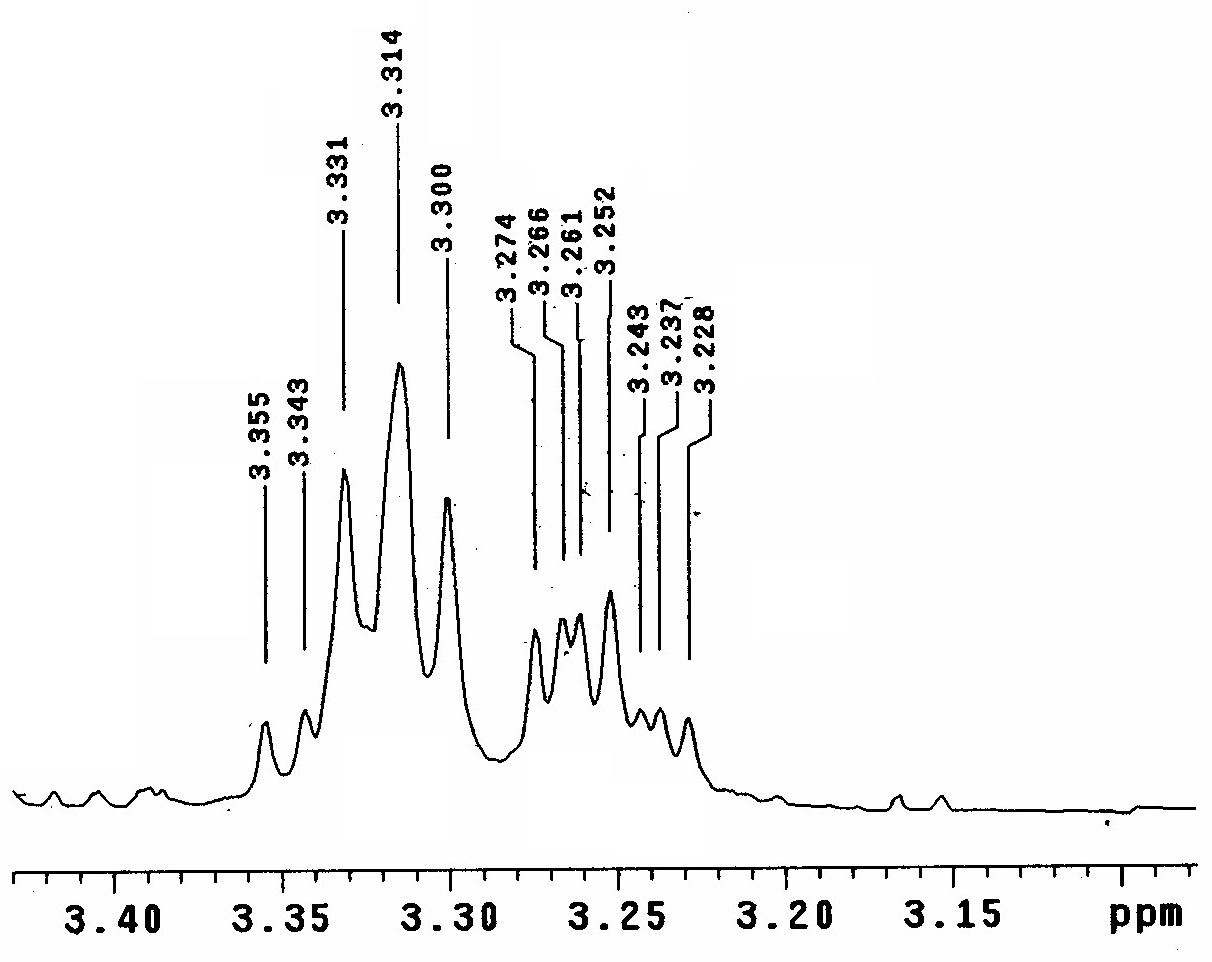

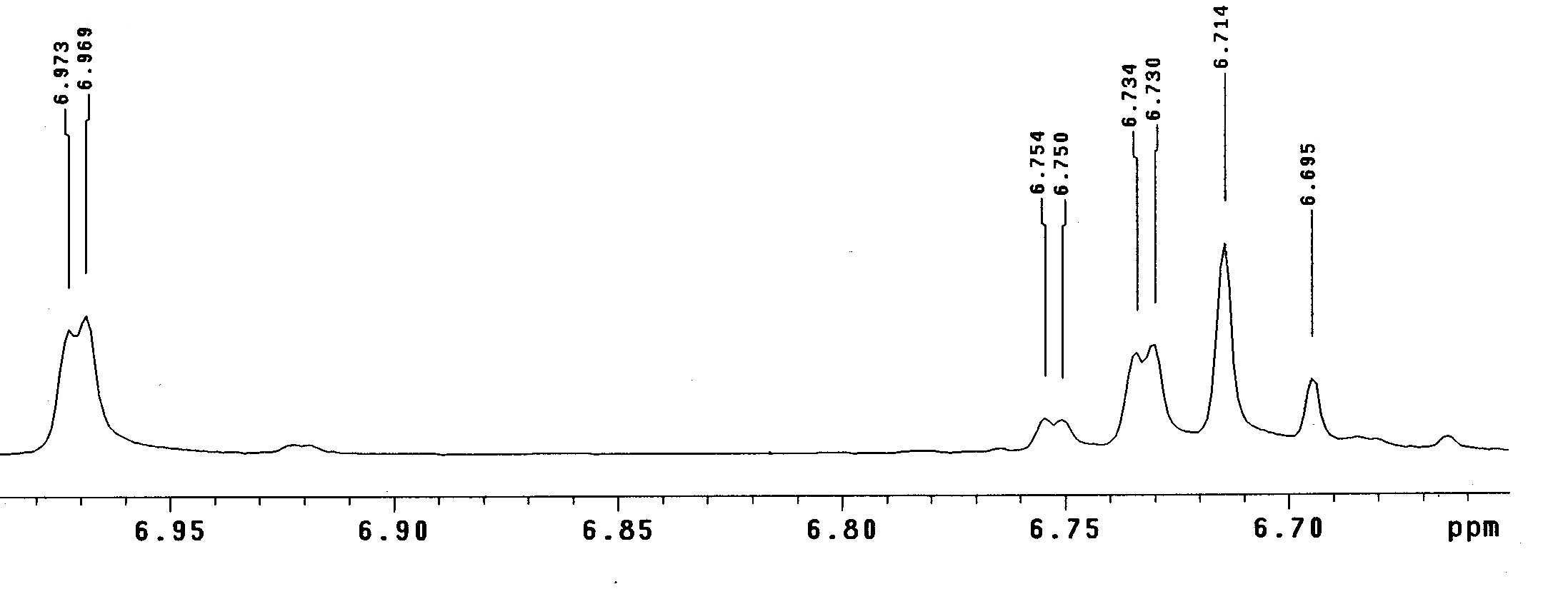


^1^H NMR spectrum of (4-hydroxy-3-methoxyphenyl)methyl-β-D-galactopyranoside (**3**)


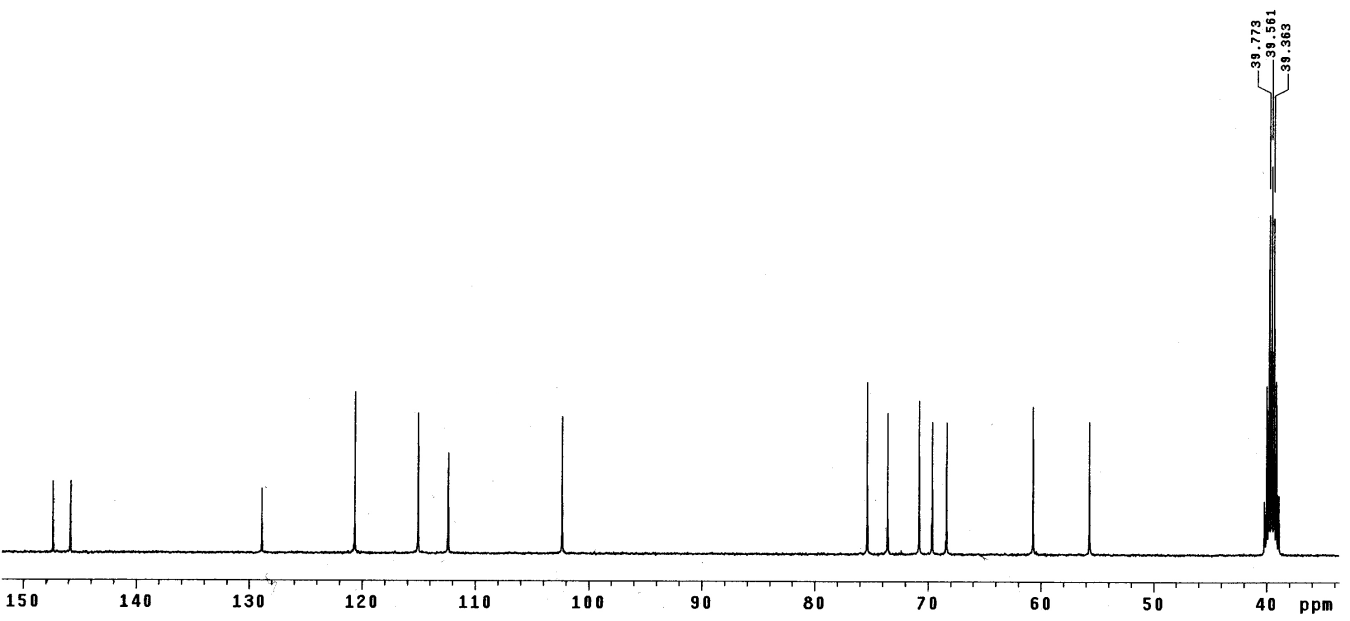


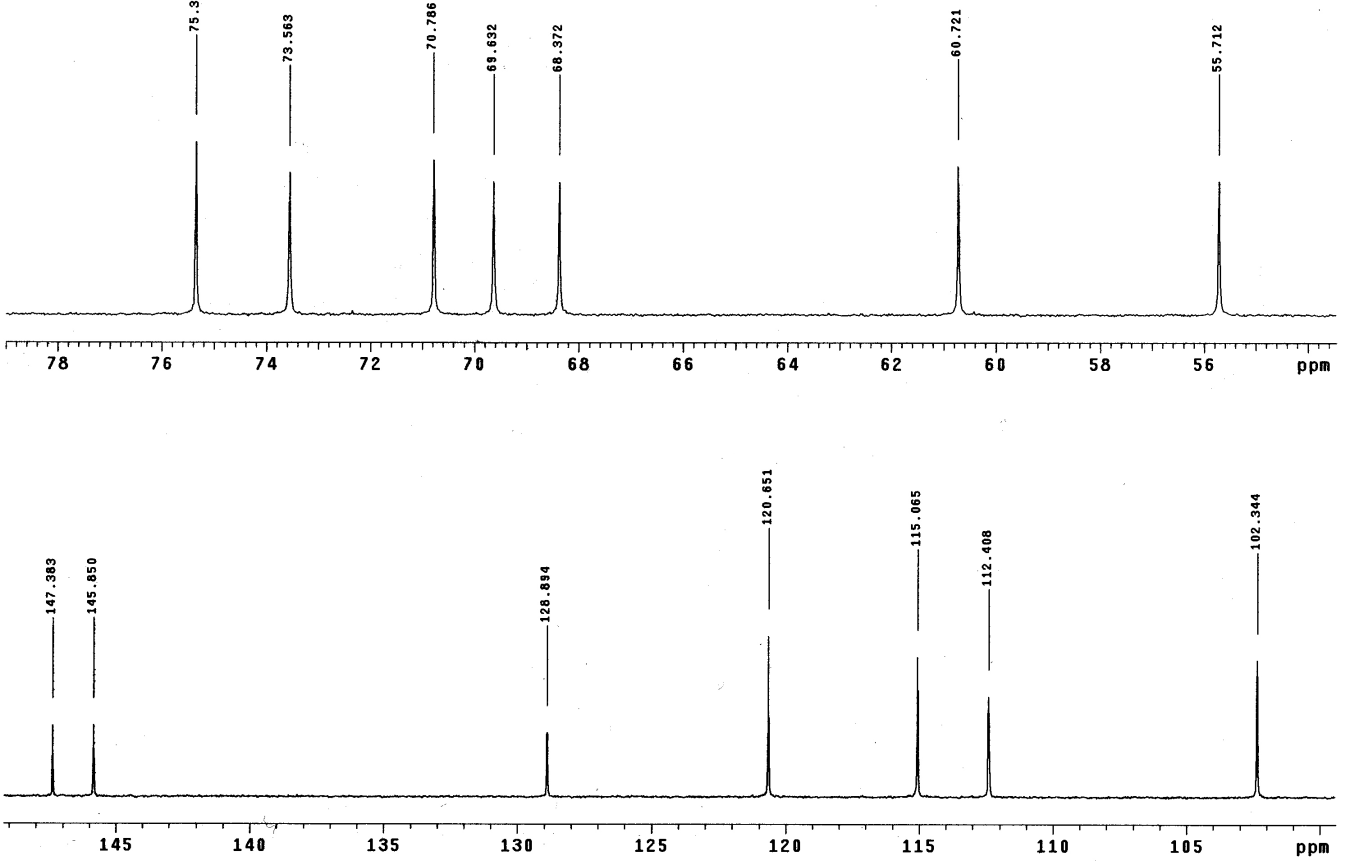


^13^C NMR spectrum of (4-hydroxy-3-methoxyphenyl)methyl-β-D-galactopyranoside (**3**)


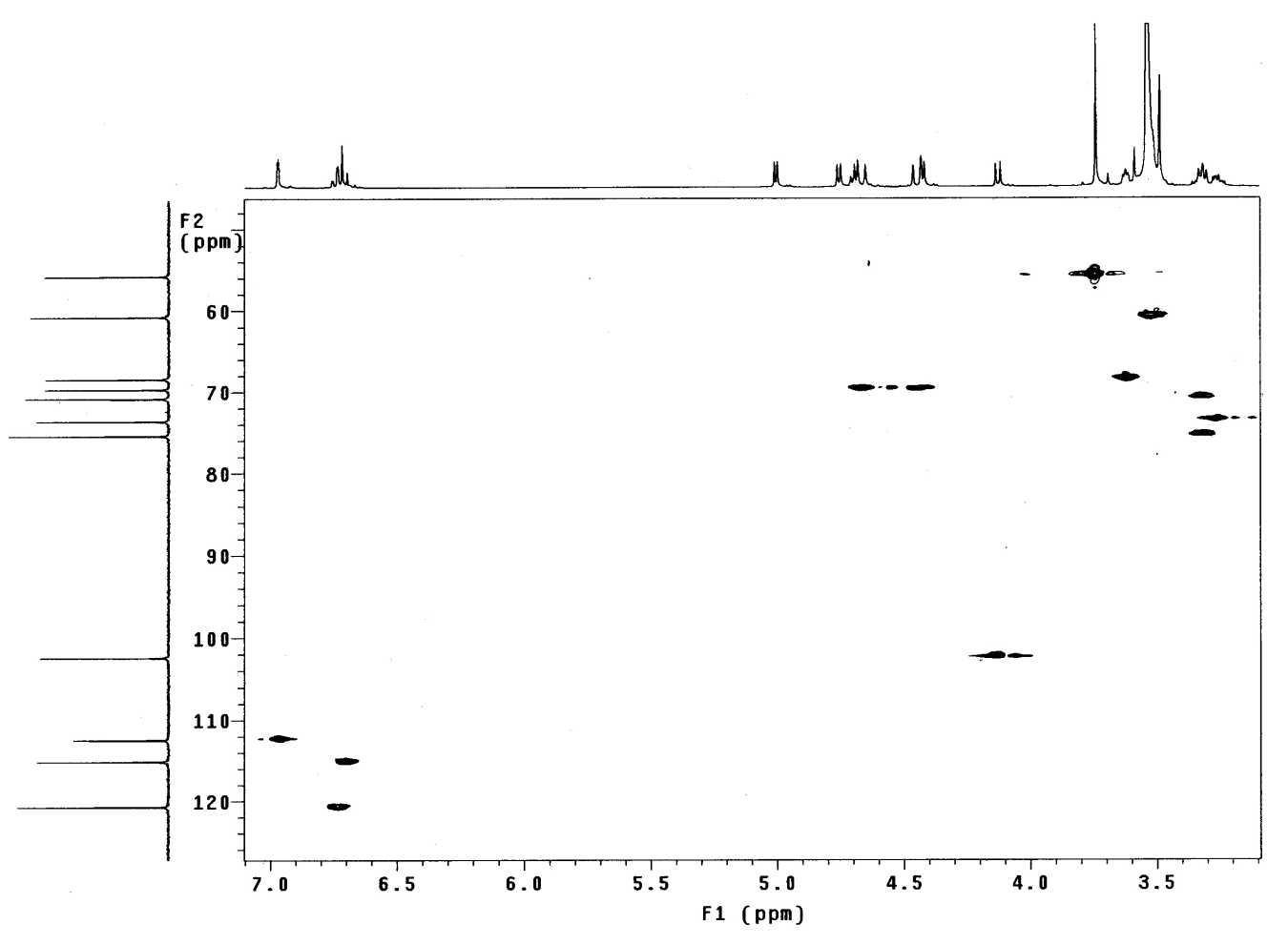


HETCOR spectrum of (4-hydroxy-3-methoxyphenyl)methyl-β-D-galactopyranoside (**3**)

Mass spectrum of (4-hydroxy-3-methoxyphenyl)methyl-β-D-galactopyranoside (**3**)

2-(4-hydroxy-3-methoxyphenyl)ethyl-β-D-galactopyranoside (**4**)


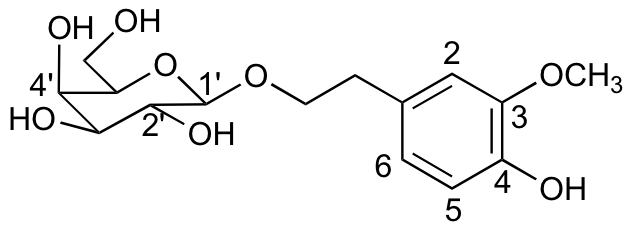


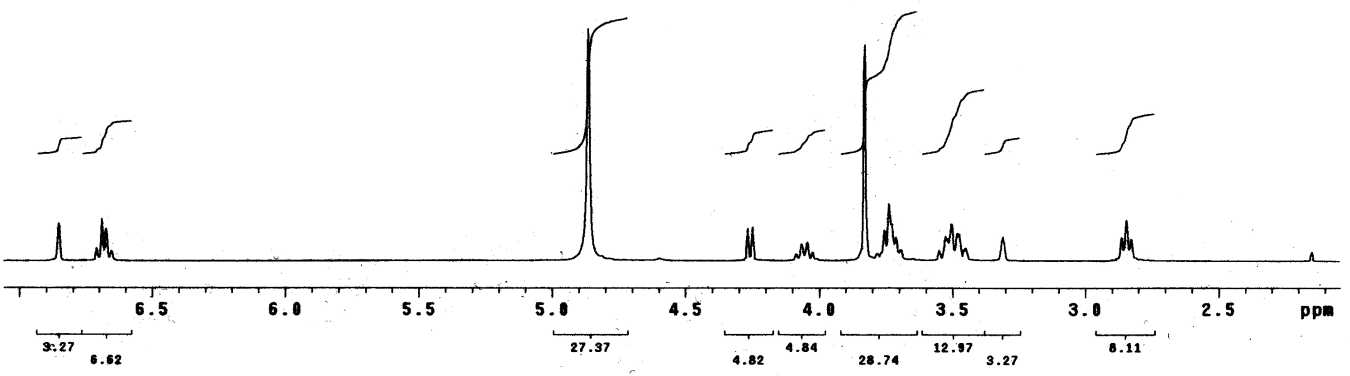


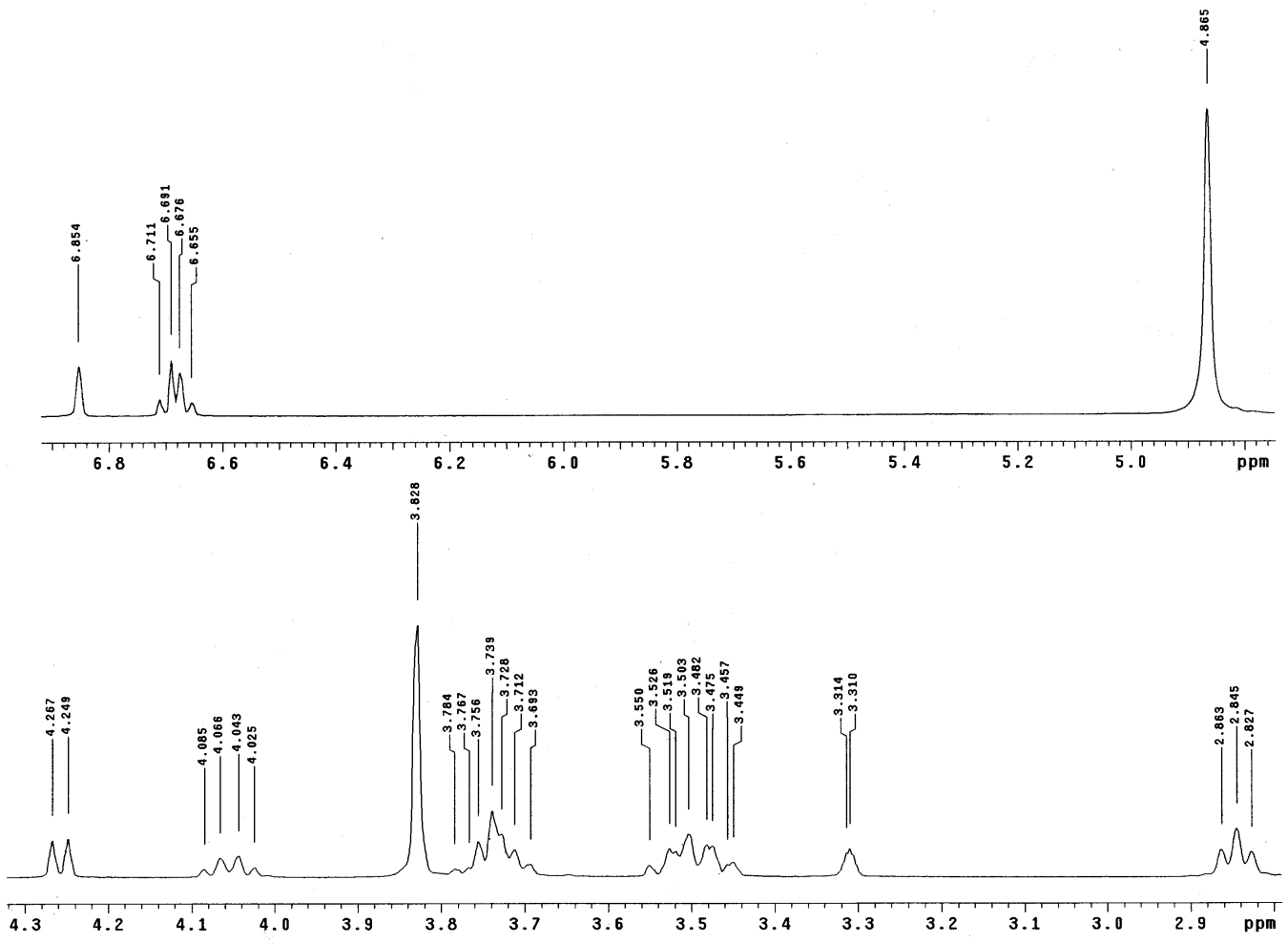


^1^H NMR spectrum of 2-(4-hydroxy-3-methoxyphenyl)ethyl-β-D-galactopyranoside (**4**)


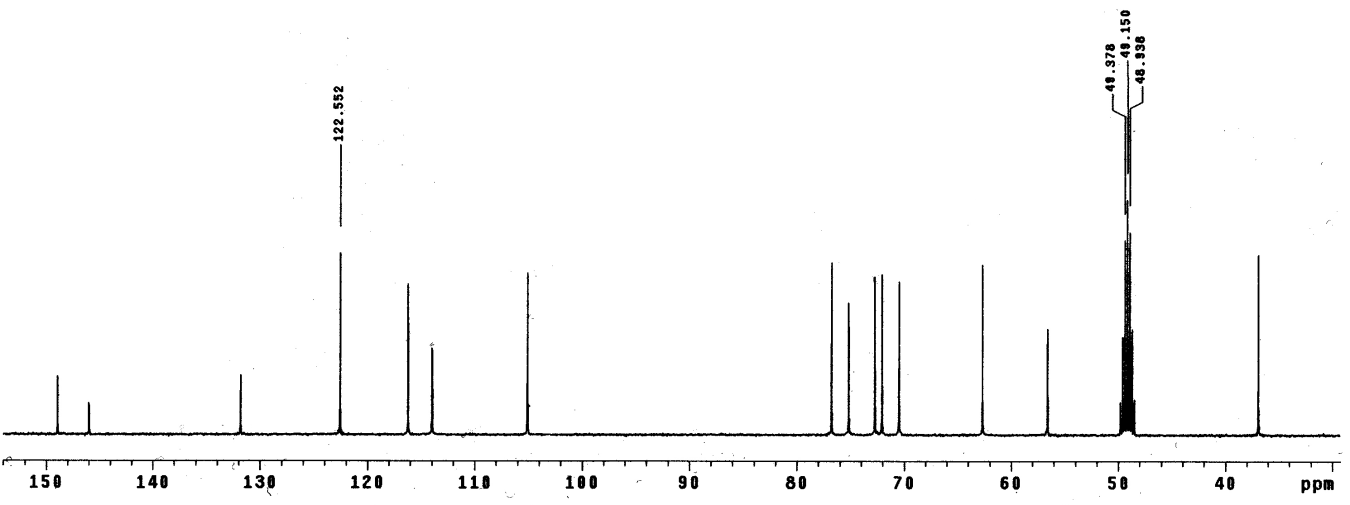


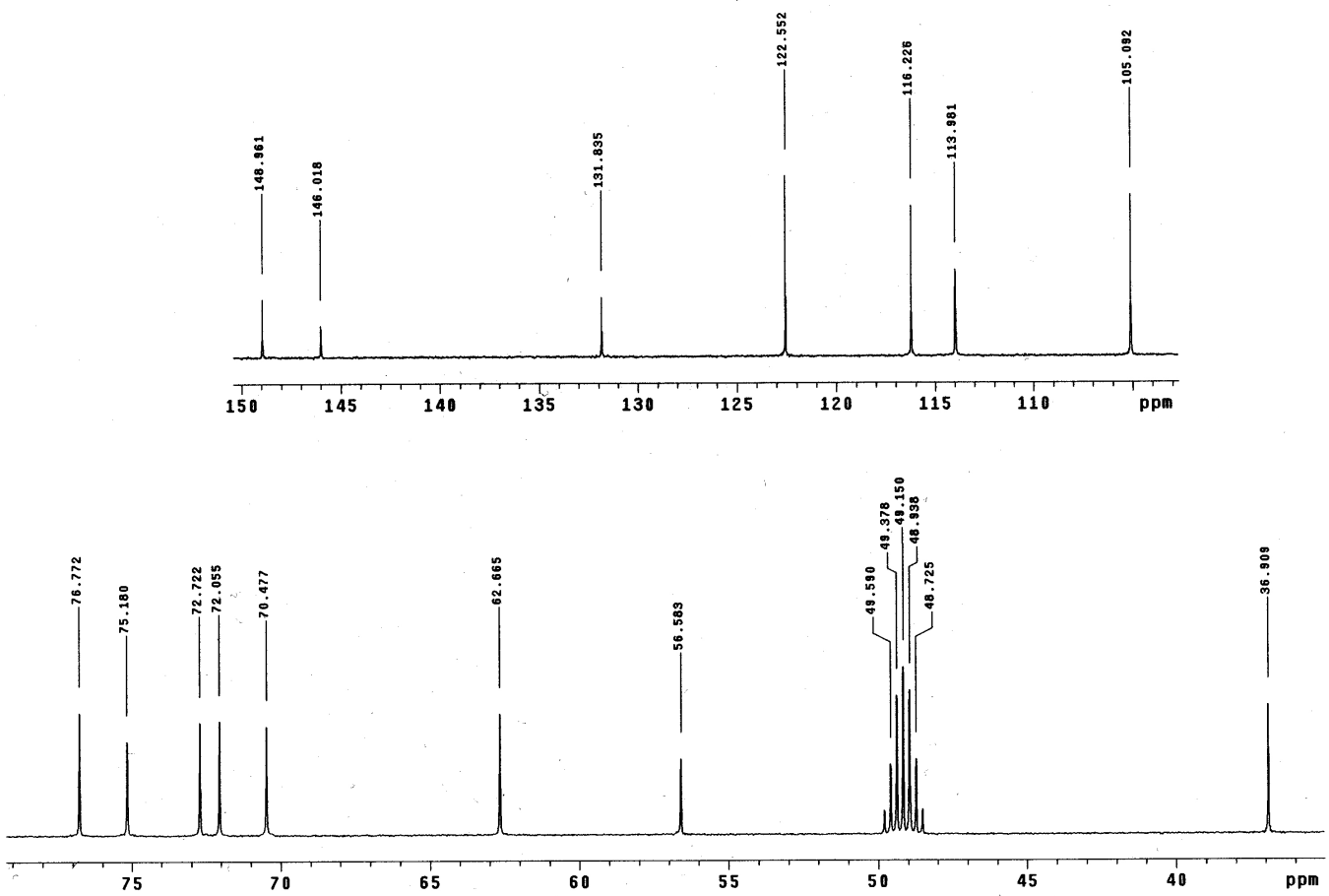


^13^C NMR spectrum of 2-(4-hydroxy-3-methoxyphenyl)ethyl-β-D-galactopyranoside (**4**)


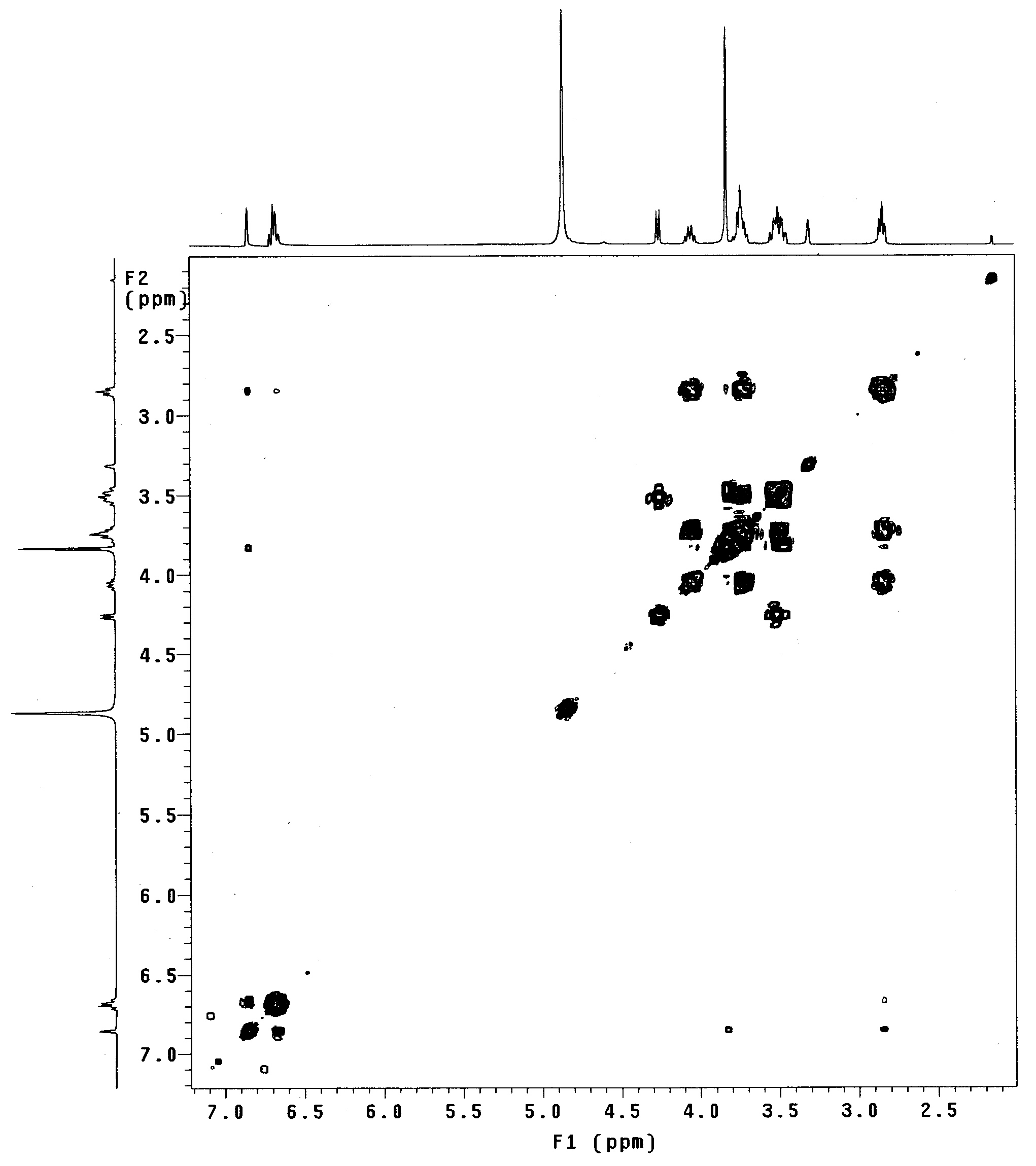


COSY spectrum of 2-(4-hydroxy-3-methoxyphenyl)ethyl-β-D-galactopyranoside (**4**)


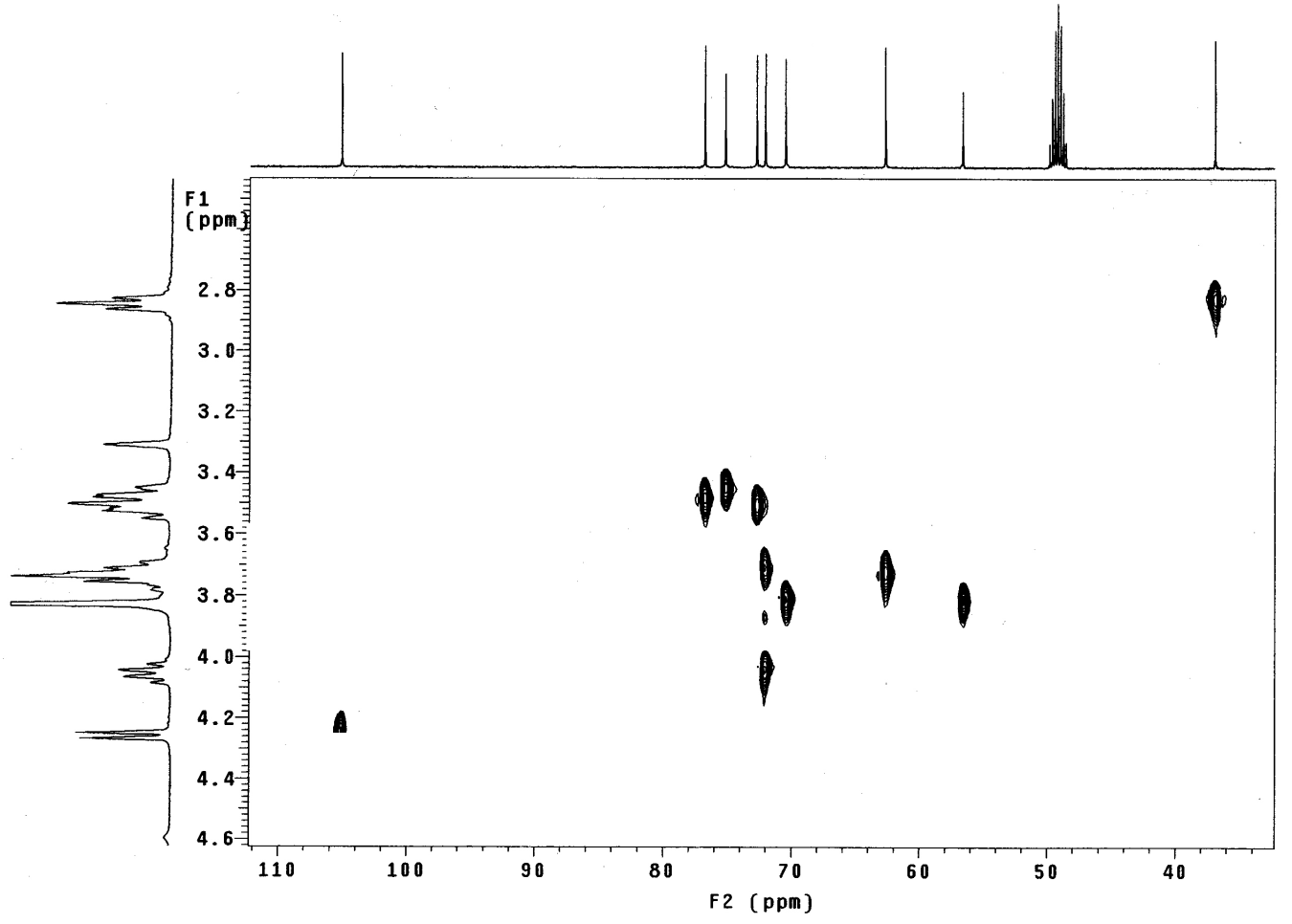


HETCOR spectrum of 2-(4-hydroxy-3-methoxyphenyl)ethyl-β-D-galactopyranoside (**4**)

Mass spectrum of 2-(4-hydroxy-3-methoxyphenyl)ethyl-β-D-galactopyranoside (**4**)

(4-hydroxy-3-methoxyphenyl)methyl-6-*O*-dihydroferuloyl-β-D-galactopyranoside (**9**)


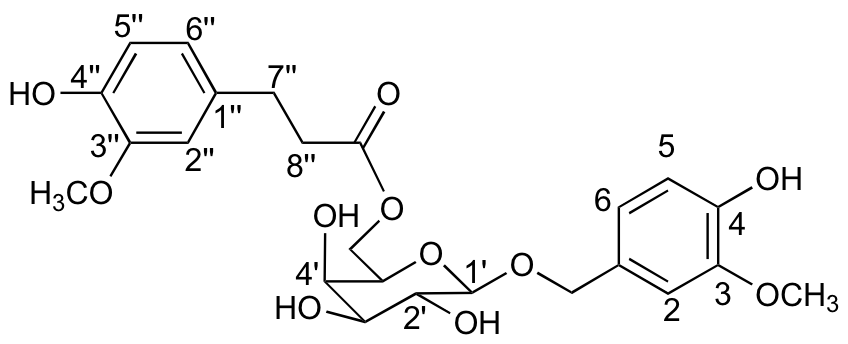


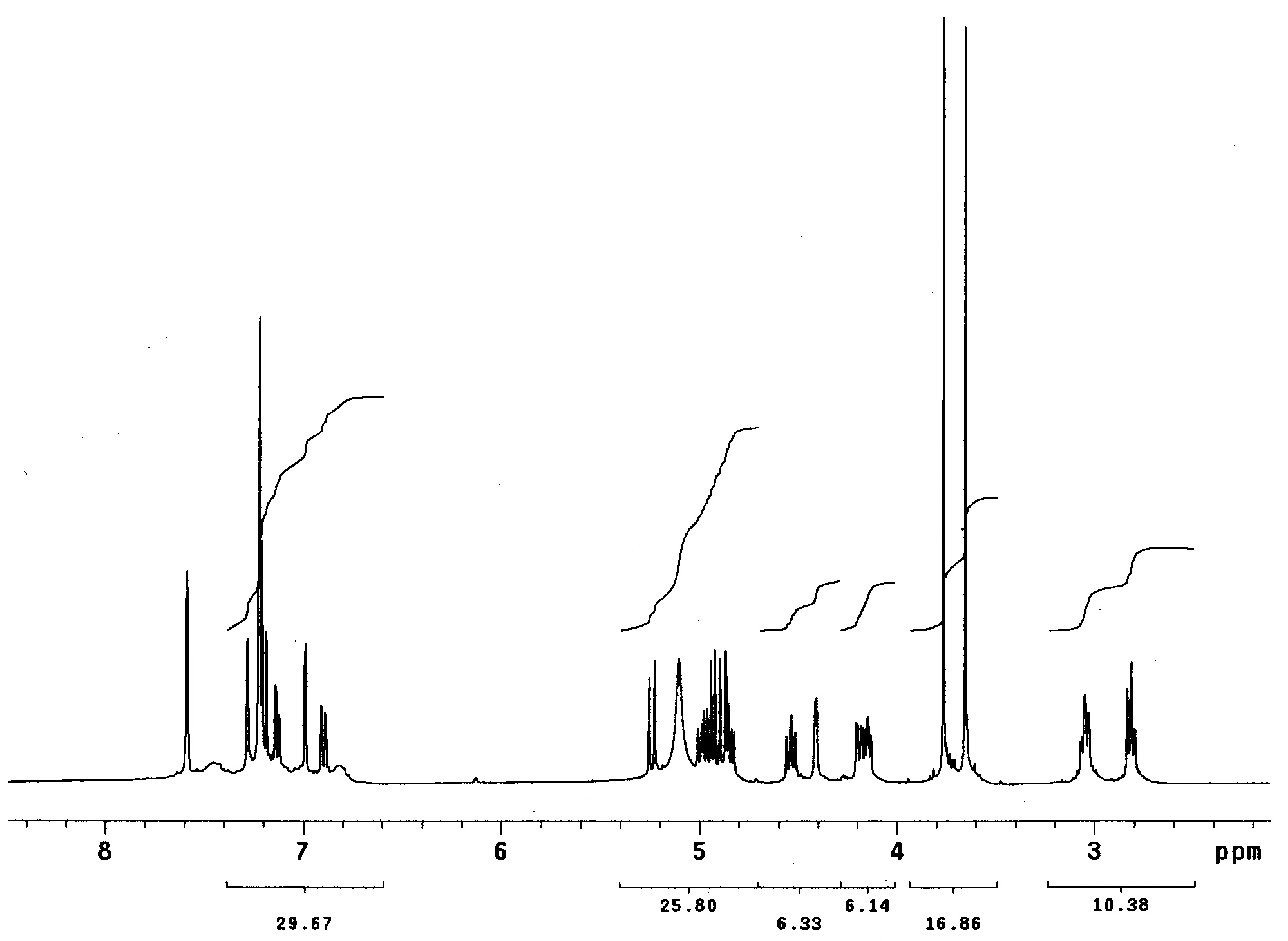


^1^H NMR spectrum of (4-hydroxy-3-methoxyphenyl)methyl-6-*O*-dihydroferuloyl-β-D-galactopyranoside (**9**)


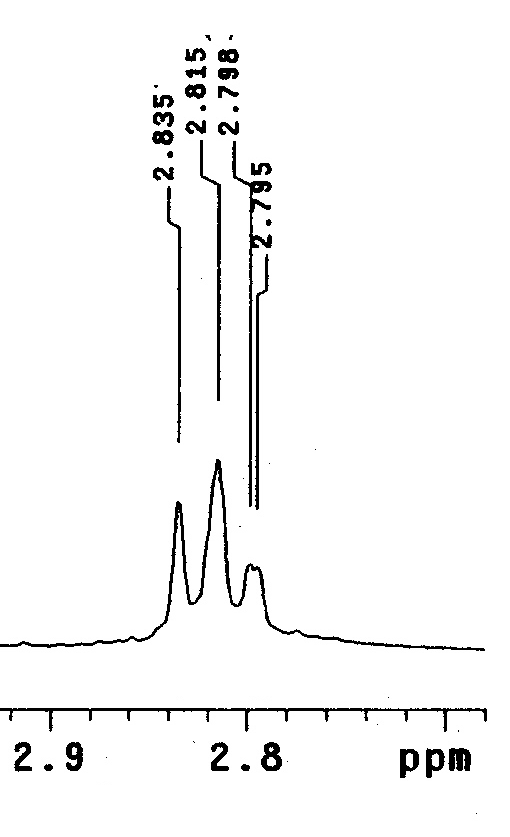

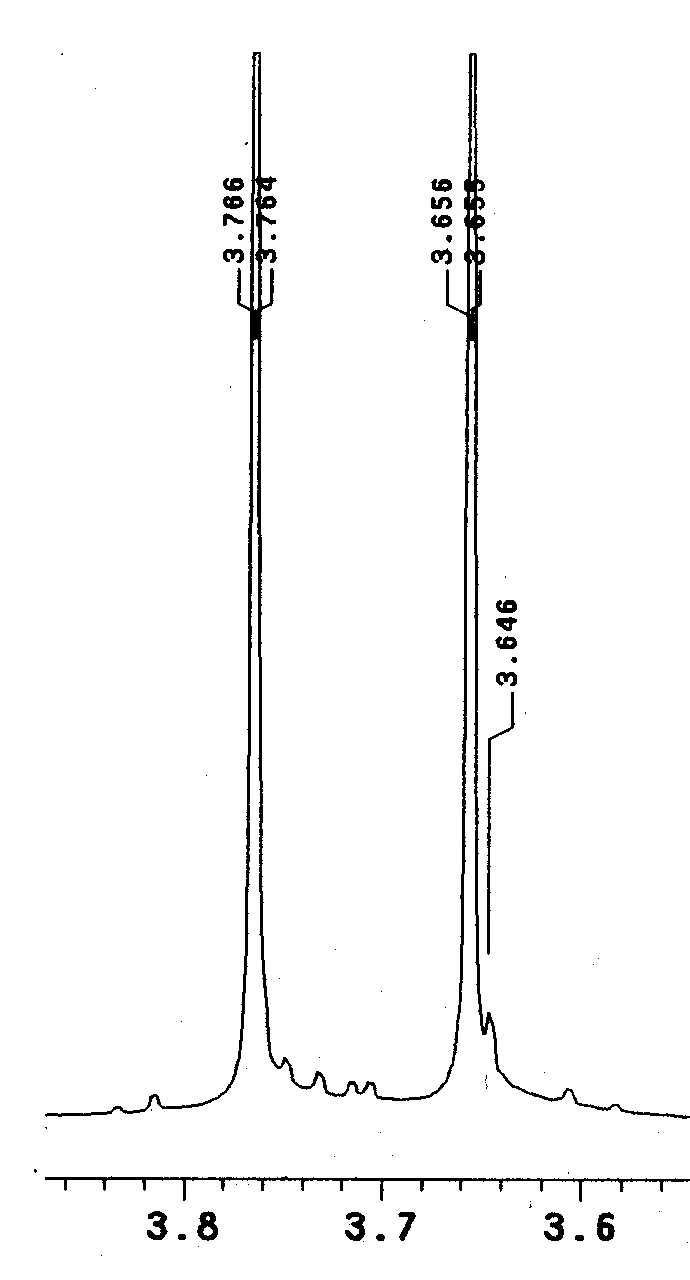


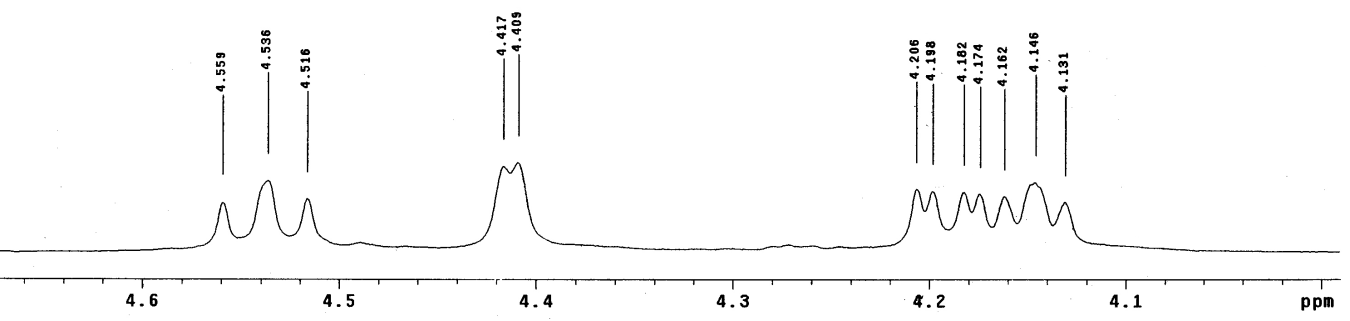


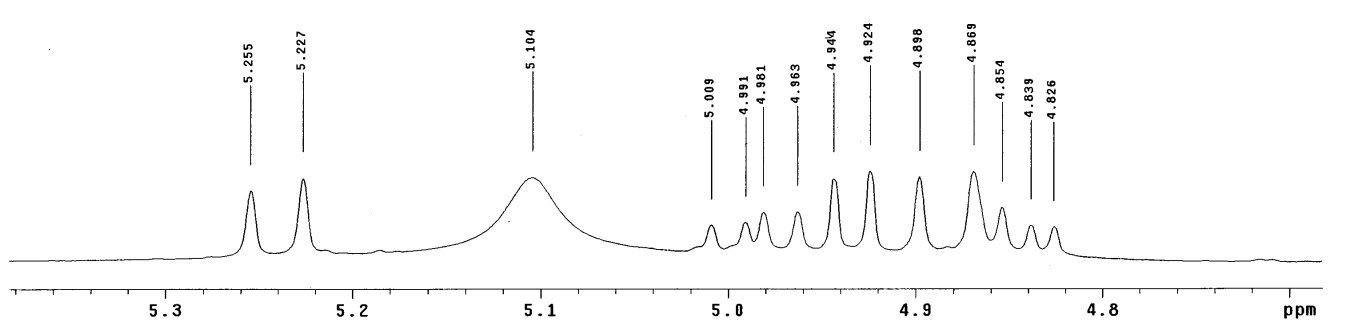


^1^H NMR spectrum of (4-hydroxy-3-methoxyphenyl)methyl-6-*O*-dihydroferuloyl-β-D-galactopyranoside (**9**)


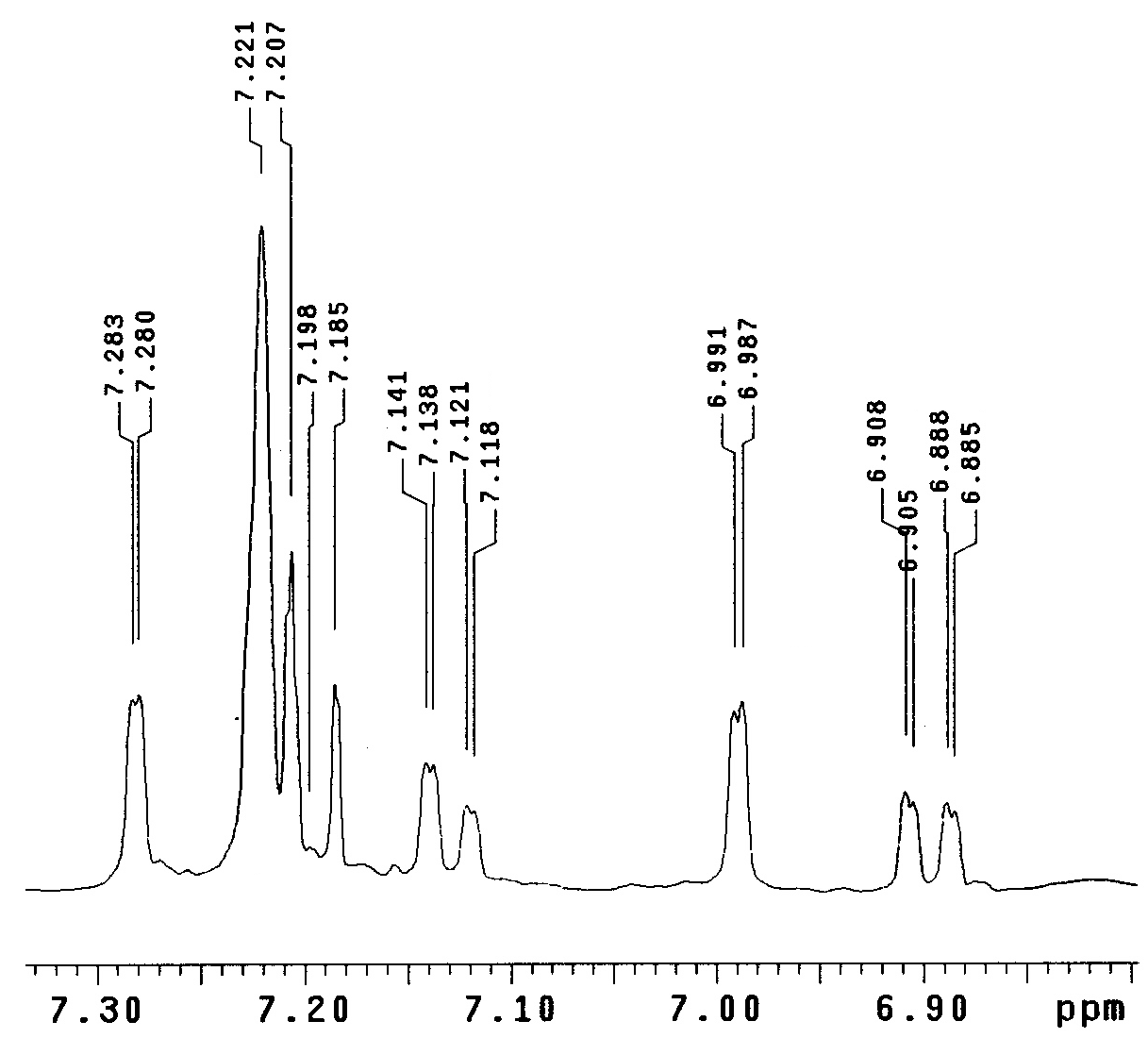


^1^H NMR spectrum of (4-hydroxy-3-methoxyphenyl)methyl-6-*O*-dihydroferuloyl-β-D-galactopyranoside (**9**)


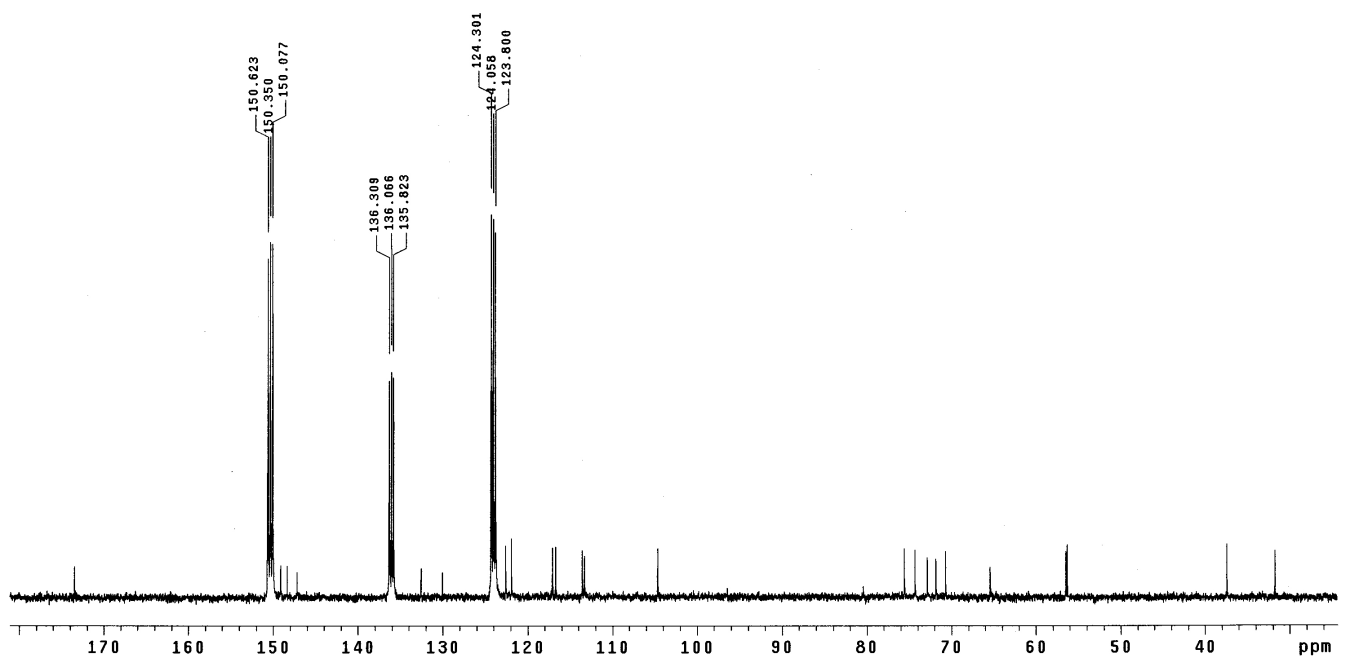


^13^C NMR spectrum of (4-hydroxy-3-methoxyphenyl)methyl-6-*O*-dihydroferuloyl-β-D-galactopyranoside (**9**)


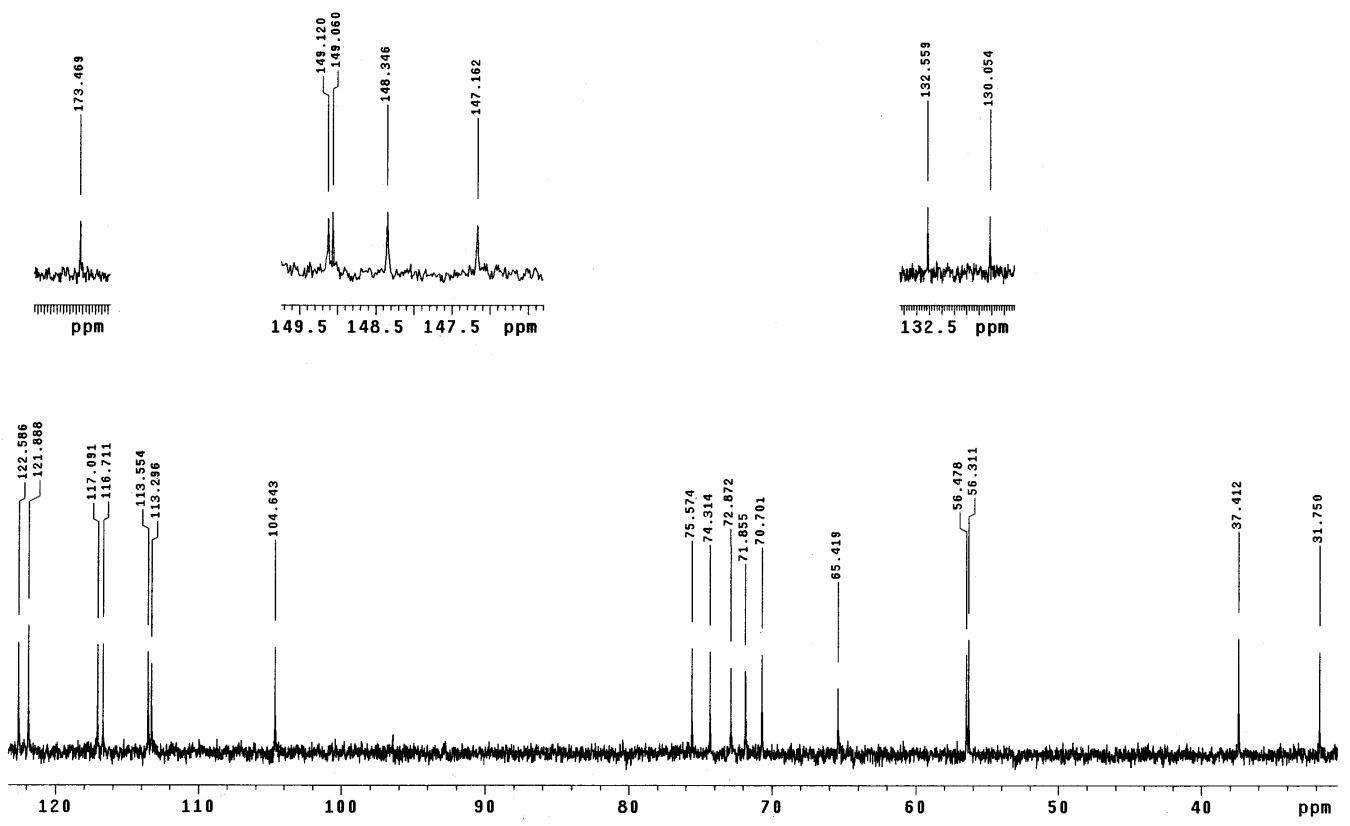


^13^C NMR spectrum of (4-hydroxy-3-methoxyphenyl)methyl-6-*O*-dihydroferuloyl-β-D-galactopyranoside (**9**)


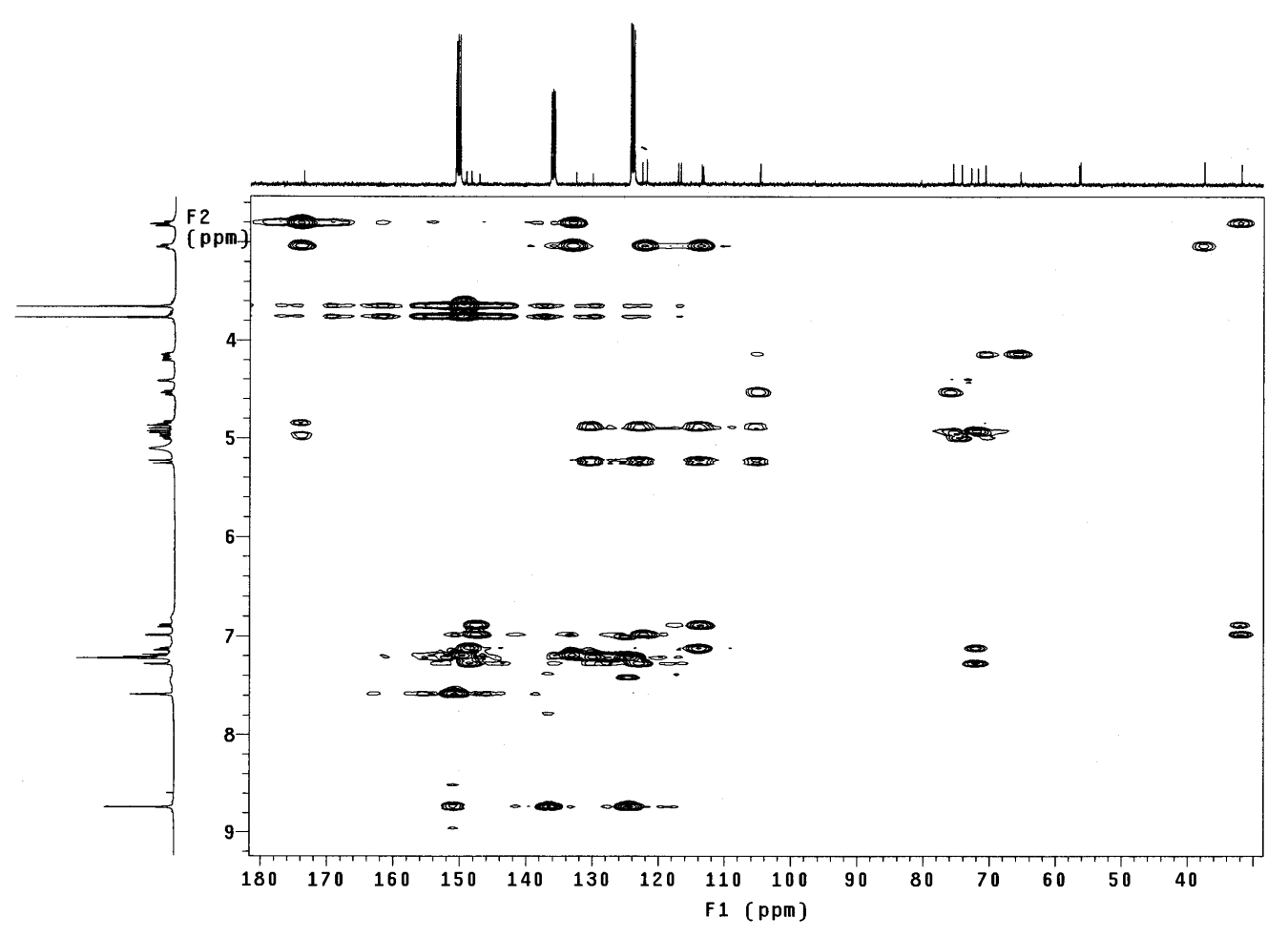


HMBC spectrum of (4-hydroxy-3-methoxyphenyl)methyl-6-*O*-dihydroferuloyl-β-D-galactopyranoside (**9**)

Mass spectrum of (4-hydroxy-3-methoxyphenyl)methyl-6-*O*-dihydroferuloyl-β-D-galactopyranoside (**9**)

(4-hydroxy-3-methoxyphenyl)methyl-6-*O*-dihydrocaffeoyl-β-D-galactopyranoside (**10**)


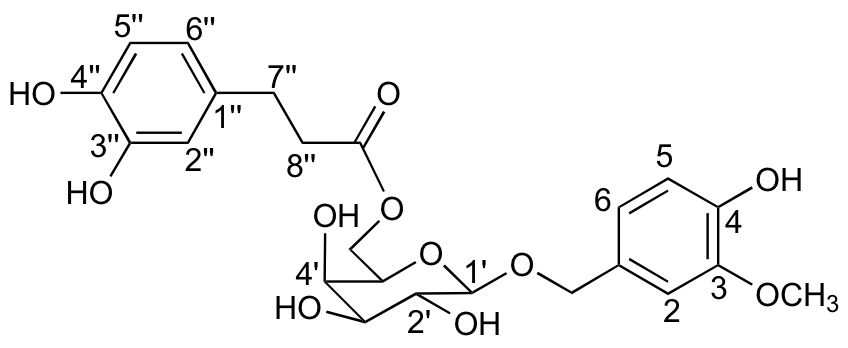


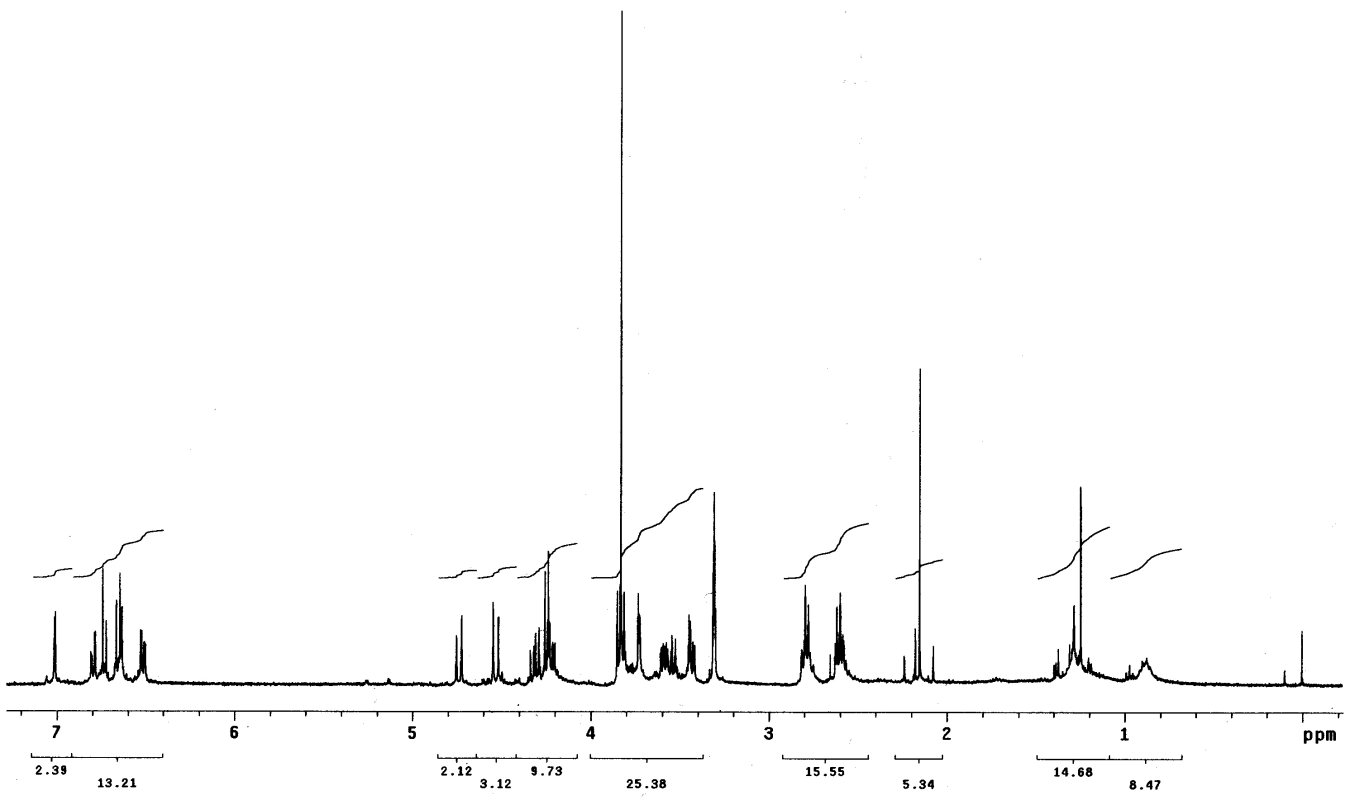


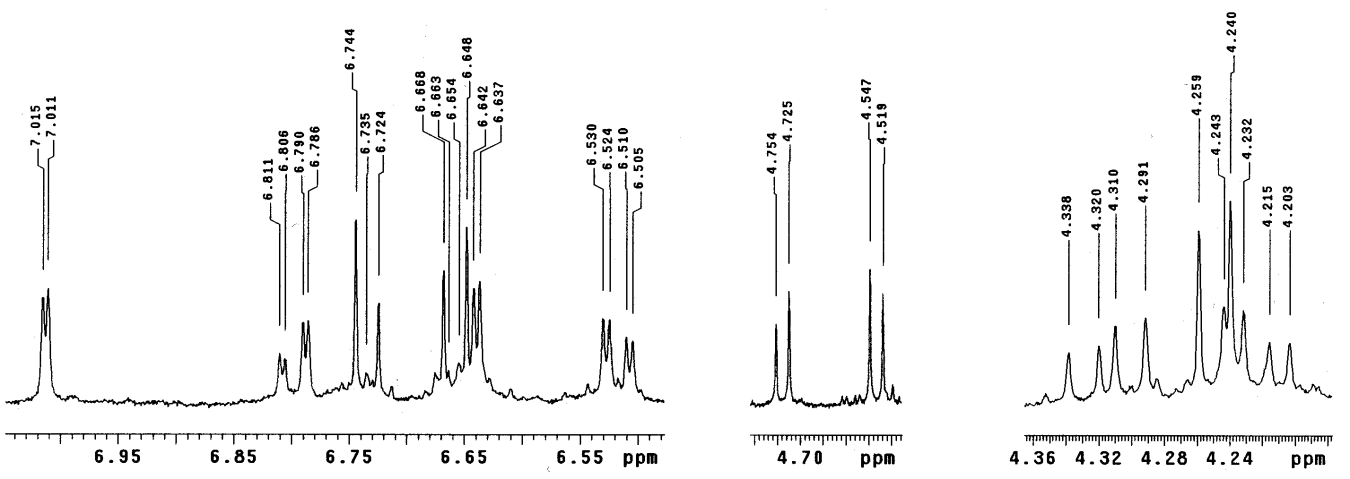


^1^H NMR spectrum of (4-hydroxy-3-methoxyphenyl)methyl-6-*O*-dihydrocaffeoyl-β-D-galactopyranoside (**10**)


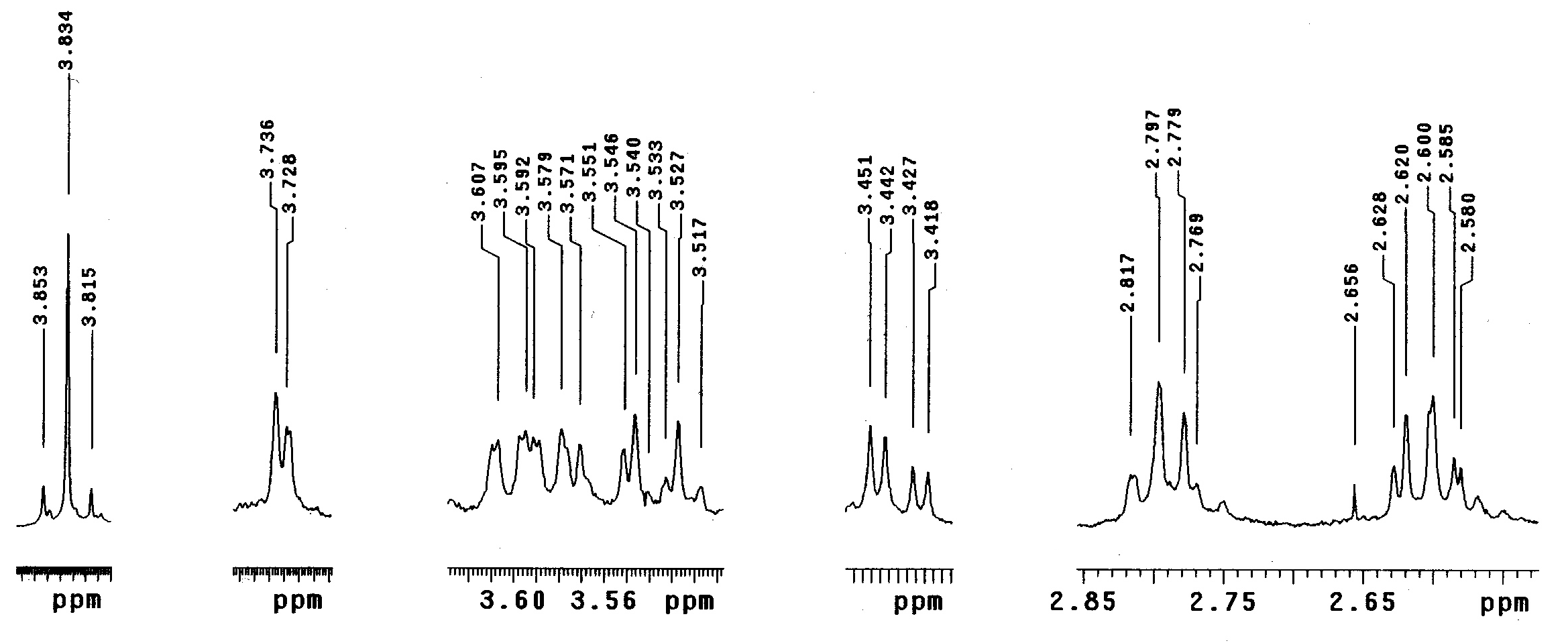


^1^H NMR spectrum of (4-hydroxy-3-methoxyphenyl)methyl-6-*O*-dihydrocaffeoyl-β-D-galactopyranoside (**10**)


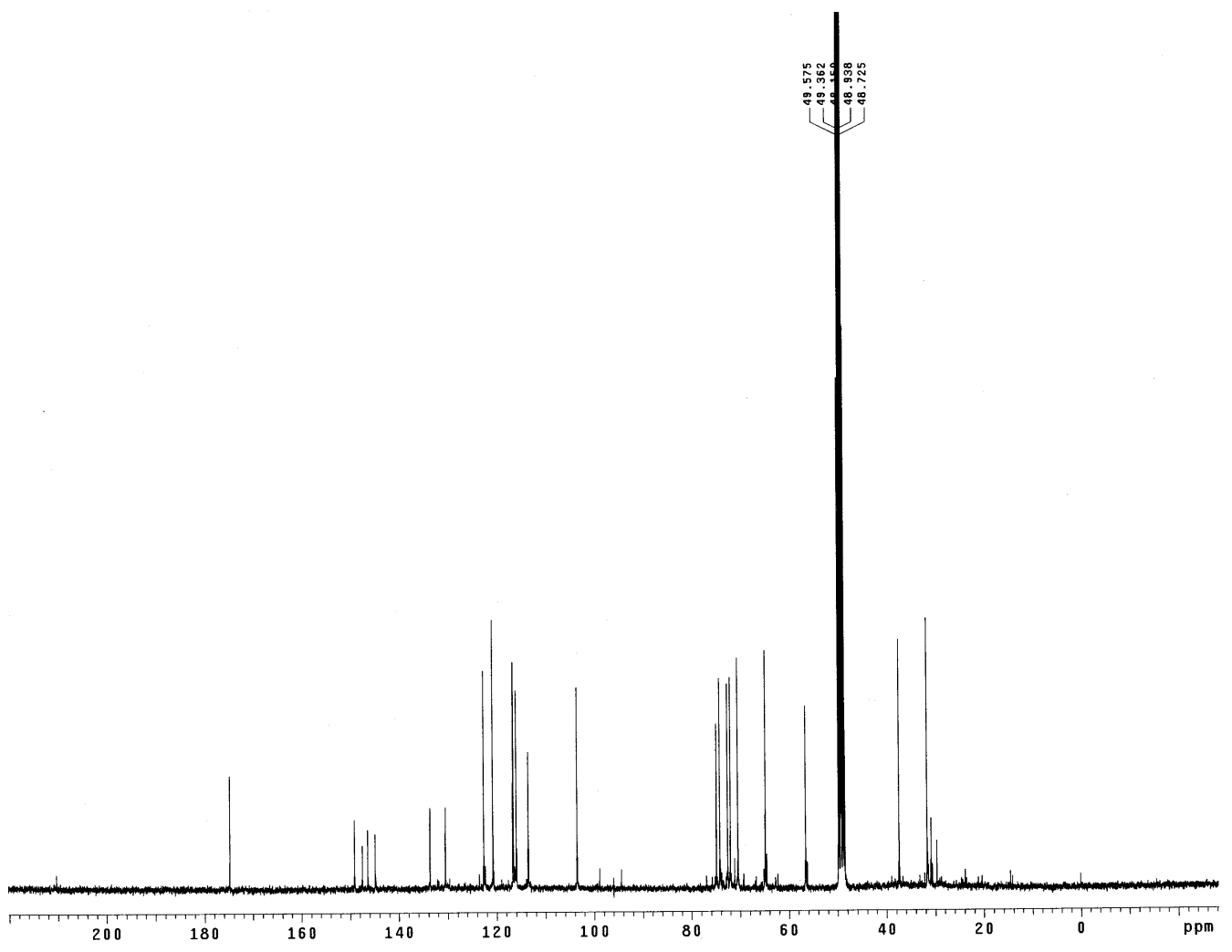


^13^C NMR spectrum of (4-hydroxy-3-methoxyphenyl)methyl-6-*O*-dihydrocaffeoyl-β-D-galactopyranoside (**10**)


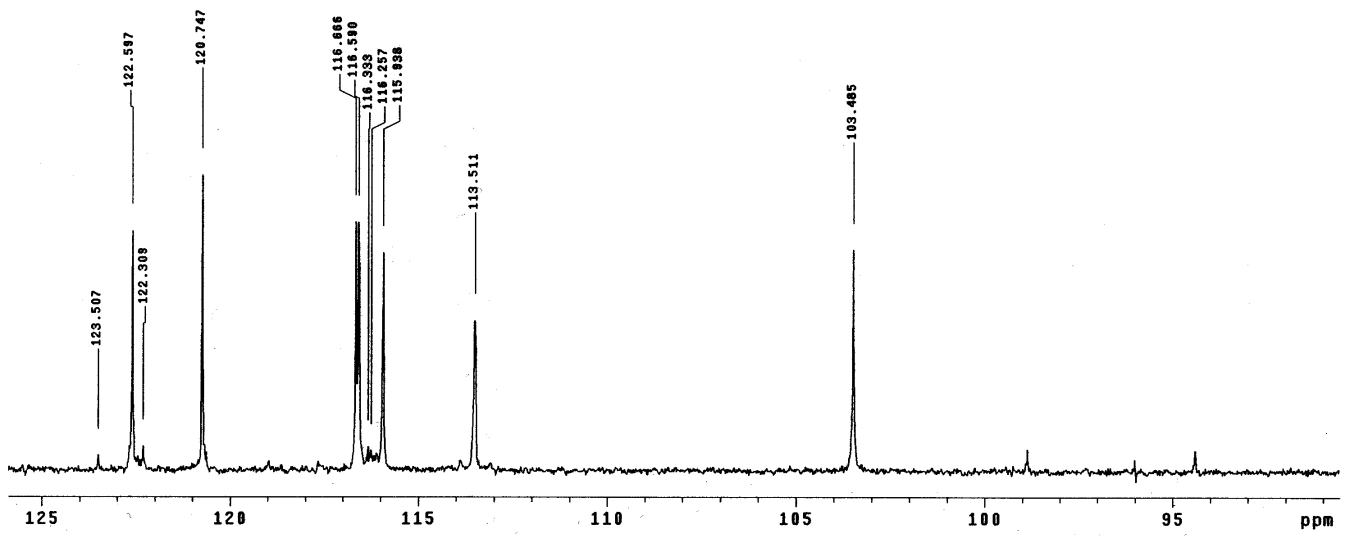


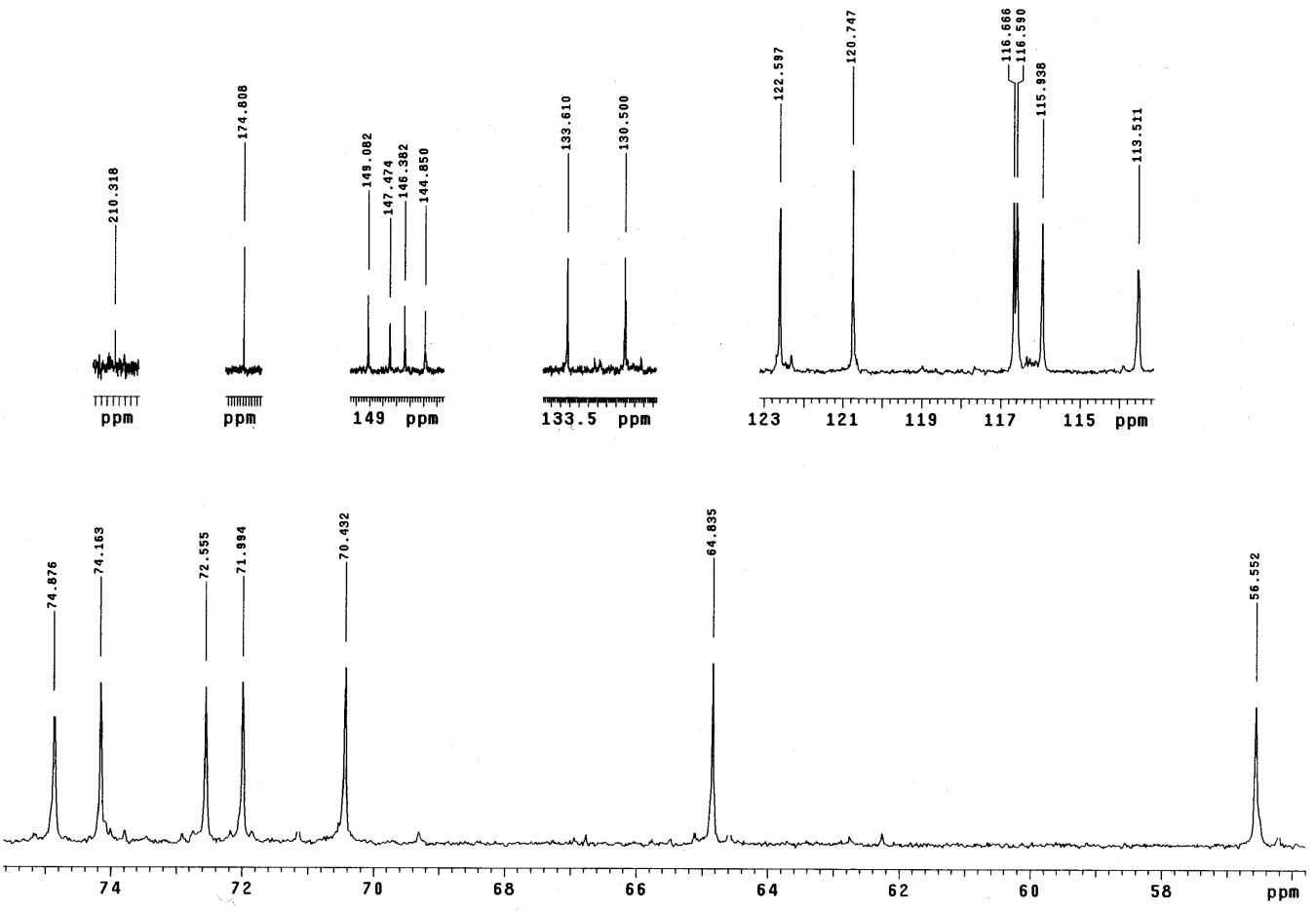


^13^C NMR spectrum of (4-hydroxy-3-methoxyphenyl)methyl-6-*O*-dihydrocaffeoyl-β-D-galactopyranoside (**10**)


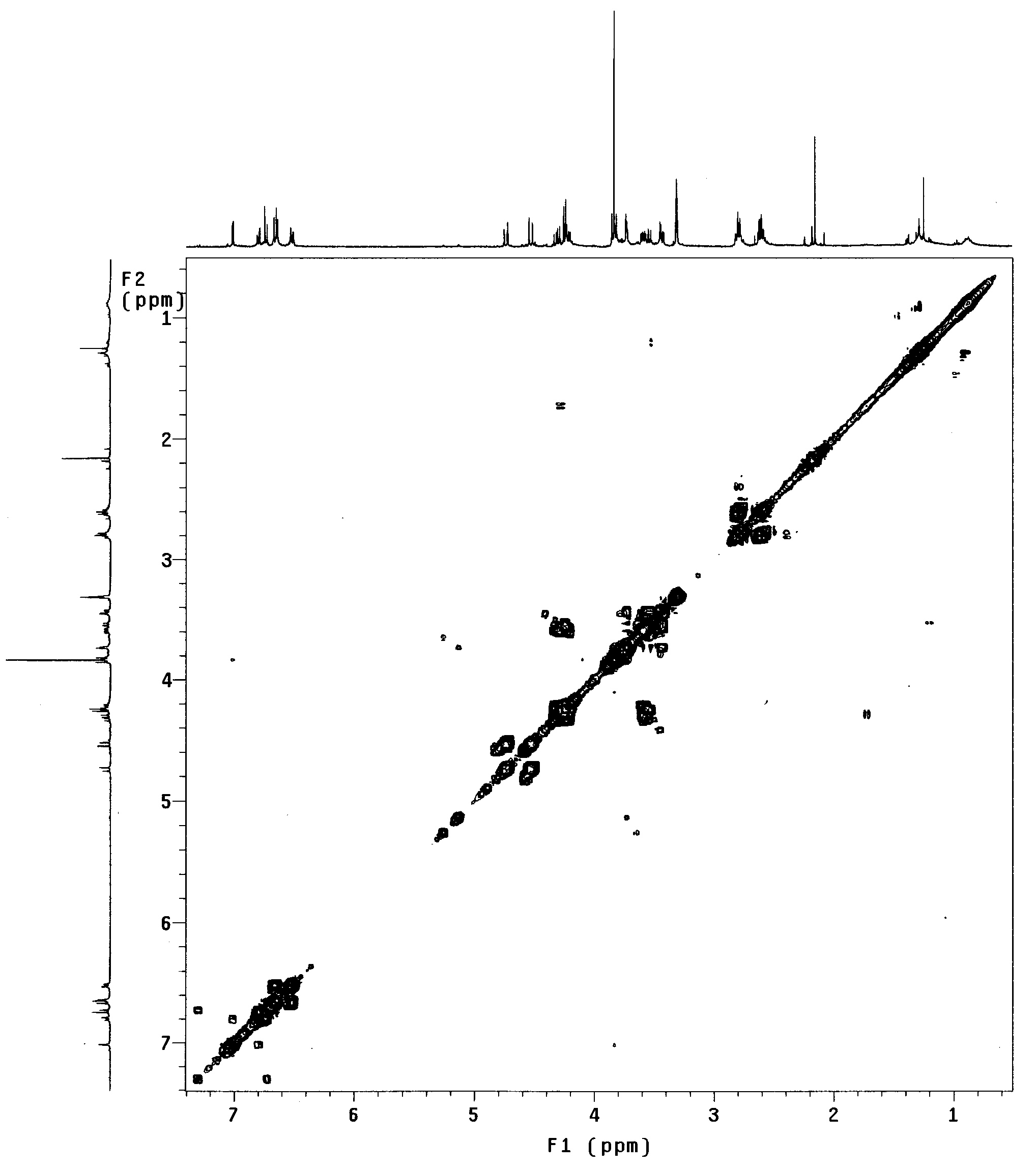


COSY spectrum of (4-hydroxy-3-methoxyphenyl)methyl-6-*O*-dihydrocaffeoyl-β-D-galactopyranoside (**10**)


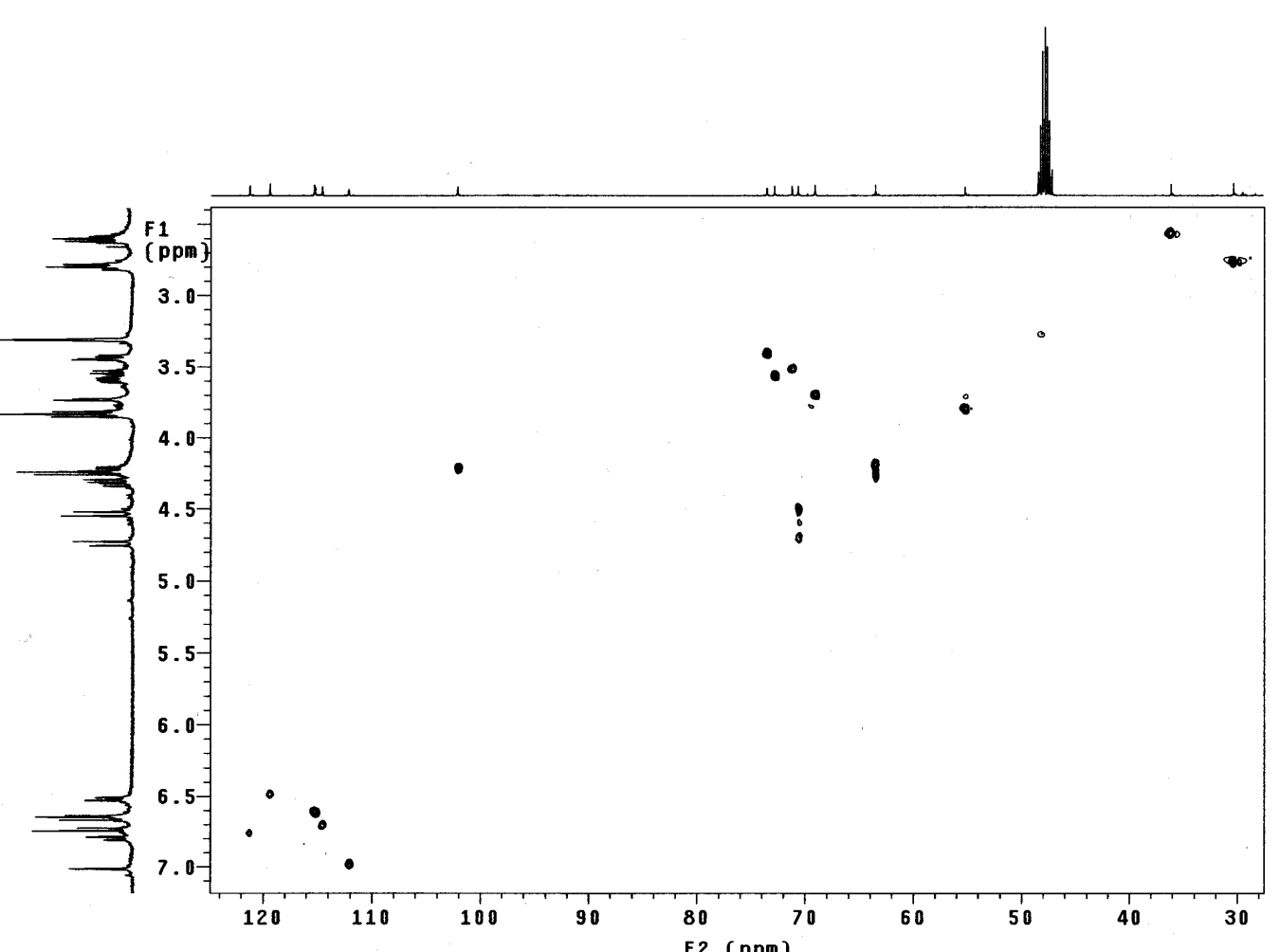


HETCOR spectrum of (4-hydroxy-3-methoxyphenyl)methyl-6-*O*-dihydrocaffeoyl-β-D-galactopyranoside (**10**)

Mass spectrum of (4-hydroxy-3-methoxyphenyl)methyl-6-*O*-dihydrocaffeoyl-β-D-galactopyranoside (**10**)

Mass spectrum of (4-hydroxy-3-methoxyphenyl)methyl-6-*O*-dihydrocaffeoyl-β-D-galactopyranoside (**10**)

2-(4-hydroxy-3-methoxyphenyl)ethyl-6-*O*-dihydroferuloyl-β-D-galactopyranoside (**12**)


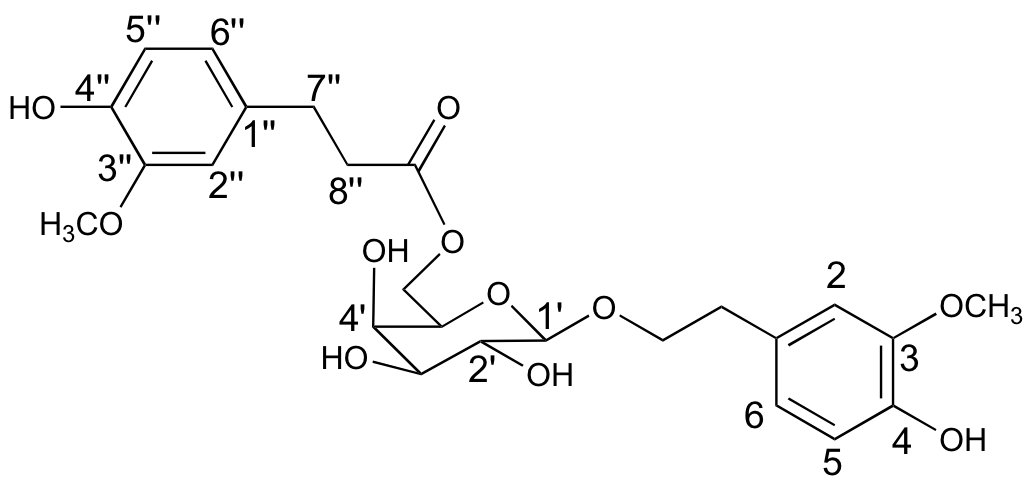


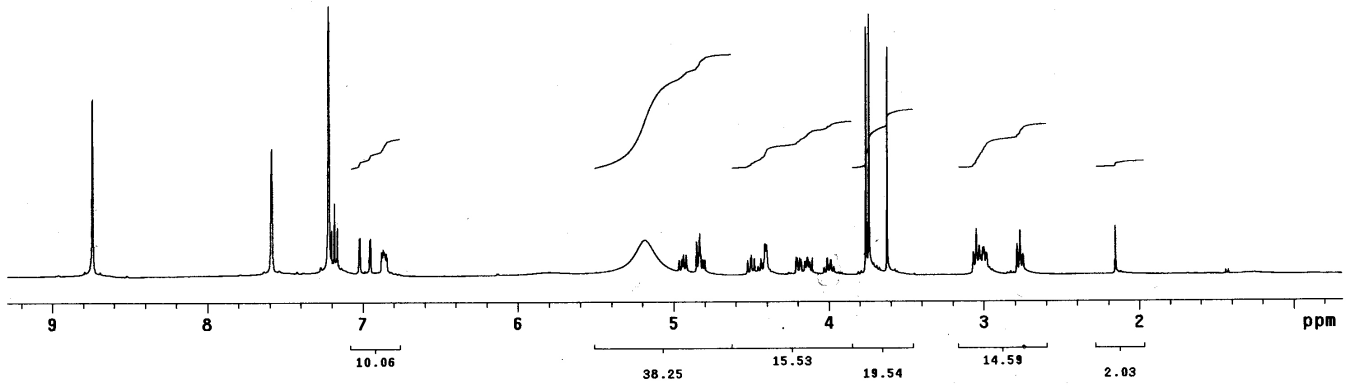


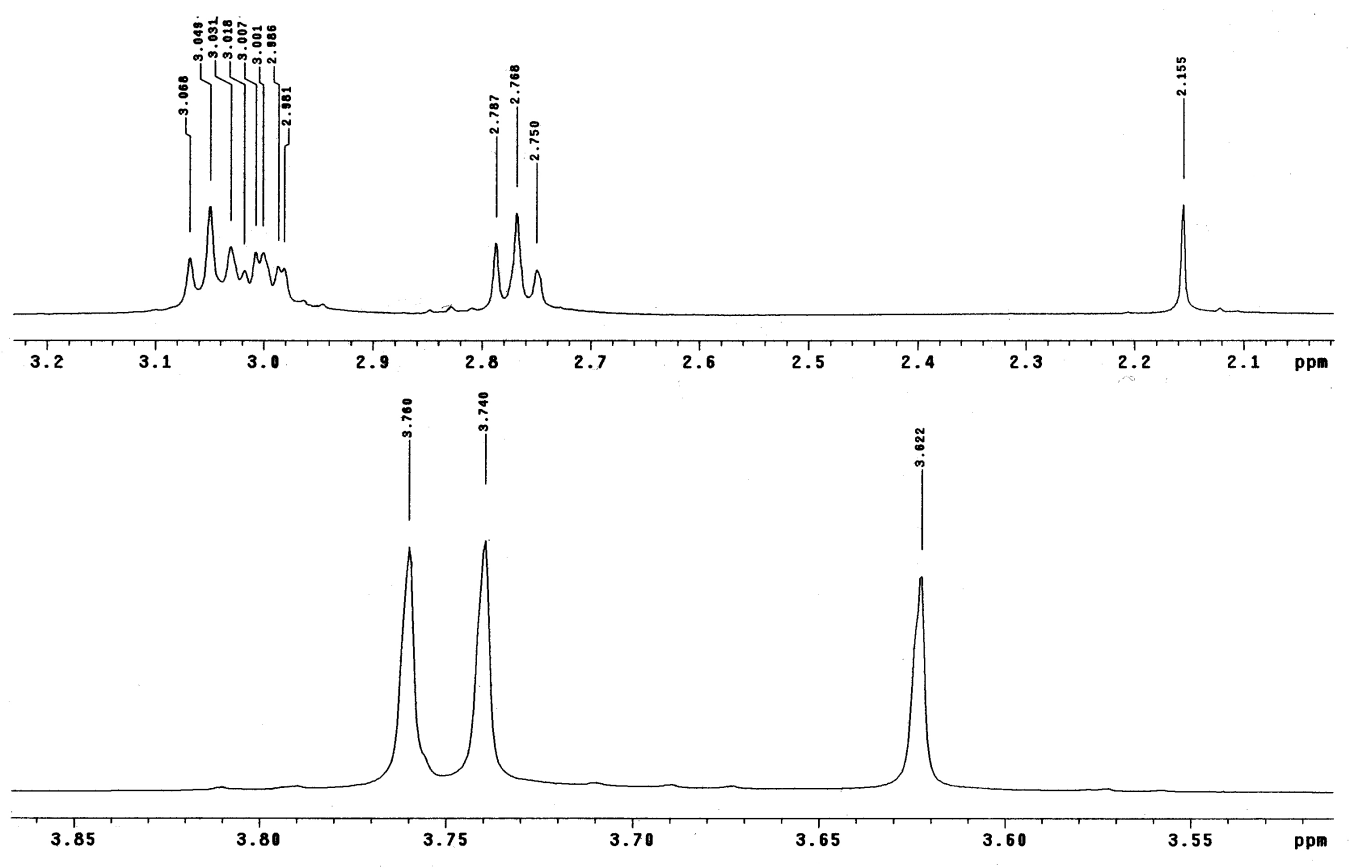


^1^H NMR spectrum of 2-(4-hydroxy-3-methoxyphenyl)ethyl-6-*O*-dihydroferuloyl-β-D-galactopyranoside (**12**)


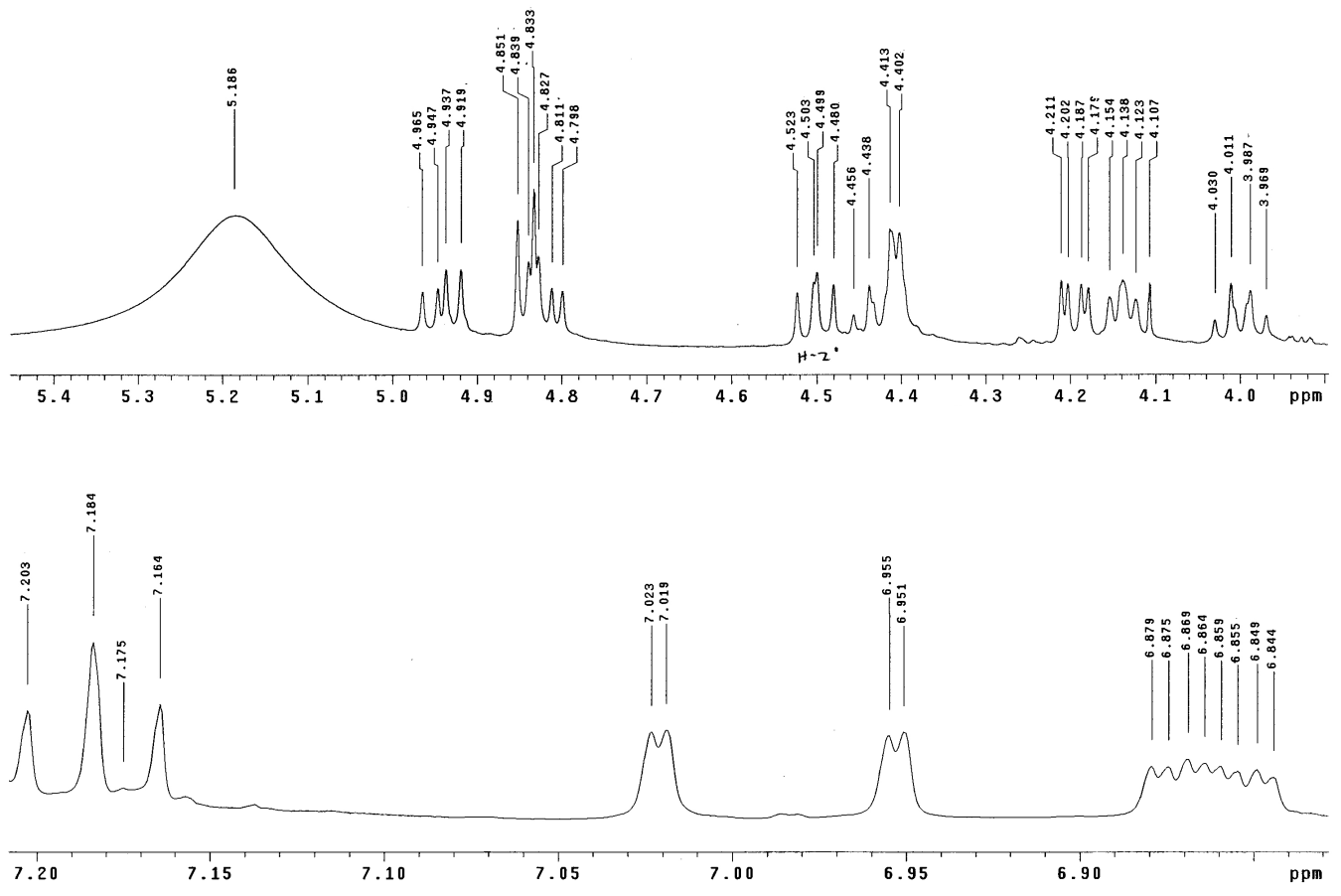


^1^H NMR spectrum of 2-(4-hydroxy-3-methoxyphenyl)ethyl-6-*O*-dihydroferuloyl-β-D-galactopyranoside (**12**)


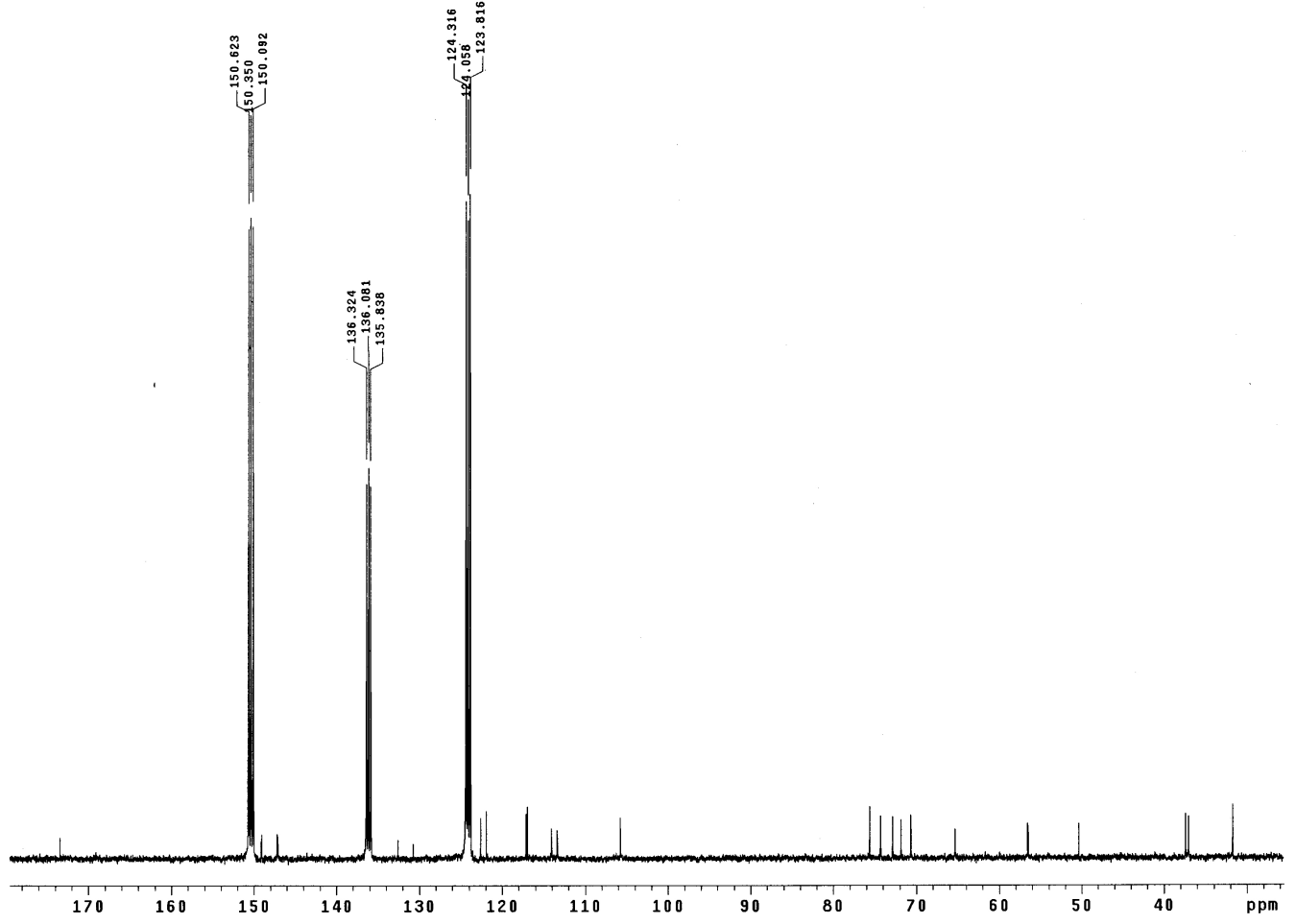


^13^C NMR spectrum of 2-(4-hydroxy-3-methoxyphenyl)ethyl-6-*O*-dihydroferuloyl-β-D-galactopyranoside (**12**)


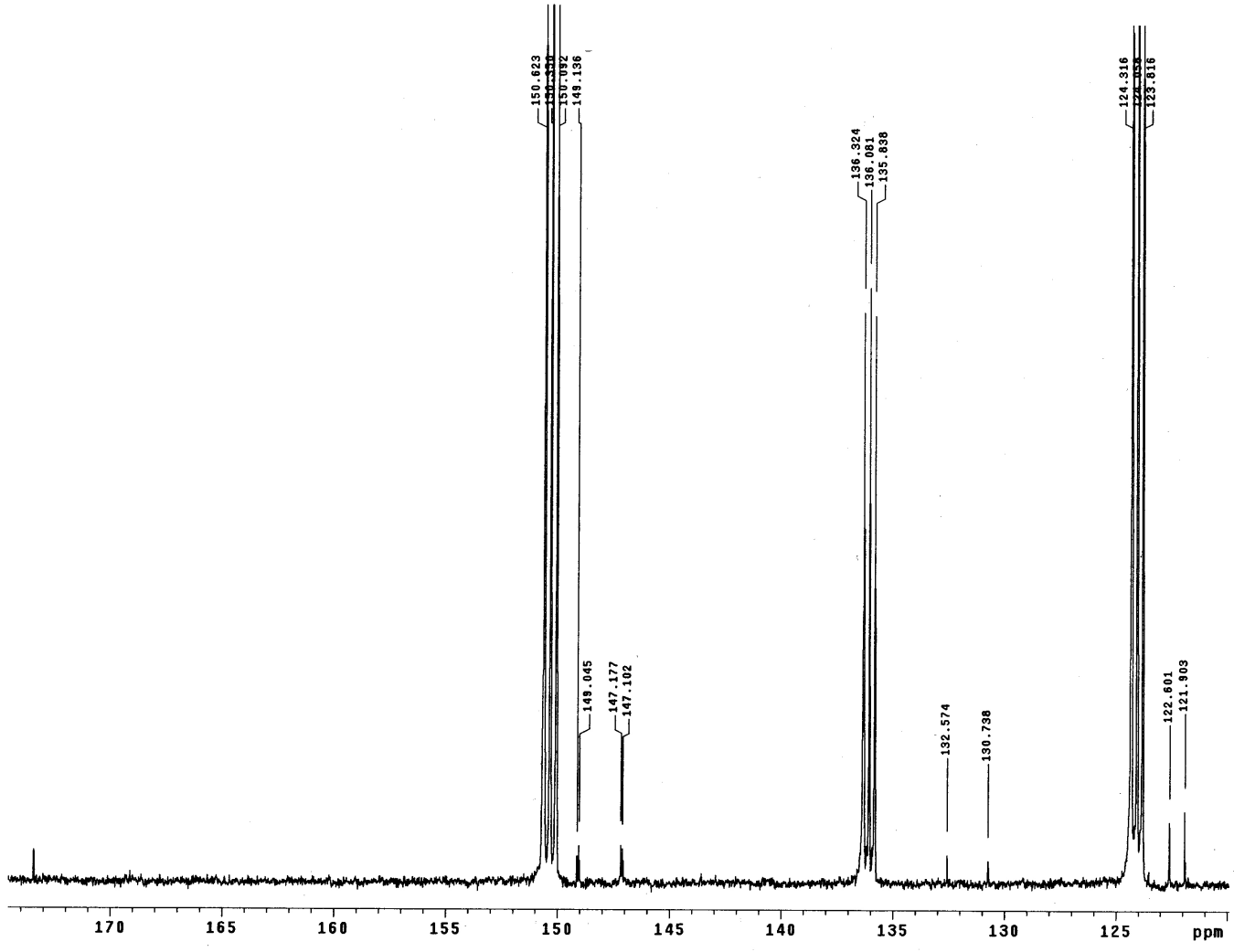


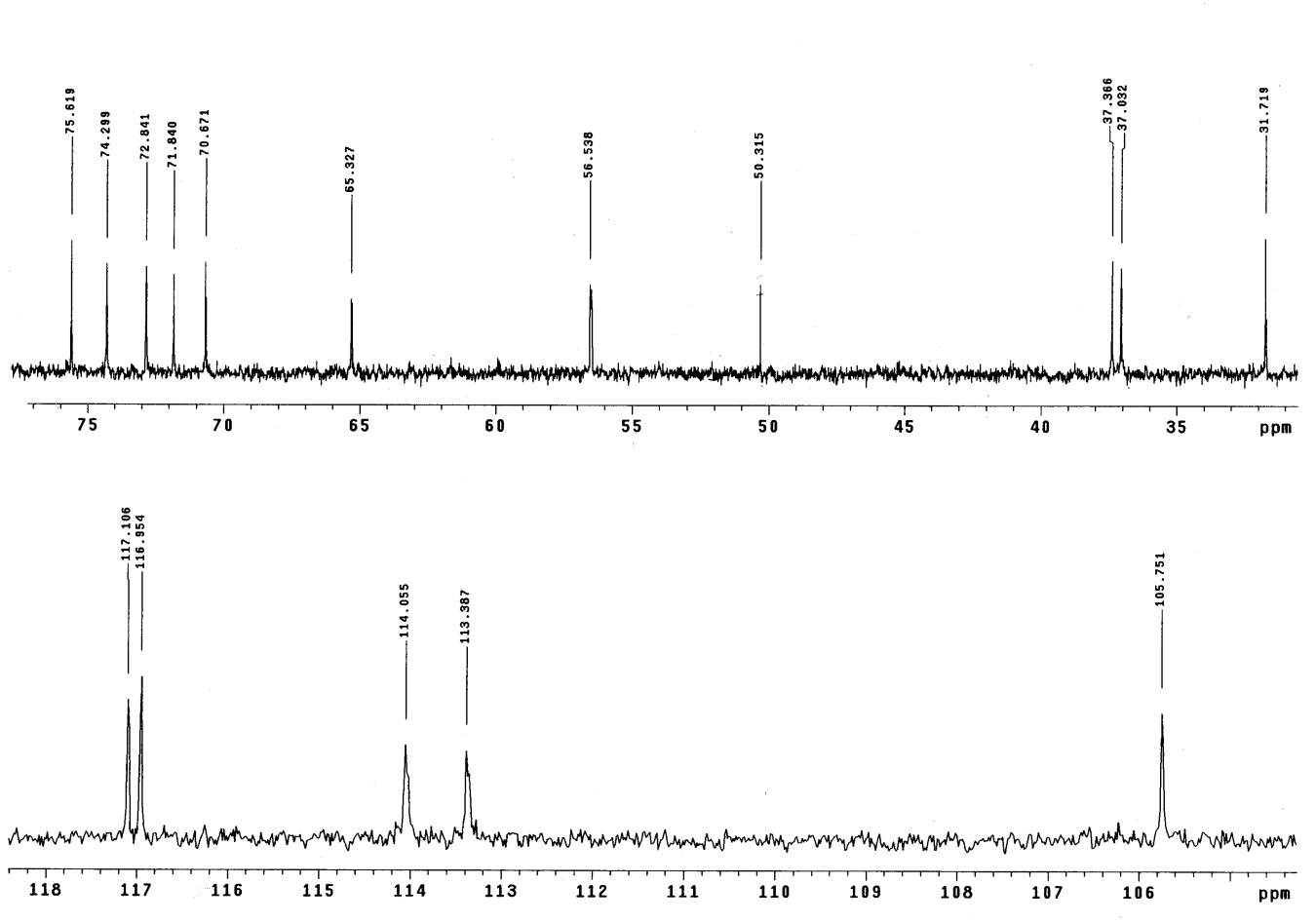


^13^C NMR spectrum of 2-(4-hydroxy-3-methoxyphenyl)ethyl-6-*O*-dihydroferuloyl-β-D-galactopyranoside (**12**)


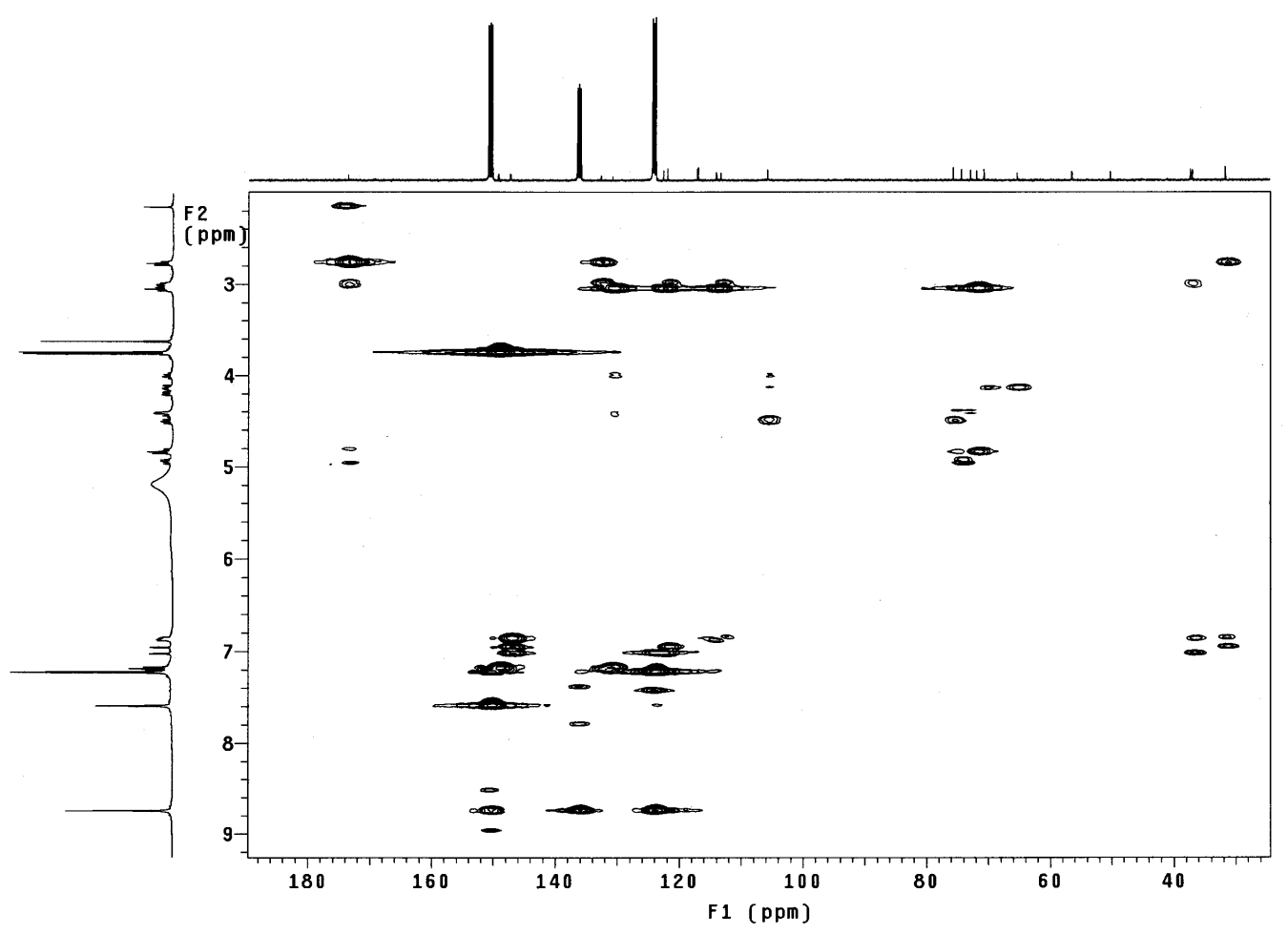


HMBC spectrum of 2-(4-hydroxy-3-methoxyphenyl)ethyl-6-*O*-dihydroferuloyl-β-D-galactopyranoside (**12**)

Mass spectrum of 2-(4-hydroxy-3-methoxyphenyl)ethyl-6-*O*-dihydroferuloyl-β-D-galactopyranoside (**12**)

2-(4-hydroxy-3-methoxyphenyl)ethyl-6-*O*-dihydrocaffeoyl-β-D-galactopyranoside (**13**)


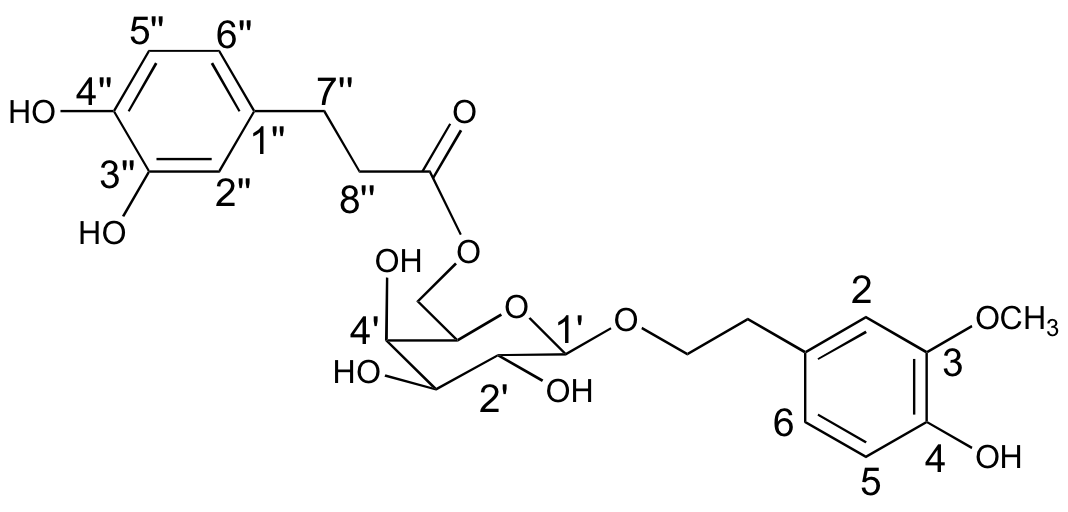


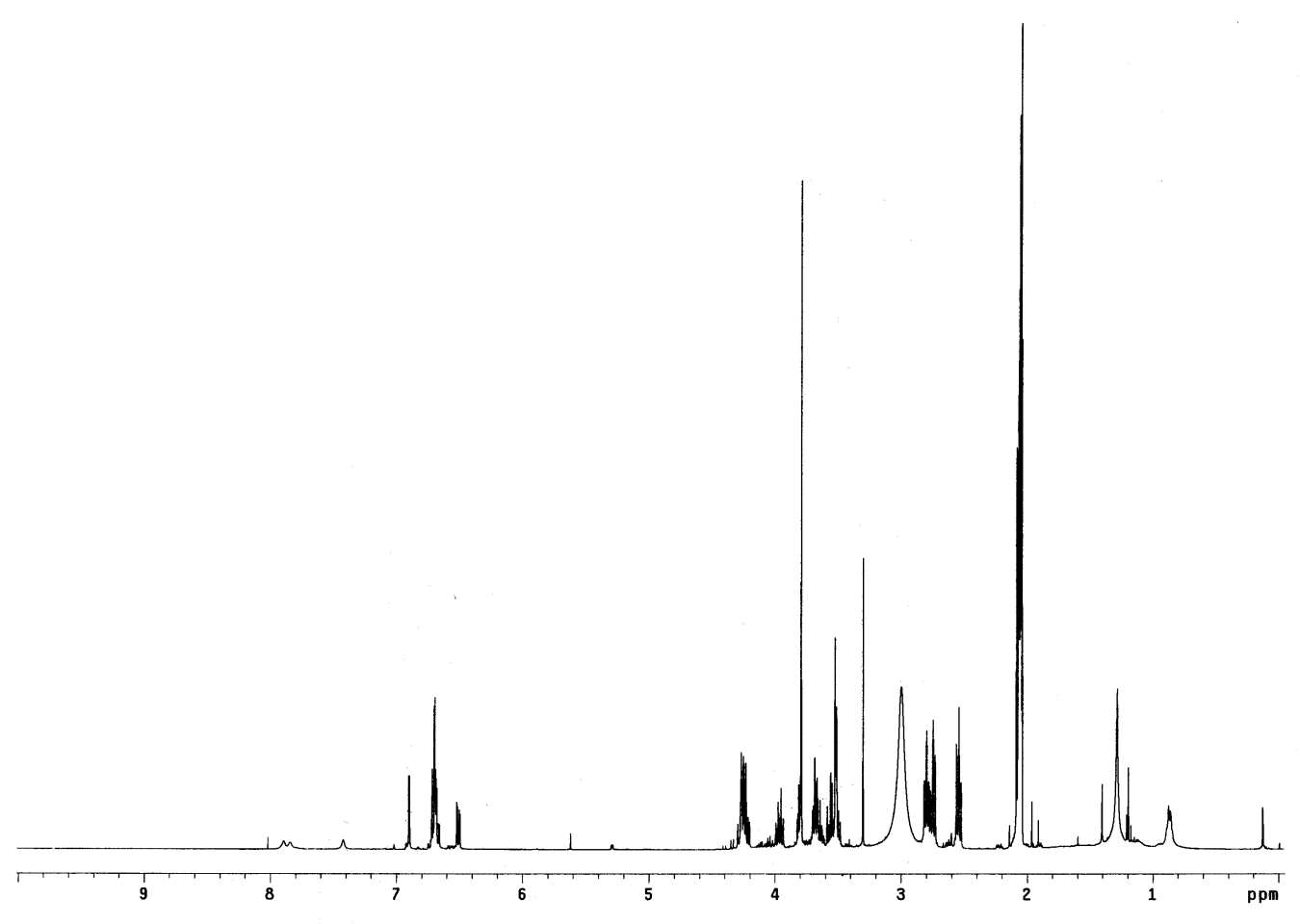


^1^H NMR spectrum of 2-(4-hydroxy-3-methoxyphenyl)ethyl-6-*O*-dihydrocaffeoyl-β-D-galactopyranoside (**13**)


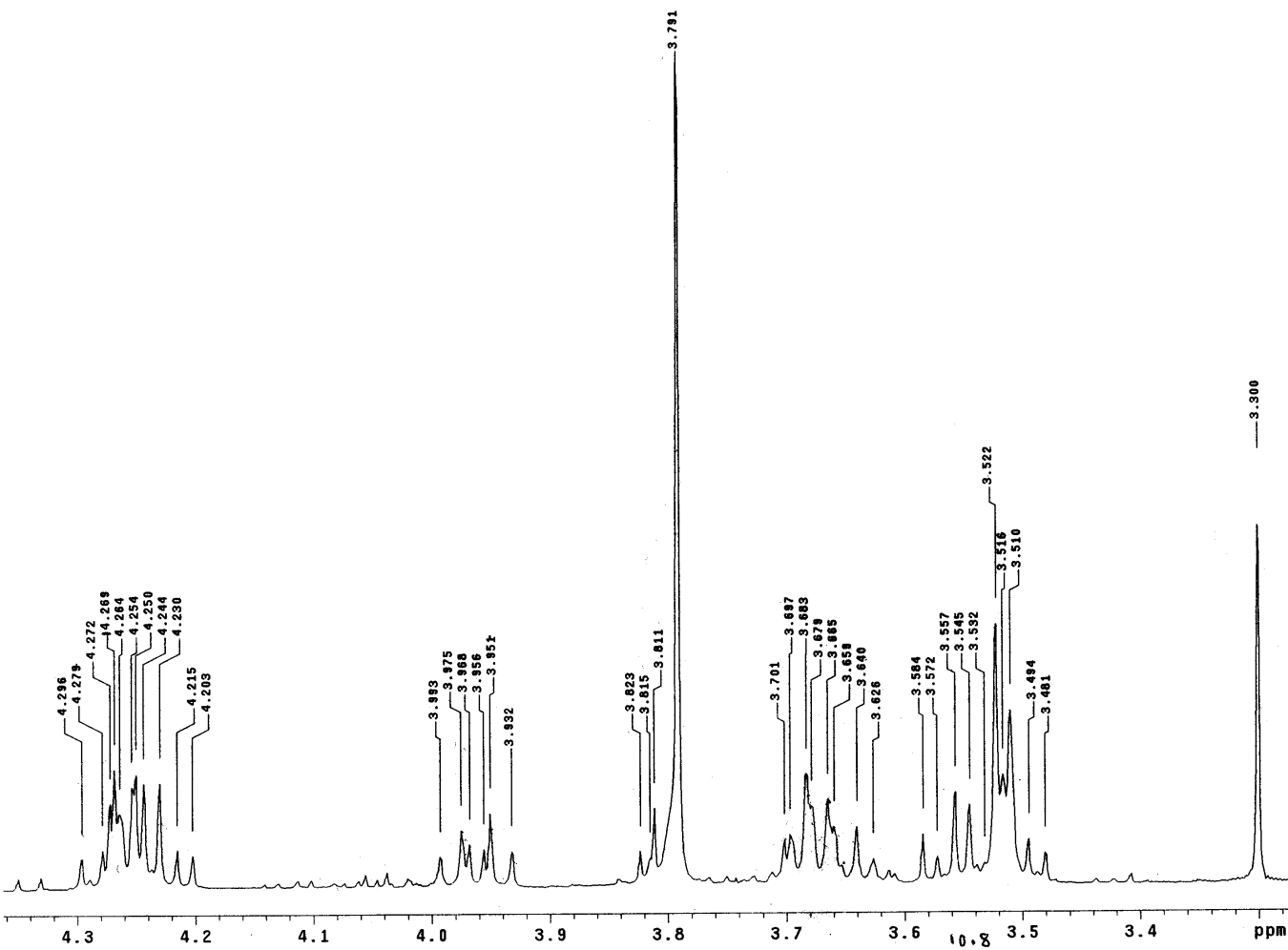


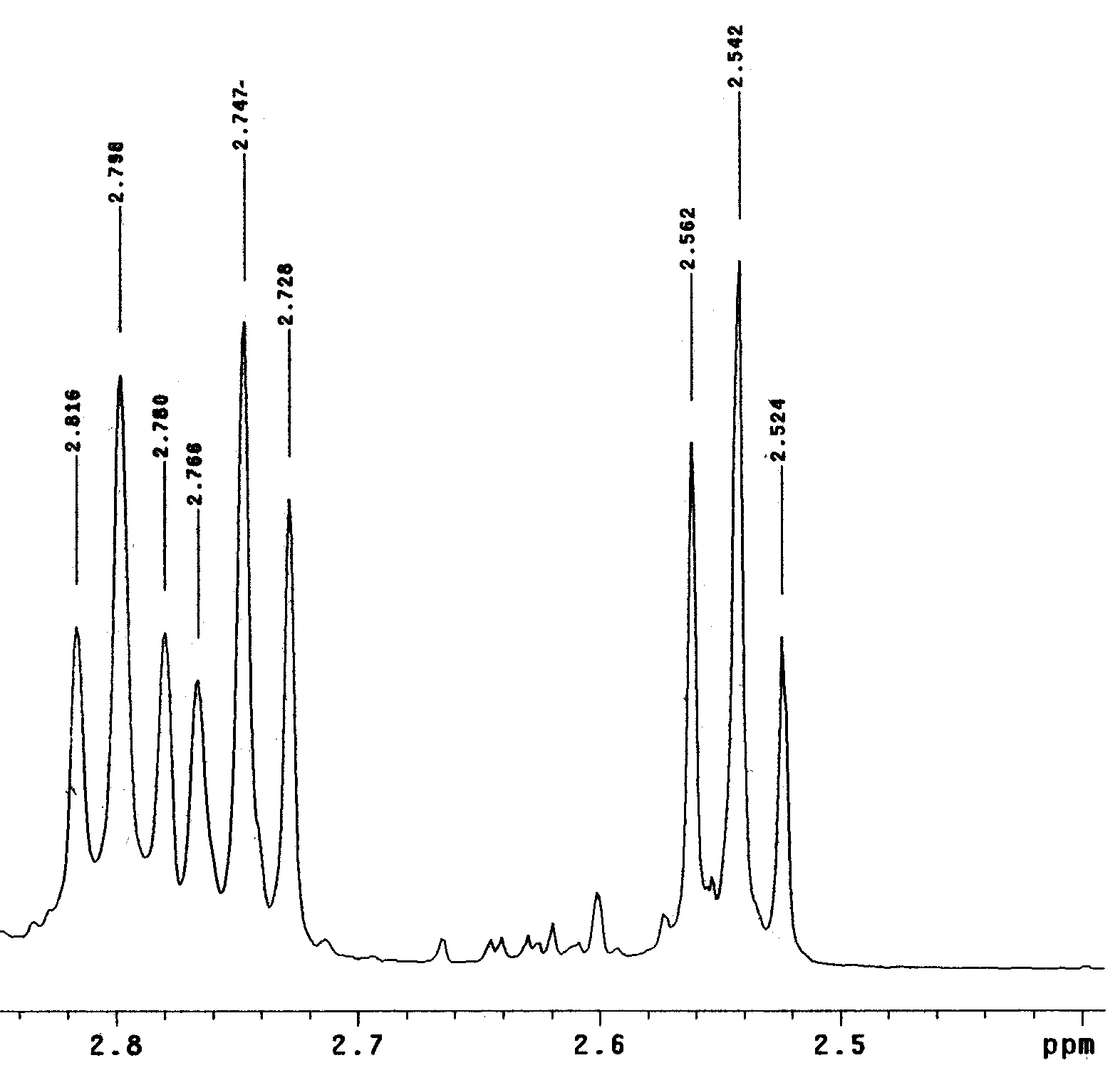


^1^H NMR spectrum of 2-(4-hydroxy-3-methoxyphenyl)ethyl-6-*O*-dihydrocaffeoyl-β-D-galactopyranoside (**13**)


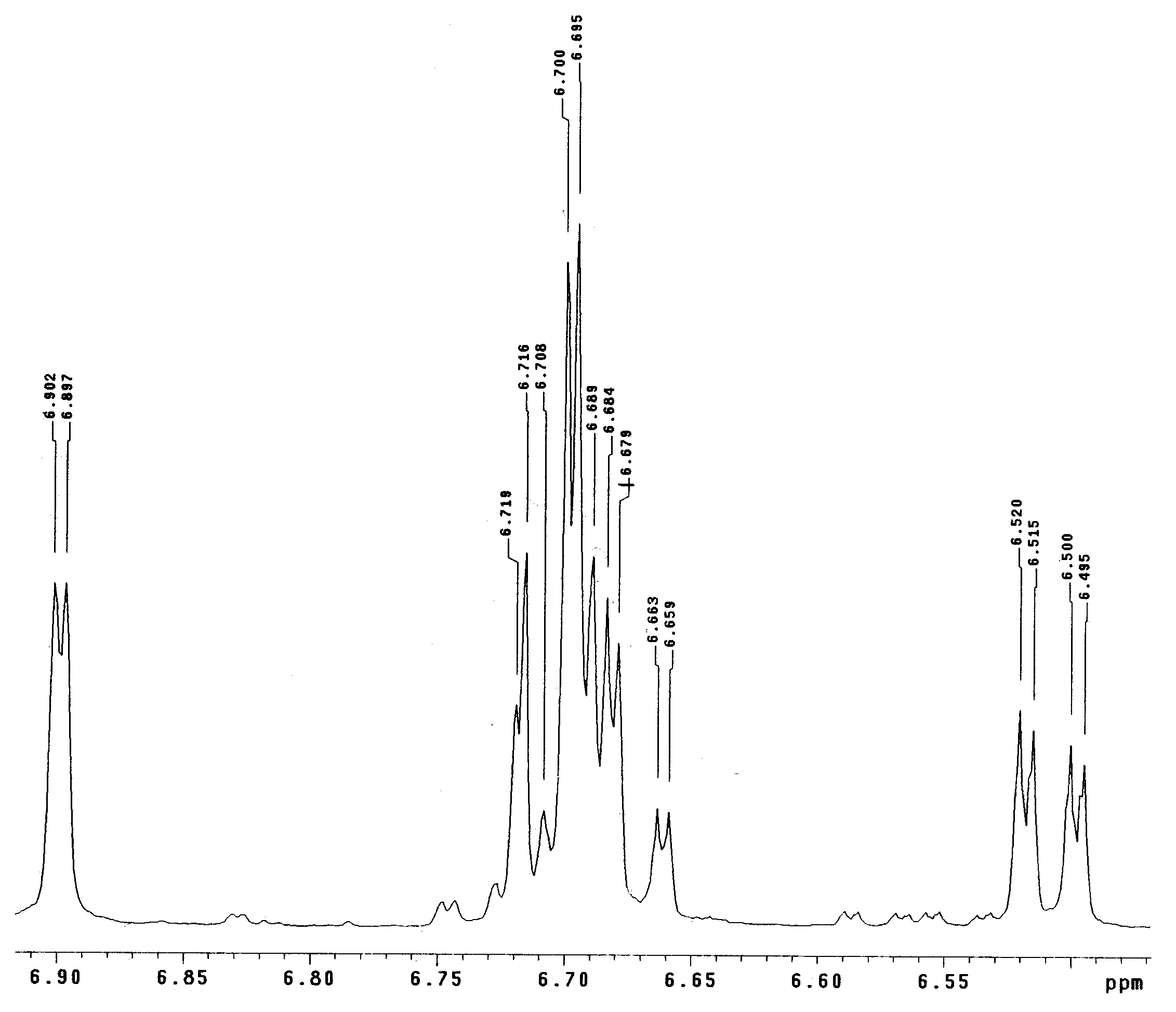


^1^H NMR spectrum of 2-(4-hydroxy-3-methoxyphenyl)ethyl-6-*O*-dihydrocaffeoyl-β-D-galactopyranoside (**13**)


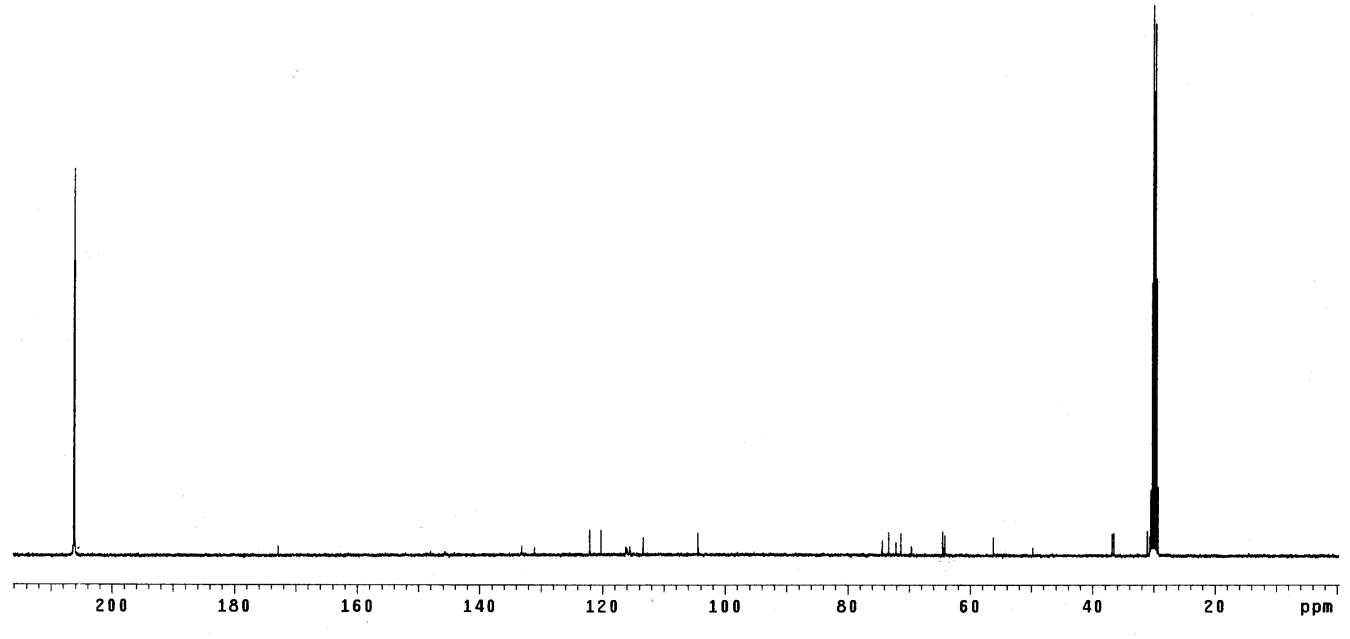


^13^C NMR spectrum of 2-(4-hydroxy-3-methoxyphenyl)ethyl-6-*O*-dihydrocaffeoyl-β-D-galactopyranoside (**13**)


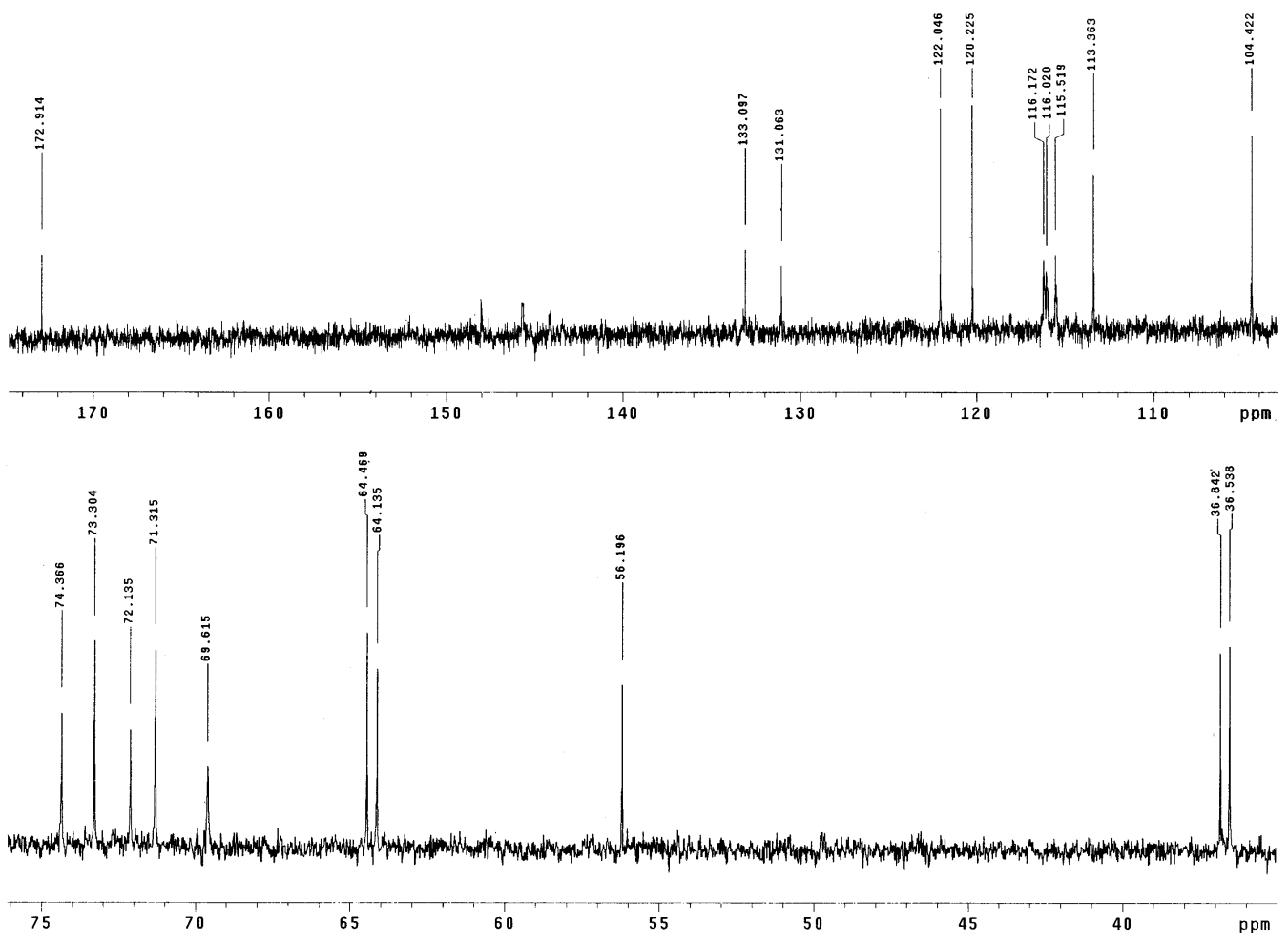


^13^C NMR spectrum of 2-(4-hydroxy-3-methoxyphenyl)ethyl-6-*O*-dihydrocaffeoyl-β-D-galactopyranoside (**13**)


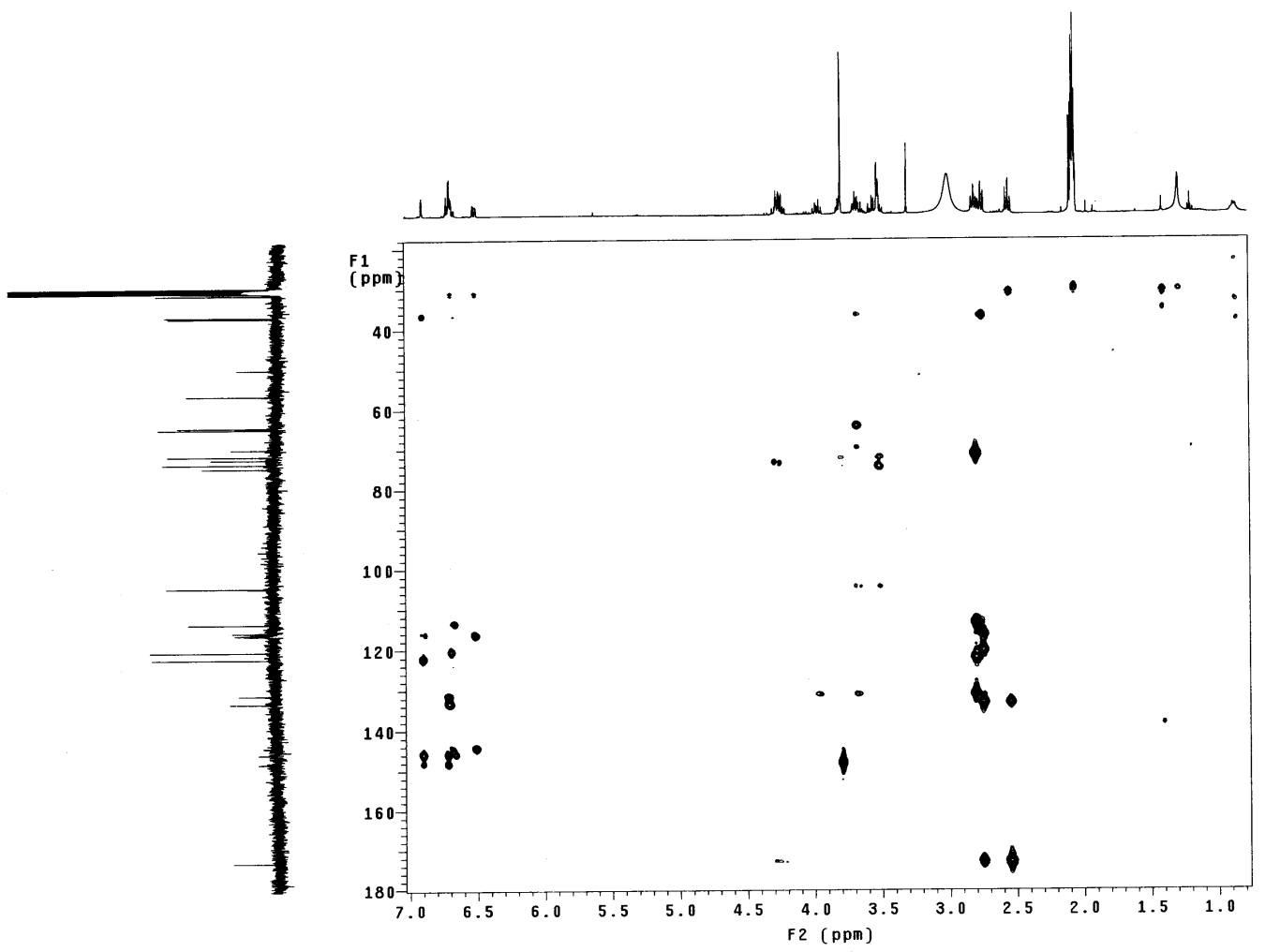


HMBC spectrum of 2-(4-hydroxy-3-methoxyphenyl)ethyl-6-*O*-dihydrocaffeoyl-β-D-galactopyranoside (**13**)

Mass spectrum of 2-(4-hydroxy-3-methoxyphenyl)ethyl-6-*O*-dihydrocaffeoyl-β-D-galactopyranoside (**13**)

Optimized structures

**Compound 5**

Input orientation:

---------------------------------------------------------------------

Center Atomic Atomic Coordinates (Angstroms)

Number Number Type X Y Z

---------------------------------------------------------------------

1 6 0 0.054587 0.343257 -0.041145

2 6 0 -0.088776 -0.252985 1.219781

3 6 0 1.021827 -0.503668 2.013066

4 6 0 2.309170 -0.170149 1.571292

5 6 0 2.440656 0.419494 0.312814

6 6 0 1.327019 0.674969 -0.486775

7 1 0 0.887733 -0.974215 2.985658

8 1 0 3.427960 0.678334 -0.058181

9 1 0 1.428769 1.127274 -1.467136

10 8 0 -1.035385 0.586699 -0.821102

11 1 0 -1.819584 0.279266 -0.347235

12 8 0 -1.385880 -0.555568 1.573878

13 1 0 -1.407479 -0.987503 2.433964

14 6 0 3.512783 -0.413916 2.453841

15 1 0 3.343503 -1.278663 3.100782

16 1 0 4.381765 -0.668021 1.840619

17 6 0 3.856054 0.800783 3.327175

18 1 0 3.002132 1.088208 3.950800

19 1 0 4.071388 1.681731 2.714703

20 6 0 5.033096 0.557257 4.242507

21 8 0 5.373524 1.681919 4.918416

22 1 0 6.123255 1.452882 5.488933

23 8 0 5.618119 -0.485642 4.389646

---------------------------------------------------------------------

**Compound (5-H)^-1^**

Input orientation:

---------------------------------------------------------------------

Center Atomic Atomic Coordinates (Angstroms)

Number Number Type X Y Z

---------------------------------------------------------------------

1 6 0 -0.035984 -0.129999 0.015705

2 6 0 0.083900 -0.065789 1.408181

3 6 0 1.329429 -0.018556 2.013933

4 6 0 2.509974 -0.027492 1.254285

5 6 0 2.373745 -0.093542 -0.136295

6 6 0 1.120299 -0.144466 -0.750945

7 1 0 1.399207 0.028356 3.099818

8 1 0 3.267393 -0.103802 -0.753714

9 1 0 1.023847 -0.194969 -1.831228

10 8 0 -1.277506 -0.180179 -0.574443

11 1 0 -1.928640 -0.139947 0.137731

12 8 0 -1.116519 -0.062894 2.105723

13 1 0 -0.932196 0.045815 3.044292

14 6 0 3.849514 0.054772 1.942448

15 1 0 3.867554 -0.565311 2.843905

16 1 0 4.626385 -0.348322 1.281943

17 6 0 4.257398 1.463323 2.405518

18 1 0 3.385951 1.968608 2.846182

19 1 0 4.582057 2.088733 1.567840

20 6 0 5.386050 1.450797 3.515405

21 8 0 6.088159 2.481168 3.553539

22 8 0 5.413625 0.428856 4.241571

---------------------------------------------------------------------

**Compound (5-2H)^-2^**

Input orientation:

---------------------------------------------------------------------

Center Atomic Atomic Coordinates (Angstroms)

Number Number Type X Y Z

---------------------------------------------------------------------

1 6 0 -0.017685 -0.094908 -0.036985

2 6 0 -0.043109 0.027504 1.403218

3 6 0 1.094605 0.112277 2.177322

4 6 0 2.383407 0.079884 1.585678

5 6 0 2.433538 -0.039683 0.197580

6 6 0 1.276453 -0.121437 -0.600594

7 1 0 0.993371 0.202341 3.258727

8 1 0 3.411044 -0.074935 -0.286035

9 1 0 1.357553 -0.217416 -1.684158

10 8 0 -1.159833 -0.172018 -0.644047

11 8 0 -1.323227 0.041444 1.911813

12 6 0 3.644479 0.150515 2.430898

13 1 0 3.564414 -0.553454 3.273423

14 1 0 4.506342 -0.195958 1.852723

15 6 0 4.020796 1.530260 3.001805

16 1 0 3.249050 1.897914 3.687804

17 1 0 4.050381 2.254121 2.173042

18 6 0 5.410742 1.601200 3.738088

19 8 0 5.508515 2.499706 4.610770

20 8 0 6.287485 0.782383 3.362762

21 1 0 -1.823487 -0.038950 1.058745

---------------------------------------------------------------------

**Compound 6**

Input orientation:

---------------------------------------------------------------------

Center Atomic Atomic Coordinates (Angstroms)

Number Number Type X Y Z

---------------------------------------------------------------------

1 6 0 0.084499 0.346186 0.068181

2 6 0 -0.023725 -0.180370 1.369038

3 6 0 1.116476 -0.424275 2.123435

4 6 0 2.391158 -0.150780 1.601463

5 6 0 2.483347 0.367037 0.311040

6 6 0 1.340897 0.614955 -0.451844

7 1 0 1.031129 -0.839450 3.121663

8 1 0 3.459808 0.575955 -0.115969

9 1 0 1.409502 1.012264 -1.458547

10 8 0 -1.033664 0.581296 -0.672549

11 1 0 -1.798088 0.323824 -0.139351

12 8 0 -1.320312 -0.411718 1.763307

13 6 0 3.626109 -0.385839 2.442662

14 1 0 3.487350 -1.256326 3.089884

15 1 0 4.476935 -0.627314 1.800146

16 6 0 3.986092 0.827953 3.310172

17 1 0 3.154479 1.103494 3.967803

18 1 0 4.166346 1.713989 2.692724

19 6 0 5.204554 0.599304 4.173476

20 8 0 5.514053 1.706065 4.893155

21 1 0 6.297884 1.489346 5.421017

22 8 0 5.846684 -0.417443 4.247071

23 6 0 -1.551071 -0.954322 3.054029

24 1 0 -1.176459 -0.287616 3.837658

25 1 0 -2.630702 -1.053307 3.150323

26 1 0 -1.085181 -1.939455 3.159612

---------------------------------------------------------------------

**Compound (6-H)^-1^**

Input orientation:

---------------------------------------------------------------------

Center Atomic Atomic Coordinates (Angstroms)

Number Number Type X Y Z

---------------------------------------------------------------------

1 6 0 -0.042466 -0.082215 -0.015332

2 6 0 0.039096 -0.030899 1.386029

3 6 0 1.271690 0.021857 2.020145

4 6 0 2.468360 0.030239 1.280110

5 6 0 2.367368 -0.022550 -0.111271

6 6 0 1.127879 -0.078414 -0.755941

7 1 0 1.336601 0.061117 3.101810

8 1 0 3.275144 -0.017753 -0.707873

9 1 0 1.055473 -0.118804 -1.838541

10 8 0 -1.269320 -0.137643 -0.632946

11 1 0 -1.931076 -0.117399 0.071019

12 8 0 -1.195529 -0.046580 2.015444

13 6 0 3.791152 0.118171 2.000670

14 1 0 3.802194 -0.528284 2.883634

15 1 0 4.590311 -0.249882 1.346270

16 6 0 4.159838 1.520334 2.513135

17 1 0 3.270115 1.991262 2.955601

18 1 0 4.481596 2.178101 1.699465

19 6 0 5.271635 1.499868 3.639123

20 8 0 5.956012 2.540378 3.713236

21 8 0 5.305633 0.461140 4.340947

22 6 0 -1.218389 0.056209 3.427549

23 1 0 -0.748255 0.984615 3.768265

24 1 0 -2.269802 0.054303 3.715060

25 1 0 -0.711784 -0.792944 3.898756

---------------------------------------------------------------------

**Compound (6-2H)^-2^**

Input orientation:

---------------------------------------------------------------------

Center Atomic Atomic Coordinates (Angstroms)

Number Number Type X Y Z

---------------------------------------------------------------------

1 6 0 -0.022763 -0.080113 -0.039820

2 6 0 -0.046680 0.027242 1.404067

3 6 0 1.103900 0.104989 2.177773

4 6 0 2.398005 0.088083 1.627815

5 6 0 2.458223 -0.031081 0.232870

6 6 0 1.314781 -0.117542 -0.559257

7 1 0 0.972718 0.188934 3.256843

8 1 0 3.438692 -0.061050 -0.246409

9 1 0 1.397448 -0.203877 -1.643546

10 8 0 -1.071680 -0.141514 -0.769868

11 8 0 -1.271544 0.110215 2.088082

12 6 0 3.646583 0.170518 2.489512

13 1 0 3.494398 -0.409442 3.411799

14 1 0 4.489717 -0.313070 1.986329

15 6 0 4.123393 1.580134 2.886986

16 1 0 3.374009 2.088763 3.504439

17 1 0 4.217833 2.187712 1.973939

18 6 0 5.507815 1.638541 3.634937

19 8 0 5.665021 2.626809 4.394144

20 8 0 6.323248 0.717328 3.376957

21 6 0 -2.161211 -0.955623 1.809695

22 1 0 -2.324968 -1.035849 0.732460

23 1 0 -1.771597 -1.913045 2.198040

24 1 0 -3.101006 -0.727315 2.328839

---------------------------------------------------------------------

**Compound 7**

Input orientation:

---------------------------------------------------------------------

Center Atomic Atomic Coordinates (Angstroms)

Number Number Type X Y Z

---------------------------------------------------------------------

1 6 0 0.082762 0.100632 -0.089783

2 6 0 -0.003796 0.063730 1.316090

3 6 0 1.135920 0.019698 2.092486

4 6 0 2.412412 0.010761 1.492620

5 6 0 2.482488 0.047834 0.092278

6 6 0 1.334322 0.092248 -0.691505

7 1 0 1.039019 -0.008125 3.174266

8 1 0 3.454605 0.041725 -0.389369

9 1 0 1.389046 0.120899 -1.773716

10 8 0 -1.041941 0.143684 -0.843597

11 1 0 -1.807636 0.142623 -0.252621

12 8 0 -1.289225 0.075858 1.805101

13 6 0 3.647475 -0.034975 2.260655

14 1 0 4.566312 -0.036662 1.678005

15 6 0 3.795512 -0.075006 3.596338

16 1 0 2.960904 -0.077797 4.287936

17 6 0 5.138639 -0.117851 4.190604

18 8 0 5.075438 -0.154509 5.549489

19 1 0 5.990135 -0.181273 5.867431

20 8 0 6.194616 -0.122004 3.598521

21 1 0 -1.288630 0.049485 2.767656

---------------------------------------------------------------------

**Compound (7-H)^-1^**

Input orientation:

---------------------------------------------------------------------

Center Atomic Atomic Coordinates (Angstroms)

Number Number Type X Y Z

---------------------------------------------------------------------

1 6 0 0.042990 0.000018 0.019947

2 6 0 -0.012570 0.000001 1.420573

3 6 0 1.158203 -0.000026 2.159321

4 6 0 2.422164 -0.000036 1.535443

5 6 0 2.447520 -0.000017 0.130342

6 6 0 1.272350 0.000008 -0.620429

7 1 0 1.078622 -0.000038 3.240417

8 1 0 3.406610 -0.000023 -0.377708

9 1 0 1.316353 0.000019 -1.708875

10 8 0 -1.187070 0.000041 -0.629884

11 8 0 -1.230664 0.000011 2.052872

12 6 0 3.687035 -0.000061 2.276390

13 1 0 4.600084 -0.000067 1.681307

14 6 0 3.876845 -0.000078 3.602069

15 1 0 3.043362 -0.000075 4.305034

16 6 0 5.276291 -0.000117 4.254474

17 8 0 5.245853 -0.000007 5.507185

18 8 0 6.251638 0.000036 3.469201

19 1 0 -1.910233 0.000042 1.366790

20 1 0 -1.040683 0.000129 -1.580732

---------------------------------------------------------------------

**Compound (7-2H)^-2^**

Input orientation:

---------------------------------------------------------------------

Center Atomic Atomic Coordinates (Angstroms)

Number Number Type X Y Z

---------------------------------------------------------------------

1 6 0 -0.024909 0.000003 -0.015234

2 6 0 -0.015439 -0.000001 1.434286

3 6 0 1.134508 -0.000004 2.186334

4 6 0 2.415168 -0.000005 1.561887

5 6 0 2.426269 -0.000001 0.159378

6 6 0 1.256834 0.000003 -0.612802

7 1 0 1.057029 -0.000007 3.270918

8 1 0 3.393108 -0.000001 -0.345132

9 1 0 1.309728 0.000005 -1.701886

10 8 0 -1.175119 0.000006 -0.600569

11 8 0 -1.282631 -0.000001 1.972465

12 6 0 3.681910 -0.000009 2.300022

13 1 0 4.583855 -0.000009 1.684149

14 6 0 3.917916 -0.000011 3.624288

15 1 0 3.097700 -0.000009 4.343950

16 6 0 5.315193 -0.000009 4.255584

17 8 0 5.321535 -0.000139 5.517388

18 8 0 6.305480 -0.000151 3.477529

19 1 0 -1.809383 0.000001 1.134567

---------------------------------------------------------------------

**Compound 8**

Input orientation:

---------------------------------------------------------------------

Center Atomic Atomic Coordinates (Angstroms)

Number Number Type X Y Z

---------------------------------------------------------------------

1 6 0 0.118908 0.028651 -0.033635

2 6 0 0.060147 -0.136445 1.369348

3 6 0 1.218482 -0.148434 2.121759

4 6 0 2.477251 0.004116 1.499214

5 6 0 2.516012 0.165979 0.108089

6 6 0 1.350998 0.178726 -0.652904

7 1 0 1.164372 -0.275753 3.195191

8 1 0 3.474974 0.284125 -0.385564

9 1 0 1.378706 0.303989 -1.729248

10 8 0 -1.021771 0.039030 -0.763159

11 1 0 -1.765351 -0.078708 -0.155056

12 8 0 -1.218613 -0.273357 1.845437

13 6 0 3.727826 -0.000456 2.242907

14 1 0 4.631044 0.125481 1.649385

15 6 0 3.908776 -0.140679 3.568112

16 1 0 3.093217 -0.273182 4.269471

17 6 0 5.263015 -0.118802 4.136322

18 8 0 5.233233 -0.276211 5.488182

19 1 0 6.153875 -0.251903 5.788590

20 8 0 6.303067 0.017550 3.531446

21 6 0 -1.409121 -0.446639 3.243283

22 1 0 -1.032089 0.415417 3.802390

23 1 0 -2.483636 -0.532687 3.391037

24 1 0 -0.918176 -1.358096 3.598125

---------------------------------------------------------------------

**Compound (8-H)^-1^**

Input orientation:

---------------------------------------------------------------------

Center Atomic Atomic Coordinates (Angstroms)

Number Number Type X Y Z

---------------------------------------------------------------------

1 6 0 -0.017267 -0.000373 -0.014316

2 6 0 -0.002657 -0.000160 1.392199

3 6 0 1.192000 0.000061 2.090280

4 6 0 2.430794 0.000082 1.412254

5 6 0 2.393292 -0.000143 0.010556

6 6 0 1.188908 -0.000366 -0.695131

7 1 0 1.195018 0.000216 3.172840

8 1 0 3.330986 -0.000136 -0.536857

9 1 0 1.171624 -0.000534 -1.780622

10 8 0 -1.212441 -0.000589 -0.690932

11 1 0 -1.907493 -0.000552 -0.019375

12 8 0 -1.267841 -0.000203 1.956917

13 6 0 3.717207 0.000340 2.116091

14 1 0 4.613730 0.000253 1.496552

15 6 0 3.939026 0.000685 3.436899

16 1 0 3.122322 0.000831 4.160348

17 6 0 5.347252 0.000888 4.067366

18 8 0 5.333404 0.001103 5.321135

19 8 0 6.311129 0.000613 3.269076

20 6 0 -1.362477 -0.000040 3.369820

21 1 0 -0.895517 0.891459 3.801706

22 1 0 -2.427300 -0.000129 3.602438

23 1 0 -0.895317 -0.891326 3.801926

---------------------------------------------------------------------

**Compound (8-H)^-2^**

Input orientation:

---------------------------------------------------------------------

Center Atomic Atomic Coordinates (Angstroms)

Number Number Type X Y Z

---------------------------------------------------------------------

1 6 0 -0.034122 0.009137 -0.014003

2 6 0 -0.020415 0.020381 1.439060

3 6 0 1.142163 0.016135 2.186336

4 6 0 2.429229 0.004829 1.599781

5 6 0 2.447068 -0.018422 0.192022

6 6 0 1.288045 -0.025324 -0.573523

7 1 0 1.039801 0.034715 3.269021

8 1 0 3.416034 -0.031574 -0.309695

9 1 0 1.341130 -0.033694 -1.662661

10 8 0 -1.100585 0.028878 -0.712551

11 8 0 -1.228786 0.100878 2.147982

12 6 0 3.684395 0.003791 2.356386

13 1 0 4.595670 -0.017943 1.754431

14 6 0 3.903479 0.022686 3.683373

15 1 0 3.074698 0.045292 4.393054

16 6 0 5.293577 0.012647 4.331060

17 8 0 5.285160 0.030255 5.592676

18 8 0 6.292147 -0.012340 3.564419

19 6 0 -2.147435 -0.934106 1.846006

20 1 0 -2.351989 -0.956619 0.773375

21 1 0 -1.762832 -1.915351 2.171640

22 1 0 -3.064017 -0.716037 2.408104

---------------------------------------------------------------------

**Compound 3**

Input orientation:

---------------------------------------------------------------------

Center Atomic Atomic Coordinates (Angstroms)

Number Number Type X Y Z

---------------------------------------------------------------------

1 6 0 -0.185749 0.484837 0.094421

2 6 0 0.038469 0.027427 1.398384

3 6 0 1.325706 0.013294 1.923274

4 6 0 2.411430 0.449328 1.145051

5 6 0 2.187466 0.894716 -0.151395

6 6 0 0.896398 0.914760 -0.673081

7 1 0 -0.798666 -0.322099 1.991913

8 1 0 3.038662 1.222147 -0.738281

9 1 0 0.721780 1.252806 -1.688221

10 8 0 3.673629 0.431824 1.657234

11 1 0 3.618280 0.084213 2.557787

12 8 0 1.681866 -0.408804 3.185145

13 6 0 0.665984 -0.892730 4.047405

14 1 0 0.167793 -1.770242 3.621864

15 1 0 1.164650 -1.174626 4.973179

16 1 0 -0.078962 -0.118118 4.258626

17 6 0 -1.576238 0.524092 -0.480316

18 1 0 -2.308525 0.141553 0.246383

19 1 0 -1.849565 1.555803 -0.728174

20 8 0 -1.606914 -0.277137 -1.662907

21 6 0 -2.792223 -0.197424 -2.380890

22 1 0 -3.656558 -0.362947 -1.705462

23 8 0 -2.719673 -1.259381 -3.305570

24 6 0 -3.937280 -1.527052 -4.000690

25 1 0 -4.593710 -2.132770 -3.353173

26 6 0 -3.576456 -2.389602 -5.198692

27 1 0 -3.043636 -3.271320 -4.818682

28 1 0 -4.506351 -2.719331 -5.678737

29 8 0 -2.765730 -1.648199 -6.095234

30 1 0 -2.543614 -2.209226 -6.844859

31 6 0 -4.667472 -0.204013 -4.371540

32 1 0 -5.374326 0.062426 -3.574440

33 6 0 -3.007753 1.165379 -3.111435

34 8 0 -5.382281 -0.279388 -5.609222

35 1 0 -6.309802 -0.451657 -5.423417

36 6 0 -3.636399 0.913959 -4.477668

37 1 0 -2.851449 0.599518 -5.171825

38 8 0 -4.230255 2.133661 -4.907222

39 1 0 -4.680861 1.935493 -5.738115

40 1 0 -3.723923 1.765563 -2.529124

41 8 0 -1.780645 1.865475 -3.230284

42 1 0 -1.963033 2.648411 -3.765092

---------------------------------------------------------------------

**Compound (3-H)^-1^**

Input orientation:

---------------------------------------------------------------------

Center Atomic Atomic Coordinates (Angstroms)

Number Number Type X Y Z

---------------------------------------------------------------------

1 6 0 0.586419 0.116009 -0.428558

2 6 0 0.366854 -0.364380 0.878821

3 6 0 1.385319 -0.494163 1.799721

4 6 0 2.766835 -0.145400 1.491527

5 6 0 2.936379 0.364201 0.152606

6 6 0 1.901950 0.476959 -0.759435

7 1 0 -0.632933 -0.663573 1.192442

8 1 0 3.952510 0.641430 -0.120886

9 1 0 2.110432 0.849953 -1.762732

10 8 0 3.717497 -0.272142 2.313707

11 8 0 1.051729 -1.040018 3.037478

12 6 0 -0.526134 0.236202 -1.399270

13 1 0 -1.469306 0.490197 -0.889880

14 1 0 -0.321554 1.000886 -2.155156

15 8 0 -0.769493 -1.026844 -2.123977

16 6 0 -2.000415 -1.099448 -2.716185

17 1 0 -2.800255 -0.870040 -1.981115

18 8 0 -2.150894 -2.460641 -3.126314

19 6 0 -3.477326 -2.836625 -3.493534

20 1 0 -4.034944 -3.141275 -2.591843

21 6 0 -3.285222 -4.069498 -4.383271

22 1 0 -2.965604 -4.899929 -3.737849

23 1 0 -4.222617 -4.343469 -4.867210

24 8 0 -2.336025 -3.840521 -5.408594

25 1 0 -1.568512 -3.447332 -4.967650

26 6 0 -4.227361 -1.653602 -4.168149

27 1 0 -4.730962 -1.049823 -3.401702

28 6 0 -2.217013 -0.155531 -3.934135

29 8 0 -5.208329 -2.075081 -5.127748

30 1 0 -6.022291 -2.295210 -4.663798

31 6 0 -3.238011 -0.760602 -4.902658

32 1 0 -2.697784 -1.382797 -5.623946

33 8 0 -3.907338 0.303404 -5.577188

34 1 0 -4.600612 -0.115002 -6.103786

35 1 0 -2.639409 0.787943 -3.558480

36 8 0 -0.981255 0.088657 -4.587713

37 1 0 -1.165982 0.704418 -5.307073

38 6 0 1.450943 -0.266417 4.160802

39 1 0 2.527363 -0.093034 4.140524

40 1 0 0.920480 0.698965 4.181999

41 1 0 1.168426 -0.841324 5.048325

---------------------------------------------------------------------

**Compound 10**

Standard orientation:

---------------------------------------------------------------------

Center Atomic Atomic Coordinates (Angstroms)

Number Number Type X Y Z

---------------------------------------------------------------------

1 6 0 3.362047 2.595343 -0.195759

2 6 0 2.003772 2.659400 -0.537246

3 6 0 1.296274 3.837355 -0.350382

4 6 0 1.933756 4.978969 0.172446

5 6 0 3.276131 4.912557 0.511189

6 6 0 3.984025 3.722054 0.333091

7 1 0 1.523075 1.768742 -0.920395

8 1 0 3.750903 5.795831 0.924074

9 1 0 5.032034 3.680924 0.614926

10 8 0 1.231419 6.132902 0.352269

11 1 0 0.323864 5.967159 0.062412

12 8 0 -0.036739 4.030796 -0.630876

13 6 0 -0.782933 2.933281 -1.136662

14 1 0 -0.780181 2.095456 -0.432530

15 1 0 -1.801274 3.295141 -1.266625

16 1 0 -0.388784 2.596979 -2.100812

17 6 0 4.146840 1.330872 -0.443961

18 1 0 4.533840 1.317214 -1.474570

19 1 0 5.005813 1.266326 0.228861

20 8 0 3.292777 0.201879 -0.258661

21 6 0 3.686814 -0.999588 -0.859289

22 1 0 4.347455 -0.785890 -1.711764

23 8 0 2.491118 -1.589982 -1.344296

24 6 0 2.220467 -2.947064 -1.005115

25 1 0 2.979365 -3.595044 -1.474072

26 6 0 0.925494 -3.320577 -1.719321

27 1 0 1.085042 -3.220929 -2.792642

28 1 0 0.637984 -4.346911 -1.487371

29 8 0 -0.171336 -2.431204 -1.431601

30 6 0 2.297898 -3.256033 0.517318

31 1 0 2.629784 -4.300917 0.605573

32 6 0 4.395726 -1.921087 0.150107

33 8 0 1.115450 -3.058075 1.275386

34 1 0 0.442057 -3.704376 1.018173

35 6 0 3.362611 -2.371645 1.169993

36 1 0 2.860110 -1.478821 1.554378

37 8 0 4.066061 -3.032092 2.223386

38 1 0 3.472225 -3.104025 2.978752

39 1 0 4.799707 -2.798362 -0.379593

40 8 0 5.452799 -1.207555 0.754295

41 1 0 5.624011 -1.648884 1.597710

42 6 0 -1.162702 -2.855731 -0.631378

43 8 0 -1.179217 -3.940812 -0.089213

44 6 0 -2.227639 -1.794095 -0.494068

45 1 0 -1.741952 -0.905744 -0.076122

46 1 0 -2.553621 -1.501443 -1.497246

47 6 0 -3.417499 -2.224974 0.374188

48 1 0 -3.049543 -2.527185 1.357903

49 1 0 -3.877258 -3.115759 -0.062284

50 6 0 -4.443841 -1.122911 0.518613

51 6 0 -4.363155 -0.201195 1.563681

52 6 0 -5.292162 0.835626 1.674215

53 6 0 -6.308935 0.956837 0.739209

54 6 0 -6.406179 0.038417 -0.316587

55 6 0 -5.476355 -0.987873 -0.417346

56 1 0 -3.577348 -0.290712 2.307291

57 1 0 -5.224739 1.544894 2.495862

58 1 0 -5.582262 -1.689555 -1.238991

59 8 0 -7.280795 1.934413 0.750789

60 1 0 -7.178780 2.501151 1.522232

61 8 0 -7.406055 0.146624 -1.234394

62 1 0 -7.958118 0.901948 -0.991417

---------------------------------------------------------------------

**Compound (10-H)-1**

Standard orientation:

---------------------------------------------------------------------

Center Atomic Atomic Coordinates (Angstroms)

Number Number Type X Y Z

---------------------------------------------------------------------

1 6 0 -0.037743 -2.831031 0.519274

2 6 0 -1.049333 -2.132580 1.211865

3 6 0 -2.349259 -2.084941 0.740397

4 6 0 -2.733906 -2.713331 -0.502996

5 6 0 -1.700243 -3.450641 -1.139058

6 6 0 -0.397163 -3.496417 -0.652822

7 1 0 -0.781298 -1.613067 2.126896

8 1 0 -1.967787 -3.955251 -2.063841

9 1 0 0.361573 -4.047156 -1.207416

10 8 0 -3.917158 -2.608456 -1.002929

11 8 0 -3.378472 -1.428787 1.380045

12 6 0 -3.066494 -0.624268 2.490793

13 1 0 -2.305663 0.126841 2.246419

14 1 0 -3.989797 -0.112184 2.761774

15 1 0 -2.713749 -1.220024 3.345326

16 6 0 1.374447 -2.786284 0.993296

17 1 0 1.436939 -2.832581 2.090413

18 1 0 1.957623 -3.613358 0.578333

19 8 0 2.004177 -1.532099 0.578982

20 6 0 3.259218 -1.253928 1.111499

21 1 0 3.452797 -1.885729 1.987642

22 8 0 3.269084 0.092633 1.578186

23 6 0 3.936673 1.096124 0.821835

24 1 0 5.002228 1.098613 1.112621

25 6 0 3.394912 2.437149 1.330280

26 1 0 3.682066 2.549748 2.376058

27 1 0 3.796188 3.274195 0.754936

28 8 0 1.962954 2.475593 1.334667

29 6 0 3.964119 0.919760 -0.727576

30 1 0 4.910006 1.380123 -1.052480

31 6 0 4.364492 -1.448000 0.062111

32 8 0 2.885513 1.470459 -1.464174

33 1 0 2.836578 2.429778 -1.342231

34 6 0 4.017086 -0.562818 -1.120486

35 1 0 3.013520 -0.843171 -1.446759

36 8 0 4.967250 -0.837525 -2.161952

37 1 0 4.569471 -0.560235 -2.994319

38 1 0 5.331117 -1.140776 0.494532

39 8 0 4.426862 -2.809659 -0.315310

40 1 0 4.798631 -2.816116 -1.207633

41 6 0 1.298756 3.147468 0.372600

42 8 0 1.844637 3.866435 -0.440483

43 6 0 -0.162586 2.794066 0.434933

44 1 0 -0.188020 1.702796 0.341597

45 1 0 -0.532298 2.993934 1.445579

46 6 0 -1.070498 3.423204 -0.638084

47 1 0 -0.504094 3.521091 -1.567869

48 1 0 -1.359080 4.437257 -0.340758

49 6 0 -2.277402 2.533573 -0.849074

50 6 0 -2.195601 1.472492 -1.751957

51 6 0 -3.175927 0.479965 -1.791606

52 6 0 -4.253837 0.526480 -0.912817

53 6 0 -4.369917 1.626360 -0.039314

54 6 0 -3.396457 2.615139 -0.006100

55 1 0 -1.325794 1.384947 -2.397959

56 1 0 -3.083934 -0.374865 -2.451575

57 1 0 -3.492679 3.415962 0.723128

58 8 0 -5.199888 -0.432023 -0.795648

59 1 0 -4.733710 -1.364286 -0.821933

60 8 0 -5.431749 1.637347 0.822782

61 1 0 -5.876562 0.786905 0.654236

**WHOLE REFERENCES**

1. Shi Y, Wang W, Kang J, Shi Y, Jia Z, Wang Y, Su B, Yao S, Lin N, Zheng R (1999) Reaction of hydroxyl radical with phenylpropanoid glycoside and its derivatives by pulse radiolysis. Sci China C-Life Sci 42*:* 420-426.
2. Kennedy JF, Kumar H, Panesar PS, Marwaha SS, Goyal R, Parmar A, Kaur S (2006) Enzyme-catalyzed regioselective synthesis of sugar esters and related compounds. J Chem Technol Biotechnol 81: 866-876.
3. Shimoji Y, Tamura Y, Nakamura Y, Nanda K, Nishidai S, Nishikawa Y, Ishihara N, Uenakai K, Ohigashi H (2002) Isolation and identification of DPPH radical scavenging compounds in Kurosu (Japanese unpolished rice vinegar). J Agric Food Chem 50: 6501-6503.
4. Frisch MJ, Trucks GW, Schlegel HB, Scuseria GE, Robb MA, Cheeseman JR, Montgomery JJA, Vreven T, Kudin KN, Burant JC, Millam JM, Iyengar SS, Tomasi J, Barone V, Mennucci B, Cossi M, Scalmani G, Rega N, Petersson GA, Nakatsuji H, Hada M, Ehara M, Toyota K, Fukuda R, Hasegawa J, Ishida M, Nakajima T, Honda Y, Kitao O, Nakai H, Klene M, Li X, Knox JE, Hratchian HP, Cross JB, Bakken V, Adamo C, Jaramillo J, Gomperts R, Stratmann RE, Yazyev O, Austin AJ, Cammi R, Pomelli C, Ochterski JW, Ayala PY, Morokuma K, Voth GA, Salvador P, Dannenberg JJ, Zakrzewski VG, Dapprich S, Daniels AD, Strain MC, Farkas O, Malick DK, Rabuck AD, Raghavachari K, Foresman JB, Ortiz JV, Cui Q, Baboul AG, Clifford S, Cioslowski J, Stefanov BB, Liu G, Liashenko A, Piskorz P, Komaromi I, Martin RL, Fox DJ, Keith T, Al-Laham MA, Peng CY, Nanayakkara A, Challacombe M, Gill PMW, Johnson B, Chen W, Wong MW, Gonzalez C, Pople, JA (2004) Gaussian, Inc.: Wallingford CT.
5. Aligiannis N, Mitaku S, Tsitsa-Tsardis E, Harvala C, Tsaknis I, Lalas S, Haroutounian S (2003) Methanolic extract of *Verbascum macrurum* as a source of natural preservatives against oxidative rancidity. J Agric Food Chem 51: 7308-7312.
6. Ersöz T, Alipieva KI, Yalcin FN, Akbay P, Handjieva N, Dönmez AA, Popov S, Calis I (2003) Physocalycoside, a New Phenylethanoid Glycoside from*Phlomis physocalyx* Hub.-Mor. Z Naturforsch C 58: 471-476.
7. Delazar A, Sabzevari A, Mojarrab M, Nazemiyeh H, Esnaashari S, Nahar L, Razavi SM, Sarker SD (2008) Free-radical-scavenging principles from *Phlomis caucasica*. J Nat Med 62: 464-466.
